# Structural and evolutionary insights into the isoprene monooxygenases

**DOI:** 10.1093/femsec/fiag004

**Published:** 2026-01-22

**Authors:** Nasmille L Larke-Mejía, Leonardo de Oliveira Martins, John Colin Murrell

**Affiliations:** Center for Volatile Interactions (VOLT), Department of Biology, University of Copenhagen, Universitetsparken 15, Copenhagen Ø, 2100, Denmark; Signal Processing Group, Department of Electrical Engineering and Electronics, University of Liverpool, L69 7ZF Liverpool, United Kingdom; School of Environmental Sciences, University of East Anglia, Norwich NR4 7TJ, United Kingdom

**Keywords:** isoprene degradation, isoprene monooxygenase, IsoA, soluble di-iron monooxygenase, methane monooxygenase, protein sequence evolution, structural modelling, functional marker genes

## Abstract

Isoprene, a highly reactive biogenic volatile organic compound emitted by terrestrial vegetation, influences atmospheric chemistry but its microbial degradation remains poorly understood. Aerobic degradation begins with isoprene monooxygenase (IsoMO), a multicomponent di-iron monooxygenase encoded by the *isoABCDEF* cluster, with *isoGHIJ* supporting downstream steps. We analysed *iso* gene clusters from 11 confirmed isoprene degraders, reconstructed amino acid sequence phylogenies, and generated structural models of IsoMO components using mainly AlphaFold2. IsoA, IsoE, and IsoB formed a highly conserved α₂β₂γ₂ monooxygenase core (IsoMO core) whose predicted architecture and closely resembled the soluble methane monooxygenase hydroxylase, revealing a shared di-iron catalytic framework adapted to distinct hydrocarbon substrates. IsoA was the most conserved subunit and remains a reliable molecular marker for isoprene degradation. This work presents the first detailed structural model of an IsoMO core and reveals its deep relationship to other soluble di-iron monooxygenases. Together these results provide a molecular foundation for future mechanistic, ecological and inhibitor-based studies linking enzyme-level specificity to microbial control of isoprene turnover under changing climate conditions.

## Teaser

The evolutionary conserved di-iron architecture in isoprene monooxygenase (IsoMO) revealed by genomic and structural analyses highlights a widespread microbial process that regulates isoprene fluxes across natural ecosystems.

## Introduction

Isoprene (2-methyl-1,3-butadiene) is a highly reactive C₅ hydrocarbon and the most abundantly emitted non-methane biogenic volatile organic compound (BVOC), released primarily by terrestrial plants, with global emissions comparable to methane at rates exceeding ∼500 Tg per year (Guenther et al. 2012, reviewed in Carrión et al. 2020). In the atmosphere, isoprene plays a major role in the formation of tropospheric ozone and secondary organic aerosols (SOAs), which impact air quality, cloud properties, and radiative forcing (Claeys et al. 2004, Wennberg et al. 2018, Shen et al. 2024). While its atmospheric chemistry is well characterized, microbial degradation of isoprene is a new research area (reviewed in Dawson et al. 2023).

Recent evidence has shown that bacteria from diverse environments (soil, aquatic, and leaf surfaces) can degrade isoprene, making microbial degradation the only known biological sink of atmospheric isoprene (McGenity et al. 2018, Carrión et al. 2020). In some ecosystems, such as *Sphagnum*-dominated peatlands, microbial uptake of isoprene may even counterbalance net emissions from the moss (Crombie et al. 2025).

Aerobic isoprene degradation (*iso*-type, Supplementary Fig. S1) is initiated by isoprene monooxygenase (IsoMO) encoded by the *isoABCDEF* genes. IsoMO is a multicomponent di-iron monooxygenase enzyme from Group 1 of the soluble di-iron centre monooxygenase family (SDIMO) essential for the first step of isoprene degradation (Crombie et al. 2015, Dawson et al. 2020, Yang et al. 2025). This monooxygenation step yields (R)-epoxyisoprene (van Hylckama Vlieg et al. 2000), a toxic intermediate which is further processed and detoxified via a glutathione-dependent pathway encoded by *isoGHIJ* (Rix et al. 2023). The core *iso* gene cluster (*iso* cluster) typically comprises 11 genes (*isoA-isoJ, aldH*) (Dawson et al. 2023). Within IsoMO monooxygenase core (IsoMO core: α_2_β_2_γ_2_; Zhou et al. 1998, Leahy et al. 2003), IsoA encodes the central catalytic α-subunit component, which contains a di-iron active site (van Hylckama Vlieg et al. 1998) and IsoE and IsoB encode the associated β-subunit and γ-subunits, respectively (van Hylckama Vlieg et al. 2000). The IsoMO also contains IsoC (a Rieske-type ferredoxin), IsoD (a coupling protein), and IsoF (an FMN-containing reductase component) that extract electrons from NADH and transfers them to the di-iron center in the monooxygenase α-subunit (Zhou et al. 1998, van Hylckama Vlieg et al. 2000), as seen with other SDIMO such as methane and toluene monooxygenases (Rosenzweig et al. 1997, Notomista et al. 2003, Wang et al. 2014).

The detoxification module pathway includes first IsoI, an isoprene pathway-specific glutathione S- transferase (GST) (van Hylckama Vlieg et al. 1998, Van Hylckama Vlieg et al. 1999), which conjugates epoxyisoprene with glutathione (GSH) to produce HGMB (1-hydroxy-2-glutathionyl-2-methyl-3-butene) (Rix et al. 2023). IsoH (an NAD⁺-dependent dehydrogenase) which sequentially oxidizes HGMB in two NAD^+^-dependent steps to form GMBA (2-glutathionyl-2-methyl-3-butanoic acid) (Rix et al. 2023). IsoG (a putative CoA-transferase) then converts GMBA into GMBA-CoA by attaching coenzyme A (from an unknown source). In the same step IsoJ, encodes for the second isoprene pathway-specific GST assigned to the Actino-like class of GST (Haarmann et al. 2025), that removes oxidized glutathione (GSSG) from GMBA-CoA, producing MBE-CoA (2-methyl-3-hydroxy-butenyl-CoA, Dawson et al. 2022, Rix et al. 2023).The final gene in the cluster is *aldH* which encodes a putative aldehyde dehydrogenase thought to oxidize intermediates of HGMB to GMBA (Dawson et al. 2022) and likely catalyzes a second NAD^+^-dependent oxidation step as in the StyH/AldH1 system of styrene metabolism (Dawson et al. 2023, Rix et al. 2023).

Within the *iso* cluster, *isoA* is the most conserved gene and has been widely used as a molecular marker for detecting and anchoring *iso* cluster genes from isoprene degraders detected in environmental DNA using PCR and qPCR assays and metagenomics (El Khawand et al. 2016, Larke-Mejía et al. 2019, Carrión et al. 2020). IsoA shares ancestry with MmoX (Group 3 SDIMO, Yang et al. 2025), the catalytic α-subunit of soluble methane monooxygenase (sMMO), and with the toluene and alkene monooxygenases (Leahy et al. 2003, Coleman et al. 2006, McGenity et al. 2018). Although sMMO has been thoroughly characterized (Rosenzweig et al. 1993, Hakemian and Rosenzweig 2007), detailed structural insights into IsoA are lacking.

*Iso*-type degraders have been isolated from a wide range of environments yet the evolutionary history, modular organization, and structure-to-function relationships of the *iso* cluster are poorly understood and no systematic phylogenetic or structural comparison of the proteins encoded in the full *isoA–J, aldH* cluster has been performed. Also, the evolutionary congruence of individual proteins, patterns of duplication within the gene cluster, and the structural similarity of IsoA to other SDIMO α-subunits remain unexplored. To address this, we conducted a comparative genomic and structural analysis of the *iso* cluster in confirmed *iso*-type isoprene-degrading bacteria. Our aims were to: (i) visualise the gene order and modular structure of the core *iso* cluster; (ii) construct nucleotide and protein phylogenies to evaluate evolutionary congruence across the cluster; (iii) compare IsoA to representative SDIMO α-subunits to identify conserved functional motifs; and (iv) use AlphaFold2 monomer and multimer modelling and structural alignment to explore IsoA structure and active site architecture.

Anchored by the well-characterized sMMO framework, this study places IsoMO within the broader evolutionary landscape of microbial one-carbon (C_1_) and hydrocarbon monooxygenases. Our integrated approach provides the first detailed structural and phylogenetic comparison of IsoA with MmoX and lays the groundwork for future biochemical and ecological studies of isoprene metabolism. We hypothesized that: (i) phylogenetic signals within the *iso* cluster reflect vertical inheritance with taxon-specific modularity; (ii) IsoA is the most conserved and functionally constrained gene within the cluster, with structural features similar to MmoX; and (iii) IsoA and MmoX share conserved catalytic residues, reflecting mechanistic parallels in hydrocarbon oxidation.

## Methods

### Genome selection and dataset compilation

Eighteen bacterial genomes representing extant *iso*-type isoprene-degrading strains were identified, 15 Gram-positive strains, primarily from the genus *Rhodococcus* (e.g. *Rh*. AD45, *Rh. opacus* PD630, *Rh*. LB1, *Rh*. WS7), two *Gordonia* (*Gordonia* sp. i37 and *Gordonia* sp. OPL2), *Mycobacterium* sp. AT1, and *Nocardioides* sp. WS12 plus three Gram-negative representatives of the Comamonadaceae family (*Ramlibacter* sp. WS9, *Variovorax* sp. WS11, and *Sphingopyxis* sp. OPL5). From this dataset, 11 genomes were selected for downstream analyses based on published reports of isoprene catabolism, active enrichment in DNA-SIP experiments, and the presence of key catabolic genes, such as *isoA*. Assembly statistics (e.g. N50, L50, genome size, GC content, and supporting references) are provided in Supplementary Table S1. These 11 isolates also represent phylogenetic breadth, ecological diversity (soil, freshwater, and phyllosphere origins), and known variation in *iso* cluster structure (Table 1).

**Table 1:** Summary of all reported *iso-*type bacterial genomes. These isolates include confirmed and putative isoprene degraders from diverse habitats. Plant hosts are listed where known. The 11 genomes included in the main figures of this study are denoted by an asterisk (*). Sequences suppressed in GenBank due to genome size (^#^). All eleven sequences were included in supplementary analyses. Genome size and protein-coding gene count were obtained from NCBI assembly records.

Class	Isolate	Assembly accession	Reference	Source	Plant host	Genome size (Mb)	Protein-coding genes
*Actinomycetes*	*Rhodococcus* AD45 *	ASM94930v1 (Mar 2015)	(van Hylckama Vlieg et al. 1998)	freshwater	N/A	6.79 479	6029
*Actinomycetes*	*Rhodococcus opacus* PD630 *	ASM23433v1 (Nov 2011)	(Alvarez et al. 1996, Crombie et al. 2015)	soil	N/A	9.16 903	8942
*Actinomycetes*	*Rhodococcus* LB1	ASM158345v1 (Mar 2016)	(El Khawand et al. 2016)	leaf	*A.hippocastanum*	10.752	9270
*Actinomycetes*	*Rhodococcus* SC4 ^#^	ASM155547v1 (Mar 2016)	(El Khawand et al. 2016)	soil	N/A	10.5719	9123
*Actinomycetes*	*Rhodococcus* ACPA1 *	ASM230019v1 (Sept 2017)	(Crombie et al. 2017, 2018)	leaf	*Populus alba*	10.0608	8808
*Actinomycetes*	*Rhodococcus* ACPA4	ASM230018v1 (Sept 2017)	(Crombie et al. 2017, 2018)	leaf	*Populus alba*	7.06 712	6247
*Actinomycetes*	*Rhodococcus* ACS1	ASM230015v1 (Sept 2017)	(Crombie et al. 2017, 2018)	soil	*Salix fragilis*	10.8914	9508
*Actinomycetes*	*Rhodococcus* WS1 *	ASM379774v1 (Nov 2018)	(Larke-Mejía 2018)	soil	*Salix alba*	6.56 024	5943
*Actinomycetes*	*Rhodococcus* WS3	ASM379708v1 (Nov 2018)	(Larke-Mejía et al. 2019)	soil	*Salix alba*	6.85 616	6095
*Actinomycetes*	*Rhodococcus* WS4 ^#^	ASM654360v1 (July 2019)	(Larke-Mejía et al. 2019)	soil	*Salix alba*	12.7295	10 854
*Actinomycetes*	*Rhodococcus* WS7	ASM654361v1 (July 2019)	(Larke-Mejía et al. 2019)	soil	*Salix alba*	6.6282	6053
*Actinomycetes*	*Gordonia* i37 *	ASM204308v1 (Mar 2017)	(Acuña Alvarez et al. 2009, Johnston et al. 2017)	estuary	N/A	6.22 816	5486
*Actinomycetes*	*Gordonia* OPL2 *	ASM379782v1 (Nov 2018)	(Larke-Mejía et al. 2020)	leaf	*E. guineensis*	5.7959	5169
*Actinomycetes*	*Mycobacterium* AT1 *	ASM204309v1 (Mar 2017)	(Johnston et al. 2017)	estuary	N/A	7.07 238	6620
*Actinomycetes*	*Nocardioides* WS12 *	ASM1410886v1 (Aug 2020)	(Larke-Mejía et al. 2019, Gibson et al. 2020)	soil	*Salix alba*	5.17 107	4925
*Betaproteobacteria*	*Ramlibacter* WS9 *	ASM379776v1 (Nov 2018)	(Larke-Mejía et al. 2019)	soil	*Salix alba*	6.98 085	6517
*Betaproteobacteria*	*Variovorax* WS11 *	ASM1049924v1 (Mar2018) ASM301487v1 (Feb2020)	(Crombie et al. 2018, Larke-Mejía et al. 2019, Carrión et al. 2020)	soil	*Salix alba*	8.48 961	7789
*Alphaproteobacteria*	*Sphingopyxis* OPL5 *	ASM379777v2 (Sept 2020)	(Larke-Mejía et al. 2019, 2020)	leaf	*E. guineensis*	4.67 697	4418

### Identification and extraction of isoprene metabolic gene clusters

Gene clusters were identified in the genomes of 11 isoprene-degrading bacterial isolates. Genomic assemblies were obtained in FASTA format (.fna) from NCBI or locally-generated assemblies. Initial cluster coordinates were based on the NCBI GenBank public annotation and homology to known *isoA*-containing regions. Where required, complete contigs were searched using BLAST to identify the location of *isoA* homologues. To validate and refine cluster extraction, an IsoA protein sequence database was used as a query in a tblastn search against all genome contigs. Each genome was split into individual contig files using Biopython (Cock et al. 2009) and combined into a single nucleotide BLAST database using makeblastdb [NCBI BLAST+ suite v 2.12.0+, (Camacho et al. 2009)]. Matching contigs were filtered to retain the top-scoring *isoA*-containing regions, and from these contigs the whole identifiable *iso* operon was extracted with an additional ±2 kb of flanking sequence at each side of the operon (total region varied from 13 to 29 kb between isolates) and stored in a curated directory. Where needed, specific contigs from GenBank (e.g. NZ_RKME01000032.1 for *Gordonia* sp. OPL2) were targeted to extract the correct cluster. During trimming, GenBank records were validated to retain the molecule_type annotation and any features located entirely within the extracted region were retained and coordinate-shifted accordingly.

### Functional annotation, visualization, and comparative analysis

**Figure 1:**
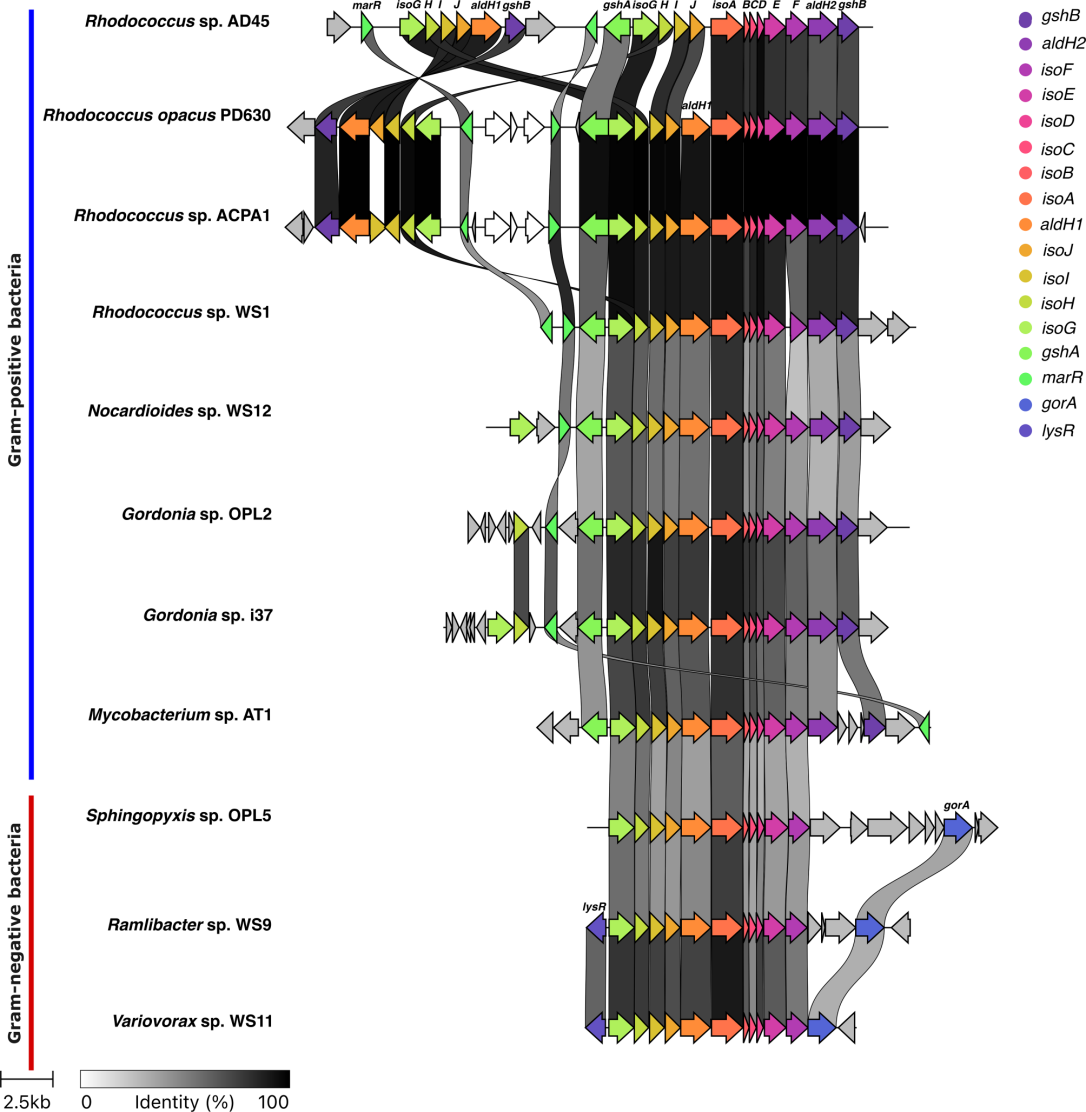
Organization of the *iso* gene clusters in 11 representative isoprene-degrading bacteria. *iso* clusters containing the *isoA* gene and associated operon components were extracted from annotated genomes. Homologous genes across strains are connected by grey links, with shading indicating amino acid identity (threshold: ≥20%). Genes are coloured and labelled according to putative functions: *isoA–isoJ* encode the IsoMO complex (including the center of the cluster *isoA-C*) and associated electron transfer proteins; *aldH1/2, gshA/B, gorA, marR*, and *lysR* represent adjacent genes putatively involved in regulation, redox processes, or glutathione metabolism. Strain names are shown on the left, with clusters scaled proportionally. Gene orientations reflect native operon direction. In Gram-positive strains, most *Rhodococcus* genomes harboured duplicated *isoGHIJ* modules flanking the monooxygenase genes, whereas Gram-negative strains showed a single contiguous operon. Notably, *Rh*. WS1 lacked the duplicated detoxification module, retaining only a single *isoGHIJ* copy, suggesting intra-lineage structural variability.

All curated *isoA* cluster regions were re-annotated using Bakta (v1.11.4, (Schwengers et al. 2021) against the full Bakta reference database (Zenodo, DOI: 10.5281/zenodo.7997310). Annotated .gbk files for each *iso* cluster were then visualized in Fig. 1 and compared across the 11 isoprene-degrading isolates using Clinker (v0.0.31; Gilchrist and Chooi 2021). Where necessary, whole cluster orientation and gene labels were manually curated to improve interpretability.

### *iso* cluster identification and comparative genomics

The 11 *iso* genes (*isoA-J, aldH*, referred to here collectively as the 11 *iso* genes) were identified in the selected genomes and cluster boundaries were defined by syntenic neighborhood, proximity, and functional annotations. Protein and nucleotide sequences were extracted for each gene and compiled into FASTA files for further analysis (Supplementary Table S2).

Pairwise sequence similarity was assessed using BLASTn (nucleotide) and BLASTp (protein) with default parameters. Percent identity matrices were generated for each gene (protein: Supplementary Table S3) and mean pairwise identity values were computed and grouped by Gram type (Gram+: Gram+, Gram-: Gram-, Gram+: Gram-), to quantify taxonomic divergence (Supplementary Table S4).

### Phylogenetic and sequence alignment analyses

DNA and predicted amino acid sequences for the 11 *iso* cluster genes from each of the 11 genomes (Table 1) were used to infer gene-by-gene phylogenies. Multiple sequence protein alignment was generated using MAFFT v7.505 (Katoh and Standley 2013) with the L-INS-i strategy and trimmed with trimAl v1.4 (Capella-Gutiérrez et al. 2009) using the auto mode. For each full protein alignment, maximum likelihood phylogenetic trees (Supplementary Fig. S2A–Supplementary Fig. S12A) were constructed with IQTREE2 v2.4.0 (Minh et al. 2020) under an LG evolutionary model (Le and Gascuel 2008) with gamma heterogeneity (Yang 1994). Branching support was assessed with 10 000 ultrafast bootstrap replicates as implemented in IQTREE2 (Hoang et al. 2018), where the best tree as well as each bootstrap replicate were further optimised using a nearest neighbor interchange search. The same procedure was used for the DNA alignment, under an HKY model (Hasegawa et al. 1985) with gamma heterogeneity (Yang 1994).

For visualisation and protein structure prediction purposes, the alignment was visualised with ESPript 3.0 (Robert and Gouet 2014) (Supplementary Fig. S2B–Supplementary Fig. S12B) with secondary structure elements annotated using *Rh* sp. AD45 as a reference for numbering. Conserved sequence motifs characteristic of di-iron monooxygenases motifs were identified manually and verified using UniProt (UniProt Consortium 2025) and PDB annotations (Zardecki et al. 2022).

To assess phylogenetic congruence among genes and input data source, normalised tree distances between all maximum likelihood tree pairs were calculated, including comparisons between amino acid-derived and nucleotide-derived trees. For these calculations, the phangorn library for R was used (Schliep 2011). Specifically, we calculated four distances between all tree pairs: the Robinson Foulds (RF) (Robinson and Foulds 1981) and the subtree prune and regraft (SPR) (de Oliveira Martins et al. 2008) normalised by their maximum values. These distances only take the topologies into account, and therefore to account also for branch lengths, we employed the weighted RF (Robinson and Foulds 1979) and the branch score distances (Kuhner and Felsenstein, 1994) on rescaled branch lengths, so that trees had comparable phylogenetic diversity. All distances were visualised as a heatmap to highlight clusters of phylogenetically similar histories and congruent genes, and their distribution was summarised in a histogram to assess the degree of overall topological similarity. Multidimensional scaling (MDS) was applied to the distance matrices using the cmdscale function in R v4.3.1 to project relationships among gene trees into two-dimensions, enabling visual detection of phylogenetically coherent gene modules. Figures were generated using ggplot2 v3.4.2 and ggtree (Yu et al. 2017) in R, and for visualisation purposes the trees were re-rooted by midpoint rooting using the phangorn library (Schliep 2011).

### Structural modelling of IsoMO subunits and complexes

Protein structure predictions were performed using AlphaFold2 implemented in ColabFold v1.5.2 (Mirdita et al. 2022), which integrates MMseqs2-based multiple sequence alignment (Steinegger and Söding 2017) and the AlphaFold2 inference pipeline (Jumper et al. 2021), using default parameters. For each of the 11 genes, monomeric models were generated for both Gram-positive (*Rh*. AD45) and Gram-negative *Variovorax* sp. WS11 (*V*. WS11) representatives (*Rh. opacus* PD630 was used in place of *Rh*. AD45 for AldH1). For IsoA, additional dimeric models were generated using the AlphaFold2 “auto” model_type pipeline to evaluate conserved α₂ arrangements. A larger α₂β₂γ₂ assembly was subsequently modelled using AlphaFold2-Multimer v3 model_type, by combining multimer predictions for IsoA + IsoB + 2 × IsoE and 2 × IsoA + 2 × IsoB within PyMOL using the super alignment command. This approach captured the canonical IsoMO core architecture (num_recycles = 1). The final IsoMO core structure was reconstructed and verified with AlphaFold3 (Abramson et al. 2024). Confidence scores (pLDDT, pTM, ipTM) for the modelled monomers/dimers and multimers were generated using AlphaFold2 or AlphaFold3, respectively.

Structural visualisation, chain splitting, superposition, and residue highlighting were performed using PyMOL v2.5 (Schrödinger and DeLano 2020). IsoA models were additionally aligned to the crystallographic structure of the sMMO α-subunit MmoX (PDB ID: 1MTY; Rosenzweig et al. 1993) after removal of non-α subunits. Iron-coordinating residues, conserved motifs, and clade-specific loops were highlighted and labelled manually for clarity.

### Comparison of alpha-subunit sequences of different SDIMOs

Protein sequences for the α-subunits of selected SDIMOs were compiled. IsoA sequences from the 11 isoprene degraders were included. A list of strains and accession numbers is provided in Supplementary Table S5. The dataset included six sMMO (sMMO; MmoX) sequences from *Methylosinus trichosporium* Ob3b, *Methylococcus capsulatus* (Bath), *Methylomonas methanica* MC09 (Boden et al. 2011, Zill et al. 2022), *Methylocella silvestris* BL2, *Methylocella tundrae* T4, and *Methylocella palustris* BL2; butane monooxygenase (BmoX) from *Pseudomonas butanovora*; tetrahydrofuran monooxygenase (ThmA) from *Pseudonocardia tetrahydrofuranoxydans*; propane monooxygenase (PrmA) from *Gordonia* sp. TY5 and *Rh*. sp. RR1; propane monooxygenase (PmoC) from *Mycobacterium* sp. M156; alkene monooxygenase (EtnC) from *Mycolicibacterium rhodesiae* JS60, *Mycolicibacterium chubuense* NBB4, and *Nocardioides* sp. JS614; toluene ortho-monooxygenase (TmoA3) from *Burkholderia cepacia* G4; toluene-4-monooxygenase (TmoA) from *Pseudomonas mendocina* KR1; toluene-3-monooxygenase (TbuA) from *Ralstonia pickettii* PKO; and alkene monooxygenase (XamoA) from *Xanthobacter autotrophicus* Py2.

**Figure 2:**
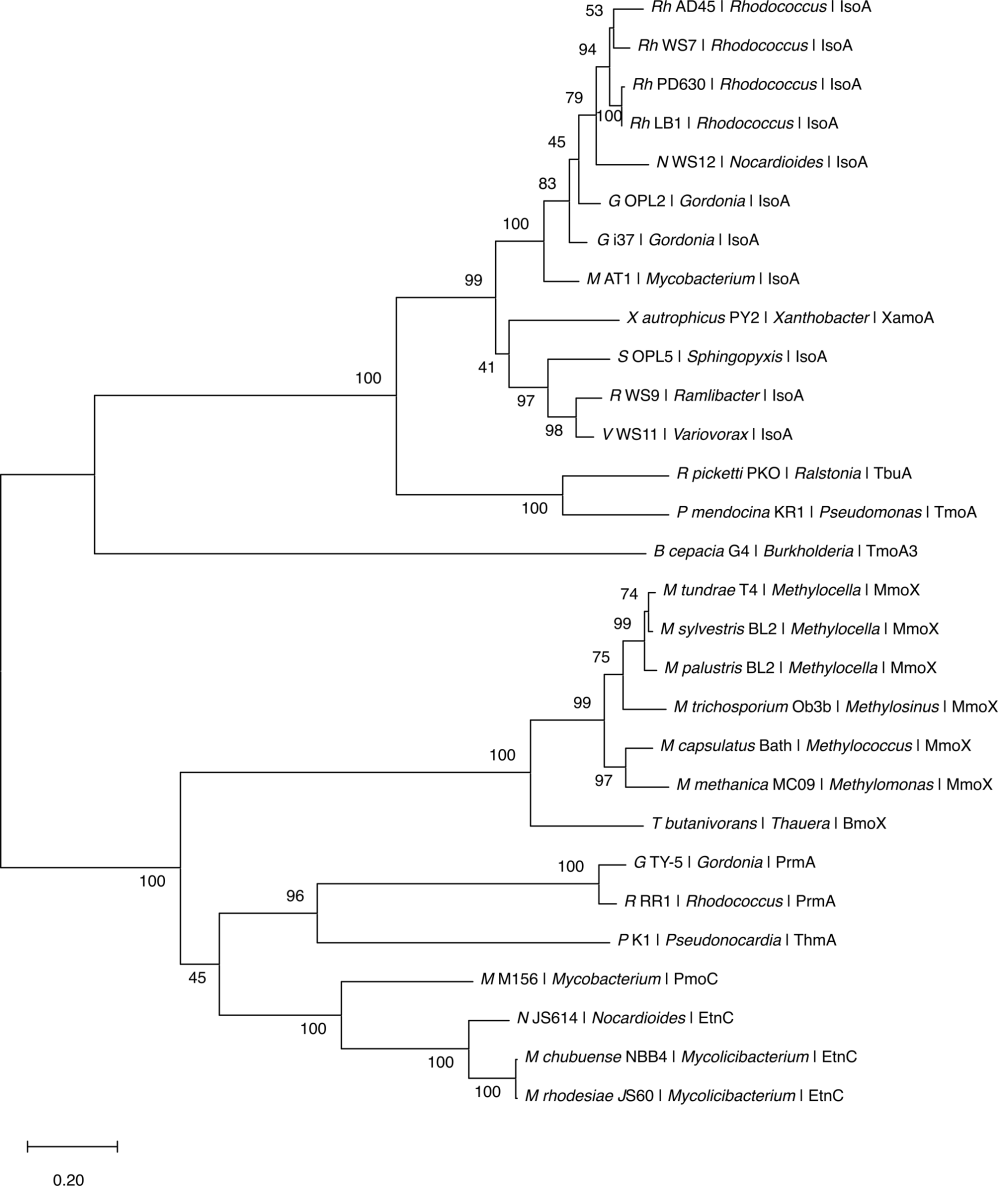
SDIMO α-subunit phylogeny and placement of IsoA. Maximum-likelihood phylogeny of soluble di-iron monooxygenase (SDIMO) α-subunits showing the placement of IsoA sequences from 11 confirmed isoprene degraders (Gram-positive and Gram-negative) relative to representative SDIMO groups. One to two representative sequences were retained per major SDIMO lineage (BmoX, PrmA, ThmA, TmoA) and six MmoX sequences, together with all IsoA sequences included in this study. IsoA forms a distinct, well-supported clade separated into Gram-positive (*Rhodococcus, Gordonia, Mycobacterium, Nocardioides*) and Gram-negative (*Variovorax, Sphingopyxis, Ramlibacter*) subclades. The analytical procedure encompassed 29 amino acid sequences with 617 positions in the final dataset. Scale bar represents amino acid substitutions per site. Accession numbers and full taxa are provided in Supplementary Table S5.

Multiple sequence alignment and maximum-likelihood trees were performed as described previously. The alignment (Supplementary Fig. S16) has *Rh*. AD45 IsoA as the structural reference for secondary structure annotation and motif mapping. For the main SDIMO context tree (Fig. 2), all IsoA sequences were retained along with one to two representative sequences per major SDIMO α-subunit clade (MmoX, BmoX, PrmA, ThmA, TmoA) to highlight relationships without overloading the topology.

### Residue-level comparison between IsoA and MmoX

**Figure 3:**
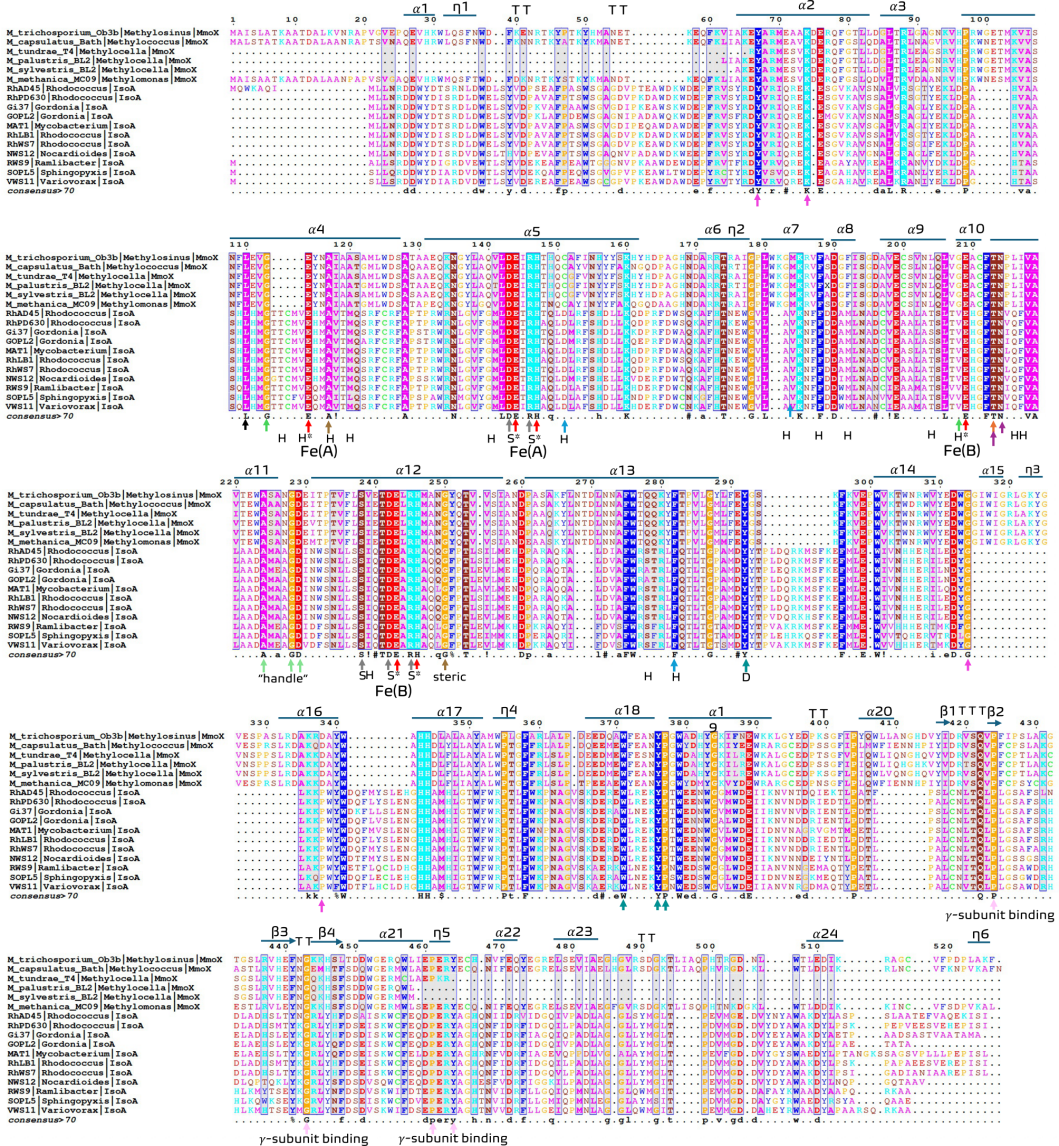
Protein sequence comparison of IsoMO α-subunit (IsoA) and sMMO α-subunit (MmoX). Multiple sequence alignment of MmoX and IsoA α-subunits visualised with ESPript 3.0, using *Rh*. AD45 IsoA as the structural reference for residue numbering and secondary structure annotation. Arrows indicate known or putative features of IsoA/MmoX (1) iron binding ligand residue in red, (2) implicated in methane oxidation in blue, (3) gating residue in black, (4) residues which might confer enantioselectivity in oxidation products in dark green, (5) highly conserved residue in orange, (6) hydrogen bonding (C-F helices) in grey, (7) delivery of electrons to the active site in purple, (8) tighly packed regions in brown, (9) canyon residues for protein B or oxidoreductase, (10) handle residues in light green, (11) important docking residues in teal and the letter D, and (12) residues that interacts with the gamma subunit in pink, according to Leahy *et al* (Table 2). Abbreviations of the annotated residues: H, hydrophobic residues surrounding the diiron center; S, structural residues involved in hydrogen bonding of the alpha-helices A and C; *, iron cluster ligands; D, docking residues; Fe**(A)**, iron cluster ligands for Fe**(A)** iron center; Fe**(B)**, iron cluster ligands for Fe**(B)** iron center. Other labels describe the location or possible function of the residue.

A focused alignment was constructed between the IsoA and MmoX sequences using MAFFT v7.505 (without trimming). Structural superpositions of IsoA monomers and dimers with MmoX (PDB: 1MTY) were performed with PyMOL. Conserved secondary structural elements were inferred by aligning six representative MmoX sequences and visualized via ESPript, enabling cross-reference of α-helices, β-strands, and di-iron motifs between MmoX and IsoA (Fig. 3; Supplementary Fig. S17 and Supplementary Fig. S18).

## Results

### Overview of *iso*-genes and genomic datasets

To investigate the diversity, conservation, and genomic context of the IsoMO and its associated catabolic genes, we constructed a dataset of *iso* clusters from 11 confirmed bacterial genomes of *iso*-type isoprene degraders from multiple taxonomic groups originating from diverse environments. These included eight Gram-positive strains, primarily from the genus *Rhodococcus*, along with representative *Gordoni*a, *Mycobacterium*, and *Nocardioides*, and the three Gram-negative isoprene degraders from the family Comamonadaceae: *Ramlibacter* WS9, *Variovorax* WS11, and *Sphingopyxis* OPL5.

Although relatively few genomes are available, the dataset reflects broad taxonomic and ecological diversity, with isolates recovered from estuarine, freshwater, soil, and phyllosphere environments. Most strains were obtained from enrichment experiments, using isoprene as the only source of carbon and energy, often from leaves or soils associated with isoprene-emitting trees such as *Elaeis guineensis, Populus alba*, and *Salix alba*. Following enrichment, bacteria isolates were purified and confirmed to degrade isoprene through growth assays, along with detection of key *iso* genes (initially *isoA)*. The taxonomic affiliation, source of isolation, and relevant references for each strain are summarized in Table 1, with detailed genome statistics provided in Supplementary Table S1. Genome sizes ranged from 4.68 to 12.7 Mb, and protein-coding genes varied from 4400 to 10 800 per genome.

### Organization and conservation of the *iso* gene cluster

Syntenic neighbourhood comparisons separate Gram-positive and Gram-negative taxa (Fig. 1). Homologous genes were identified by amino acid identity (≥20%) and gene orientation was maintained in the figure to reflect the native genomic organization and orientation of each gene. As expected, all strains contained a conserved modular cluster of six genes encoding the IsoMO subunits (*isoA-F)*, typically followed by four glutathione-dependent genes (*isoG-J)*. The aldehyde dehydrogenase gene (*aldH1*), was located immediately downstream of *isoF* in most genomes, except for *Rh*. AD45 in which *aldH1* was absent from this locus (Dawson et al. 2020).

Two major patterns were apparent from the *iso* cluster visualization. First, most *Rhodococcus* genomes (except WS1, isolated at lower isoprene enrichment concentrations) showed a distinctive modular organization characterized by duplications and rearrangements of the *isoGHIJ* module. This suggests possible species-specific adaptation to environmental conditions or isoprene flux. Second, Gram-positive and Gram-negative strains may be further distinguished by adjacent accessory genes. In Gram-positives (*Rhodococcus, Nocardioides, Gordonia, Mycobacterium*), *gshA* was consistently positioned upstream of *isoG, gshB* downstream of *isoF* also observed by Haarmann et al. 2025, and *marR*-like regulator was usually present (absent only in *Mycobacterium*, which instead encoded MCE family proteins and LysR-type regulator). In Gram-negatives (*Sphingopyxis, Ramlibacter, Variovorax*), *gorA* (in charge of catalyzing glutathione disulfide to reduced glutathione; UniprotKB P06715) was present upstream of *isoF*, and two of the genomes also carried a *lysR*-type regulator gene.

### Pairwise amino acid sequence conservation of IsoMO and detoxification proteins

To further characterize the proteins encoded by the *iso* cluster, we calculated average amino acid sequence lengths across the 11 selected genomes (Supplementary Table S2). The IsoMO proteins were highly consistent in length, with IsoA averaging 425 amino acids (± 1), IsoB 334 (± 2), IsoC 184 (± 1), IsoD 194 (± 3), IsoE 381 (± 2), and IsoF 326 (± 2). By contrast, glutathione-dependent detoxification protein sizes were more variable, with IsoG averaging 177 amino acids (± 6), IsoH 206 (± 7), IsoI 219 (± 18), and IsoJ 225 (± 18). The adjacent aldehyde dehydrogenase (AldH1), was highly consistent at 499 amino acids (± 0).

Pairwise BLASTp comparisons were performed for each protein, with identity values grouped by taxonomic comparison: Gram-positive vs. Gram-positive (G⁺: G⁺), Gram-negative vs. Gram-negative (G⁻: G⁻), and Gram-positive vs. Gram-negative (G⁺: G⁻) (Supplementary Table S3-Supplementary Table S4). IsoA emerged as the most conserved protein, with identities of 82.4%–100% (G⁺: G⁺) and 81–100% (G⁻: G⁻). Even across Gram groups, conservation remained high (71.2%–74.6%), confirming its suitability as both a functional and phylogenetic marker. In contrast, the IsoMO core β-subunit and reductase (IsoE and IsoF, respectively) were among the least conserved, particularly in inter-group comparisons, with G⁺: G⁻ identities of 47.3%–54.1% and 39.3%–47.7%, respectively. The detoxification enzymes IsoG, IsoH, IsoI, and IsoJ also exhibited substantial divergence across Gram groups, with minimum inter-group identities ranging from 45.4% to 58.2%. AldH1 displayed intermediate conservation, with identities of 63.6%–100% (G⁺: G⁺) and 49.2%–54.1% (G⁺: G⁻), consistent with a less central or more functionally flexible role.

Overall, these comparisons show the center of the *iso* cluster (IsoA–C) is highly conserved across lineages, likely reflecting a strong purifying selection against detrimental mutations, contrasted by higher variability in the detoxification module. Nucleotide-level comparisons (Supplementary Table S6) mirrored these trends but showed greater variability, likely due to synonymous substitutions and divergence in non-coding regions.

### Phylogenetic analysis and alignment of proteins encoded by the *iso* cluster

To assess whether conservation patterns across the proteins encoded within the *iso* cluster reflect vertical inheritance, modular evolution, or recombination, we constructed maximum likelihood phylogenies based on amino acid sequences from proteins IsoA–F, AldH, and IsoG–J from the selected genomes (Supplementary Fig. S2A–Supplementary Fig. S12A). In all cases, Gram-positive (G^+^) and Gram-negative (G^-^) amino acid sequences resolved as distinct, well-supported clades, consistent with long-term lineage-specific divergence, with pairwise amino acid identity values (described previously, Supplementary Table S3) confirmed these patterns, G⁺: G⁻ comparisons consistently showing lower amino acid sequence identity than within-group comparisons.

Protein analyses are summarized below, with phylogenetic trees (A), multiple sequence alignment (B), and AlphaFold models (C) shown in Supplementary Fig. S2–Supplementary Fig. S12. The supplementary notes summarise the main phylogenetic and amino acid sequence alignment features for each protein, with G⁺/G⁻ variation profiles highlighted and conserved motifs referenced to *Rh*. AD45 numbering from ESPript.

Structural models generated for each IsoMO protein from both *iso*-type model organisms (*Rh*. AD45, Gram-positive and *V*. WS11, Gram-negative) which consistently predicted well-conserved structural scaffolds across all components (Supplementary Fig. S2C-Supplementary Fig. S12C). Structural overlays confirmed that the IsoMO multicomponent core, elements α₂β₂γ₂ (that include key α-helical and β-sheet elements) are retained across clades. Structural variation between clades was mostly found in loop insertions, termini, and surface-exposed segments, particularly in detoxification and electron transfer proteins. These results provide a consistent picture: the proteins at the center of the IsoMO (encoded by genes in the center of the *iso* cluster: IsoA–C) are highly conserved across all clades and retain functional motifs essential for di-iron catalysis and subunit assembly. Accessory proteins such as IsoG–J exhibit greater divergence and signs of duplication in Gram-positive taxa, while electron transfer components IsoD–F show intermediate variability, particularly in loop regions and termini. AldH1 appears loosely linked, phylogenetically intermediate, and structurally conserved, possibly reflecting its more peripheral role in the catabolic pathway.

### Pairwise topological comparisons between *iso* gene trees

To quantify the degree of phylogenetic congruence among the *iso* genes, we computed pairwise distances between gene trees using four metrics: normalized Robinson–Foulds (RF), weighted RF, normalized subtree prune-and-regraft (SPR), and branch score distance. These metrics capture both topological similarity and evolutionary rate variation. We focus on amino acid–based trees (Supplementary Fig. S13), as the nucleotide trees yielded comparable results but with greater uncertainty (Supplementary Fig. S14).

Heatmaps showed the IsoMO core structural genes *isoA, isoB*, and *isoE*, exhibited a high congruence across all metrics, forming a tightly coherent cluster in both RF and SPR analyses (Supplementary Fig. S13 and Supplementary Fig. S15; de Oliveira Martins et al. 2016). The same can be observed between the reductase component *isoF* gene and the structural gene *isoE*. In contrast, detoxification genes *isoG, isoH, isoI*, and *isoJ* showed higher pairwise distances, particularly in normalized RF and SPR metrics, consistent with their history of duplication, diversification, and modular evolution. They tend to cluster together on an MDS map, however, probably due to similar (alternative) evolutionary trajectories (Supplementary Fig. S15). *aldH1* and particularly *isoE* consistently showed partial congruence with the IsoMO genes, supporting *aldH1’*s intermediate evolutionary linkage.

Histogram distributions of RF distances (Supplementary Fig. S14) show how both amino acid and nucleotide alignment yield broadly similar tree-to-tree distance patterns across all metrics. Overall values were higher for nucleotide- than for amino acid–based trees, indicating slightly more diverse nucleotide-based phylogenies. In all cases, most comparisons fall at intermediate distance values, with only a small number of very similar tree pairs.

These tree-based comparisons reinforce the modular organization of the *iso* cluster. The comparatively low diversity of the monooxygenase components supports the hypothesis that the IsoMO core and the detoxification module follow separate evolutionary trajectories. The intermediate positioning of *isoD–F* and *aldH1* supports a mixed evolutionary trajectory, with components shaped by both functional linkage and independent adaptation.

### IsoA and its phylogenetic position with reference to α-subunits of other SDIMOs

To place IsoA within the broader context of SDIMO diversity, we constructed a maximum-likelihood phylogeny using representative α-subunits from well-characterized SDIMO families, including methane, alkene, toluene, and propane monooxygenases. IsoA sequences from all 11 isoprene-degrading genomes formed a monophyletic clade, distinct from other SDIMO lineages (Fig. 2; Supplementary Fig. S16). Within the IsoA clade, Gram-positive and Gram-negative sequences segregated into sister subgroups with strong bootstrap support, reflecting vertical inheritance shaped by host taxonomy. MmoX, the α-subunit of sMMO, formed a separate lineage within the tree, underscoring the evolutionary divergence between methane- and isoprene-oxidizing systems. This topology supports a single IsoA lineage specialized for isoprene oxidation and structured primarily by host phylogeny, consistent with vertical inheritance within Gram groups and substrate-specialized lineage separation within the SDIMO superfamily.

### Structural comparison of IsoA and MmoX reveals conservation of important residues

To investigate structural conservation between isoprene and methane monooxygenases, we aligned IsoA models with the crystal structure of MmoX from *Methylococcus capsulatus* Bath (PDB: 1MTY; Rosenzweig et al. 1997). Multiple sequence alignment revealed that all six residues responsible for di-iron coordination in MmoX (e.g. E114, H147, E243) were conserved in IsoA (e.g. E110, H143, E237), suggesting preservation of the catalytic core architecture (Fig. 3; Table 2; Supplementary Fig. S17–Supplementary Fig. S18). The functional roles of these residues in IsoMO are putative and inferred from structural homology rather than experimental verification; no site-directed mutagenesis or biochemical data currently confirm their activity. Nevertheless, the strong structural and sequence correspondence provides a compelling *in silico* framework to guide future mutagenesis and mechanistic studies of isoprene oxidation.

**Table 2:** Functionally important residues in MmoX vs. IsoA, mapped according to *Methylosinus trichosporium* Ob3b (MmoX) and *Rhodococcus* sp. AD45 (IsoA) sequence numbering. **Note:** The functional roles of IsoA residues are putative, based solely on in silico structural and sequence homology with MmoX (PDB 1MTY). These predicted correspondences identify candidate positions for future site-directed mutagenesis to verify catalytic and structural roles within the IsoMO di-iron centre.

Residue function	MmoX	IsoA
Gating	L110	L101
Enantioselectivity	G113, G208	G104, V202
Important in methane oxidation	C151, M184, F282	D147, V178, F274
Highly conserved	T213	T207
Iron ligand	E114, E144, H147, E209, E243, H246	E110, E140, H143, E203, E237, H240
Hydrogen bonding between C and F helices	D143, R146, S238, D242, R245	D139, R142, S232, D236, R239
Access of substrates and release of products to and from the active site	T213, N214, E240	T207, N208, Q234
Tightly packed regions of the protein	A117, G250	A113, G244
Canyon	Y67, K74, L321, G325, P329	Y64, K71, G315, P319
Handle	A224, G228, D229	A218, G222, D223
Possible docking	Y292, W371, Y376, P377	Y284, W362, Y367, P368
Interact with gamma	P424, G443, P461, Y464	P408, G427, Y430

**Figure 4:**
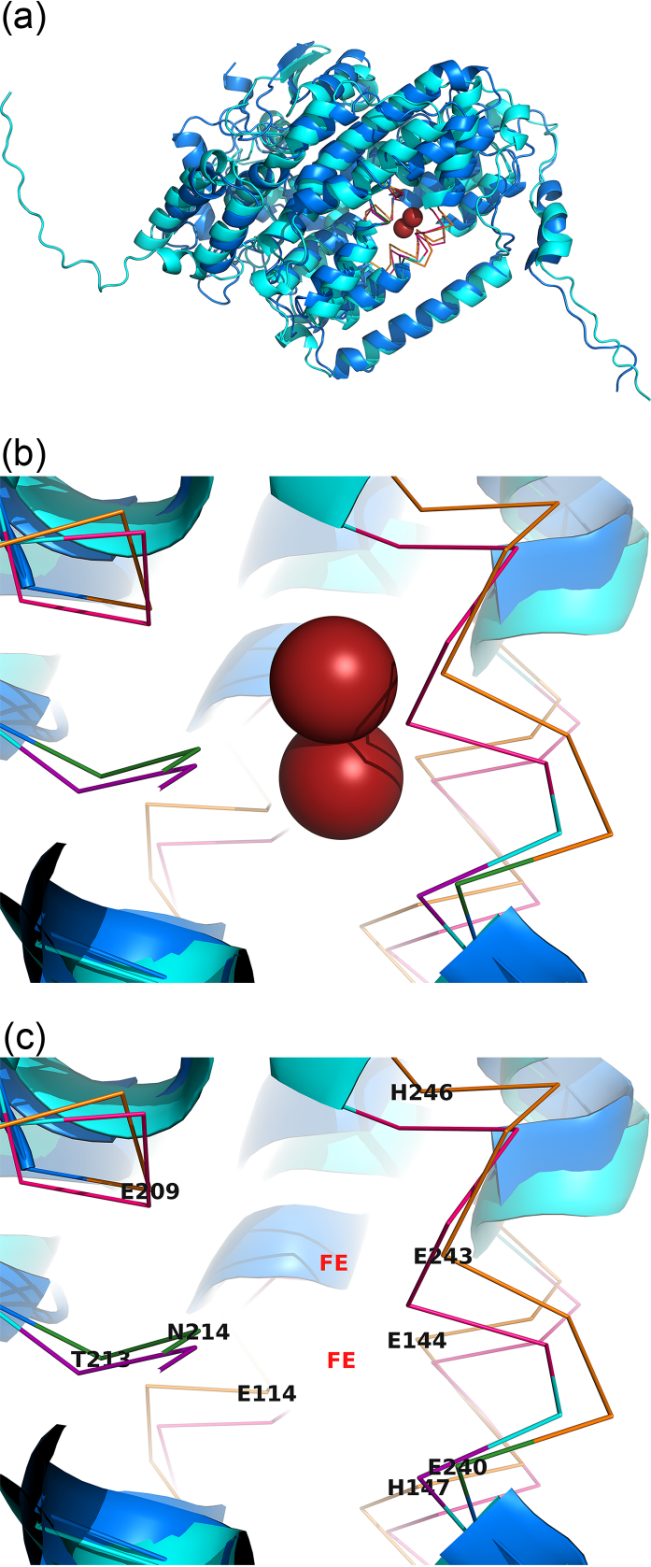
Structural comparison of IsoA and MmoX α-subunits. Two views of the cartoon representation of the α-subunit of IsoMO (IsoA, *Rh*. AD45) predicted by AlphaFold2 and the α-subunit of sMMO (MmoX, *Methylococcus capsulatus* Bath, PDB: 1MTY) superimposed and visualised in PyMOL. Residues involved in iron coordination and active site channel are highlighted in ribbon representation and detail annotated with residue type and position for MmoX (F4C). Location of iron atoms (spheres in F4B, letters FE in F4C) are shown in reference to sMMO in SF17 and the sMMO from 1MTY PDB model. Two magnifications and slight changes in orientation of the active site are shown to illustrate the conservation of the non-heme di-iron catalytic core and surrounding helices. The figure highlights both the conserved channel residues (TN) and EXXH motifs along with clade-specific substitutions near the active site, consistent with shared SDIMO ancestry but functional divergence for isoprene oxidation. Mean pLDDT give an overall confidence score of ∼91.8.

Protein structural overlays of IsoA (*Rh*. AD45) and MmoX showed high similarity in their α-helical cores and active-site configuration (Fig. 4A–C). Differences were observed in loop regions near the substrate-binding site and at the N-/C-termini, including substitutions in residues predicted to be in line with channel for entry of substrates and exit of products (e.g. Q234 in IsoA vs. E240 in MmoX), which may contribute to isoprene specificity. Protein structure models of IsoA achieved high overall confidence (mean pLDDT ≈ 91.8), with the α-helical core and di-iron ligating motifs resolved at very high confidence (>95). Lower scores (<70) were confined to terminal extensions and surface-exposed loops, consistent with their structural variability. Predicted alignment error (PAE) values were uniformly low within the catalytic core, supporting the reliability of the structural comparison with MmoX. Together, these features indicate that IsoA retains the hallmark SDIMO fold and di-iron catalytic geometry while exhibiting clade-specific divergence in substrate-binding regions.

### IsoA dimer modeling and clade-specific conservation

Multimer predictions for IsoA from *Rh*. AD45 and *V*. WS11 yielded stable dimeric assemblies with symmetric head-to-tail arrangements. The predicted interface was dominated by conserved α-helical contacts, and the overall fold was highly consistent across both clades (Supplementary Fig. S19). Structural superposition of the two dimer models showed near-complete alignment of core helices and di-iron-binding residues, with minor differences restricted to loop extensions and termini. Model confidence was high, with the *Rh*. AD45 IsoA dimer achieving a mean pLDDT of 90.8 (median 95.4), with over 80% of residues scoring above 90. Predicted template modeling scores (pTM = 0.86) and interface scores (ipTM = 0.83) supported a reliable dimer arrangement. Predicted alignment error (PAE) values were low within monomers (mean ∼6 Å) and moderate at the interface (median ∼8.3 Å), consistent with confident core contacts and some flexibility in loop regions. Together, these results suggest that IsoA dimerization is a structurally conserved feature of IsoMO systems and likely critical for maintaining the catalytic configuration across diverse isoprene degraders.

### Structural modelling of the IsoMO putative catalytic core

**Figure 5:**
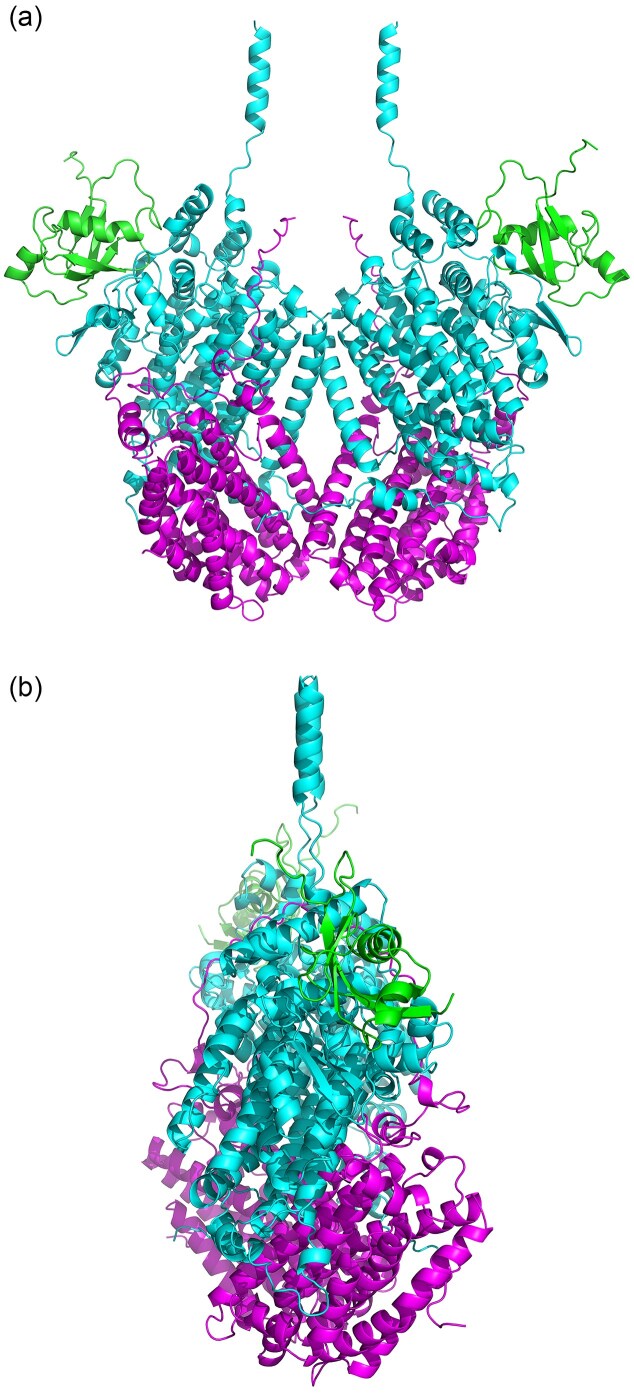
Predicted architecture of the IsoMO catalytic core. Two views of the quaternary structure of the IsoMO hydroxylase core from *Rh*. AD45. Multimer recovered the expected α₂β₂γ₂ oxygenase assembly, comprising an IsoA dimer, two IsoE subunits, and two IsoB subunits, closely resembling the architecture of other soluble di-iron monooxygenase (SDIMO) hydroxylases. Model confidence was very high overall with most residues scoring above 90. The core fold was strongly supported (pTM = 0.89), while inter-subunit interfaces showed high confidence (ipTM = 0.86), consistent with a stable, well-defined catalytic assembly and limited flexibility in peripheral contact regions.

AlphaFold3 multimer predictions for the IsoMO from *Rh*. AD45 recovered the expected α₂β₂γ₂ IsoMO core assembly comprising two IsoA (α; cyan), two IsoE (β; purple), and two IsoB (γ; green) subunits (Fig. 5). Conserved α-helical bundles within IsoA form the di-iron active site, while the β- and γ-subunits provide peripheral stabilising contacts consistent with the SDIMO. The IsoMO core was modelled with high global and interface confidence (pTM = 0.88; ipTM = 0.86 for *Rh*. AD45), indicating a reliable subunit orientation and well-defined assembly interface (Jumper et al. 2021, Abramson et al. 2024). Residue-level confidence was also high for conserved regions (pLDDT > 90), confirming a robustly folded catalytic scaffold. Lower scores (<50) were limited to short terminal or surface-exposed regions, consistent with expected flexibility in non-core segments.

Structural superposition of the IsoMO core with the sMMO hydroxylase from *Methylococcus capsulatus* Bath (PDB: 1MTY) revealed strong overall correspondence specifically between the α₂β₂ architectures, including alignment of conserved α-helical bundles and di-iron centres (Supplementary Fig. S20, refer to IsoA residues in Fig. 4). Together, these results provide a high-confidence structural framework for the IsoMO core.

## Discussion

This study used isolate-based genomic and structural comparison analyses to investigate the evolution and function of IsoMO across diverse bacterial taxa. By addressing key questions in microbial ecology (Antwis et al. 2017), we provide insights into microbial evolutionary processes and functional diversity underlying isoprene degradation. Protein phylogenetic and comparative genomic analysis support strong conservation of the IsoMO catalytic core alongside lineage-specific variation in accessory and detoxification genes. Together, these patterns support the view that IsoMO is maintained as a vertically inherited, functionally constrained enzymatic module, with surrounding regulatory and redox components exhibiting greater evolutionary flexibility, potentially in response to host-specific ecological and physiological pressures.

Structural modelling reinforces this interpretation. Despite sequence divergence between Gram-positive and Gram-negative taxa, the IsoMO core retains a conserved multicomponent architecture characteristic of Group1 soluble di-iron monooxygenases. IsoA carries a highly conserved di-iron center closely resembling MmoX (Rosenzweig et al. 1997). This structural continuity supports the view that sMMO and IsoMO arose either from a shared ancestral monooxygenase, as suggested by Leahy et al. (2003), or evolutionary convergence, with both enzymes subsequently adapting to distinct carbon substrates. IsoA was consistently the most conserved subunit, supporting its role as the evolutionary anchor of the *iso* cluster and the principal determinant of isoprene oxidation (Crombie et al. 2015, Carrion et al. 2018). Substrate-channel differences relative to MmoX (e.g. G208, C151, M184, E240 versus V202, D147, V178, Q234) may underpin isoprene specificity (Merkx et al. 2001, Borodina et al. 2007) and provide a framework for targeted mutagenesis and mechanistic studies.

From an ecological perspective, this modular organization may facilitate its persistence across diverse environments and host taxa. Isoprene-degrading and methane-oxidising bacteria have been reported in plant-associated habitats such as the phyllosphere of *Sphagnum* moss (e.g. *Methylocella* spp., Dedysh et al. 2005, Crombie et al. 2025). Because *Sphagnum* hosts active degraders of both methane and isoprene, it represents a natural environment in which the evolutionary history of these monooxygenases may converge at the interphase between aerial and submerged tissue, supporting the analysis and comparison of IsoA to MmoX and for exploring how related monooxygenases contribute to carbon cycling.

Within this ecological context, genomic and functional patterns may also help explain observations from microcosm studies. In *Sphagnum* moss, isoprene degradation was inhibited by 1-octyne but not by acetylene (Dawson et al. 2020, Sims et al. 2023, Crombie et al. 2025), consistent with a target within the conserved IsoMO active site. Structural models therefore provide a framework for interpreting such inhibition and for developing future imaging or probe-based approaches to study IsoMO activity *in situ*. Future studies should examine how substrates and inhibitors interact with IsoMO at the structural and molecular level, how variation in these interactions influences the microbial community adaptation and plant–microbiome interactions under environmental change.

Beyond these ecological implications, our phylogenetic analyses also help clarify long-standing misannotation issues, which likely arise from the limited representation of verified *iso* cluster sequences in public databases (Dawson et al. 2023). Moving forward, a combined strategy that couples direct phenotypic assays with indirect, sequence-based inference of active *iso*-type degraders (Larke-Mejía et al. 2019, Ledford et al. 2025) will be essential for improving database accuracy and strengthening ecological and evolutionary analyses. IsoA grouped closely with TmoA- and XamoA-like α-subunits (Dawson et al. 2020), indicating that further comparative work on these related SDIMOs will be essential for identifying residues that differentiate substrate selection across isoprene-, toluene-, and alkene-oxidizing enzymes.

Overall, these structural and genomic insights highlight that IsoMO represents a conserved and stable microbial function with direct ecological significance. Because isoprene degradation is the only known biological sink of one of the most abundantly produced BVOCs, understanding the evolutionary stability and mechanistic basis of IsoMO may be critical for predicting how microbial communities influence isoprene fluxes, plant–microbe interactions and VOC-driven ecosystem processes.

## Conclusion

This study presents an integrated evolutionary and structural analysis of IsoMO from confirmed isoprene-degrading isolates. We demonstrate that IsoMO comprises a highly conserved α₂β₂γ₂ di-iron monooxygenase core, closely related to the sMMO hydroxylase, embedded within a more flexible accessory framework. This organisation supports a model in which the *iso* gene cluster combines vertical inheritance of a functionally constrained catalytic unit with adaptive diversification of auxiliary components. By establishing a deep structural and evolutionary relationship between IsoMO and other soluble di-iron monooxygenases, this work reveals that the enzymatic machinery responsible for oxidising two of Earth’s most abundant BVOCs (methane and isoprene) shares a conserved catalytic framework that has diversified to support contrasting ecological roles. The strong conservation of IsoA further supports its use as a reliable molecular marker for isoprene degradation and for identifying active isoprene-degrading populations in environmental studies. Future work should prioritize isolation and experimental validation of predicted IsoMO complexes and structure-guided inhibitor interactions. Integrating structural insights with inhibitor-based imaging, microcosm, and mesocosm studies will improve understanding of microbial communities influence VOC fluxes under changing environmental conditions.

## Supplementary Material

fiag004_Supplemental_Files

## Data Availability

The data underlying this article (alignments and structural models) are available in ZENODO at DOI 10.5281/zenodo.18410741.
