# Supplementary material for: Structural and evolutionary insights into the isoprene monooxygenases
Source: FEMS Microbiol Ecol. 2026 Jan 22;102(3):fiag004. doi: 10.1093/femsec/fiag004 (PMC12917321; doi:10.1093/femsec/fiag004)
Supplement: fiag004_Supplemental_Files [file fiag004_supplemental_files.zip › UNTRACKED_revised_Supplementary_Fig_v2.docx]

**
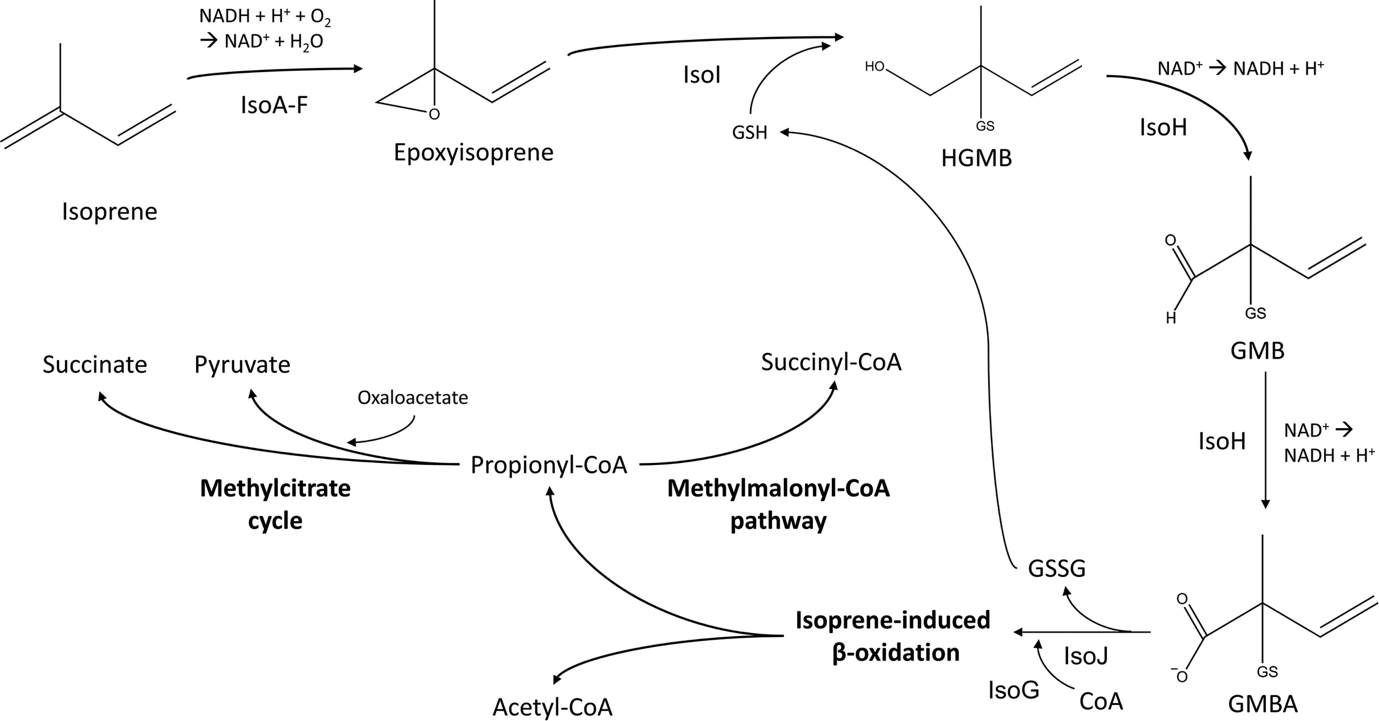
**

**Supplementary Figure S1. Isoprene metabolic pathway in the Gram-positive model bacterium *Rhodococcus* sp. AD45.** HGMB, 1-hydroxy-2-glutathionyl-2-methyl-3-butene; GMB, 2-glutathionyl-2-methyl-3-butenal; GMBA, 2-glutathionyl-2-methyl-3-butenoic acid; SG, glutathione; GSH, reduced glutathione; X-CoA, donor. The figure is modified from Rix *et al*., 2023.

**
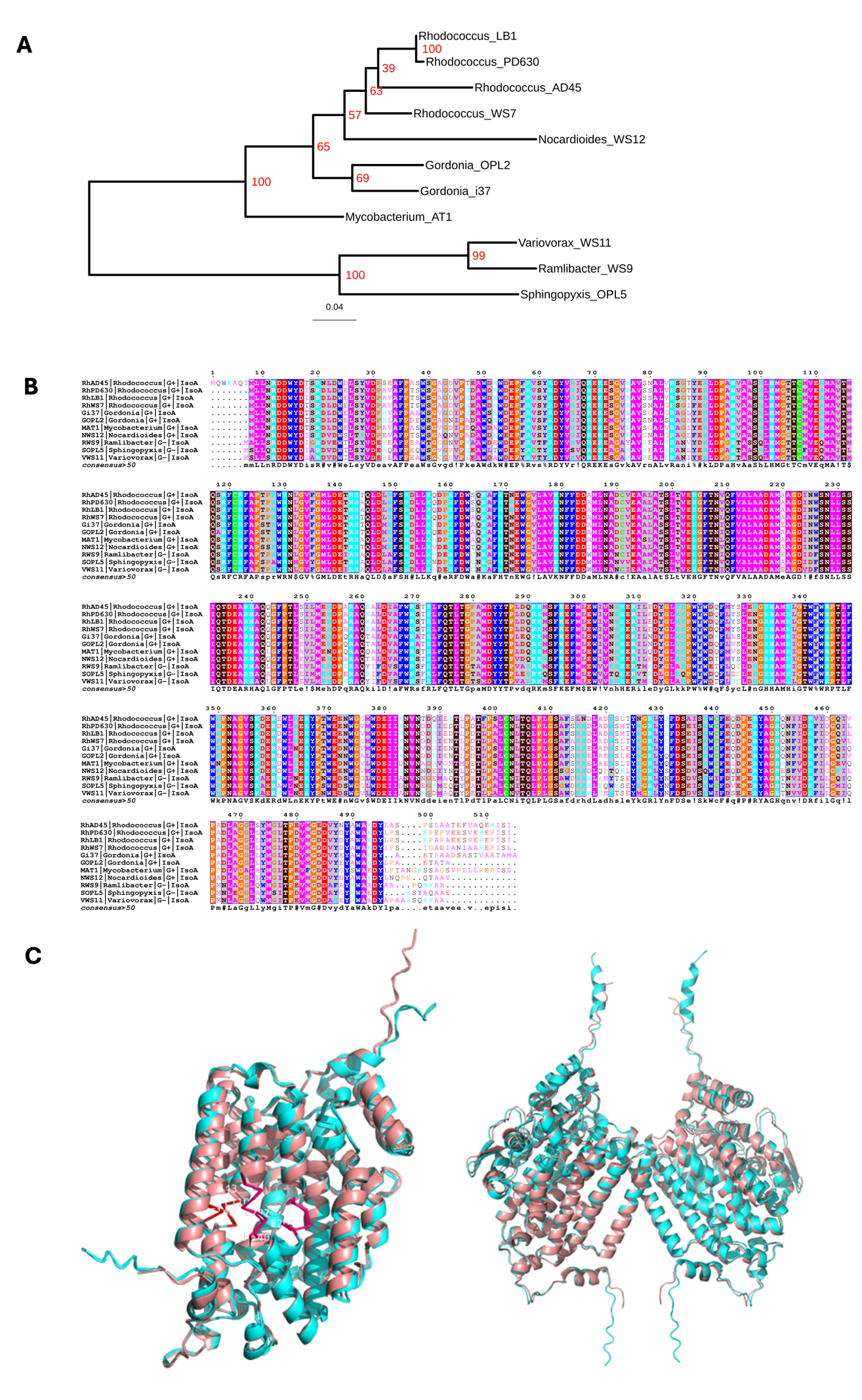
Supplementary Figure S2.** **IsoMO core α-subunit (IsoA) sequence analysis.** (A) Maximum-likelihood phylogeny of IsoA protein sequences from 11 representative isoprene-degrading bacteria. UFBoot node-support values are shown in red. The tree was inferred from full-length protein sequences (518 amino acids). Scale bar indicates substitutions per site. (B) Multiple sequence alignment of IsoA proteins visualised with ESPript 3.0, based on MAFFT-trimmed alignments (507 aligned positions after removal of gaps and poorly aligned regions). (C) Two angles of the AlphaFold2 structural model of IsoA proteins from *Rh*. AD45 (Gram-positive; cyan) and *V*. WS11 (Gram-negative; salmon). Models were predicted with AlphaFold2 via ColabFold (default parameters). Monomeric and dimeric forms are shown, with superposition highlighting conserved α-helical/β-sheet cores and clade-specific loop differences. Predicted iron-ligand residues are marked in red (*V.* WS11) and magenta (*Rh*. AD45, e.g. E110, H143, E237). All AlphaFold models were scored using per-residue pLDDT values; for IsoA, the mean pLDDT was ~91.8, indicating high model confidence, particularly around conserved di-iron coordination residues.

**
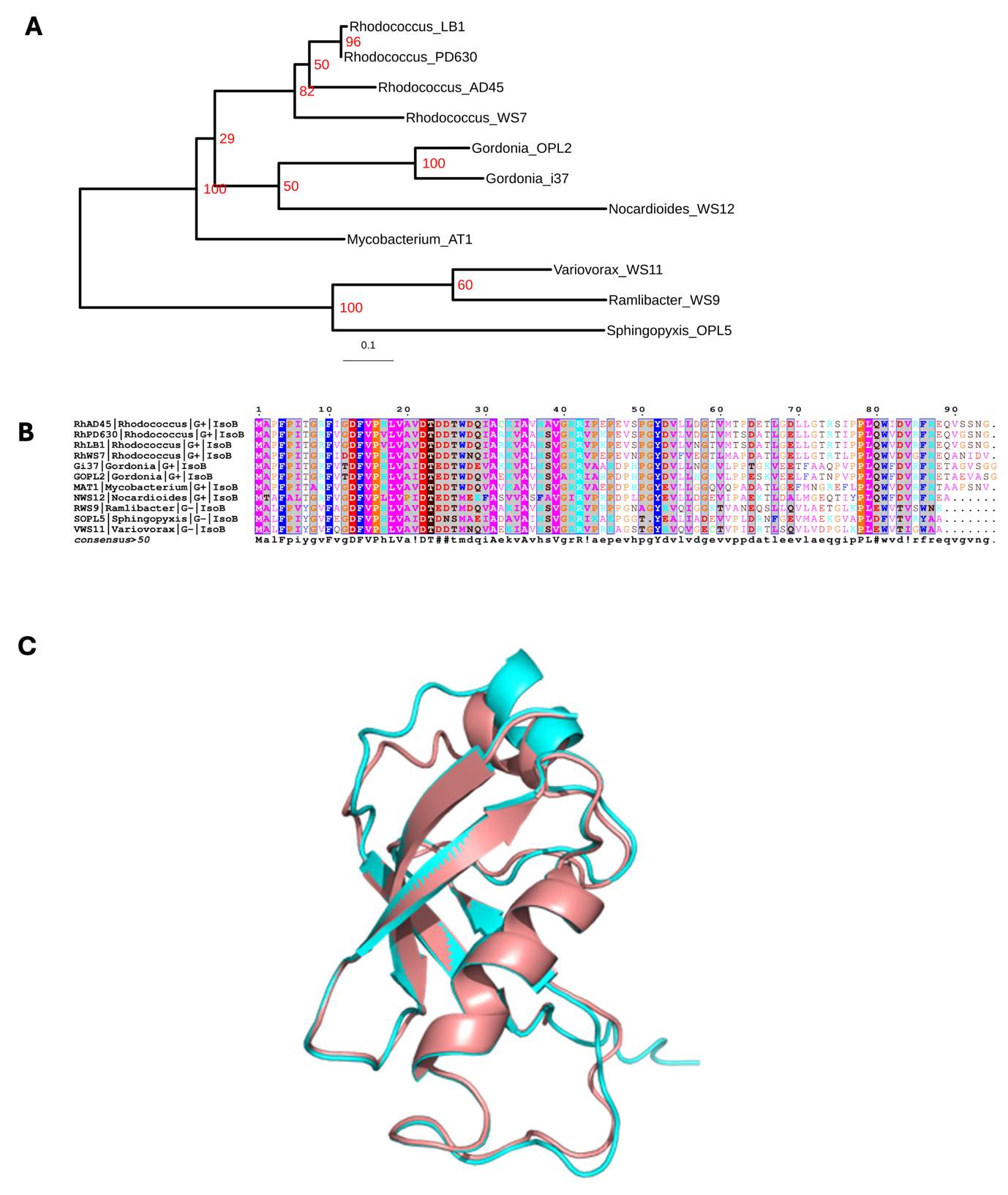
Supplementary Figure S3.** **IsoMO core γ-subunit (IsoB) sequence analysis.** (A) Maximum-likelihood phylogeny of IsoB protein sequences from 11 representative isoprene-degrading bacteria. UFBoot node-support values are shown in red. The tree was inferred from full-length protein sequences (95 amino acids). Scale bar indicates substitutions per site. (B) Multiple sequence alignment of IsoB proteins visualised with ESPript 3.0, based on MAFFT-trimmed alignments (89 aligned positions after removal of gaps and poorly aligned regions). (C) AlphaFold2 structural models of IsoB proteins from *Rh*. AD45 (Gram-positive; cyan) and *V*. WS11 (Gram-negative; salmon). Models were predicted with AlphaFold2 via ColabFold as above. Prediction confidence was high across the β1–α2 core (mean pLDDT > 90), with moderate scores in peripheral loops (pLDDT 70–85), indicating reliable modelling of the conserved structural framework.

**
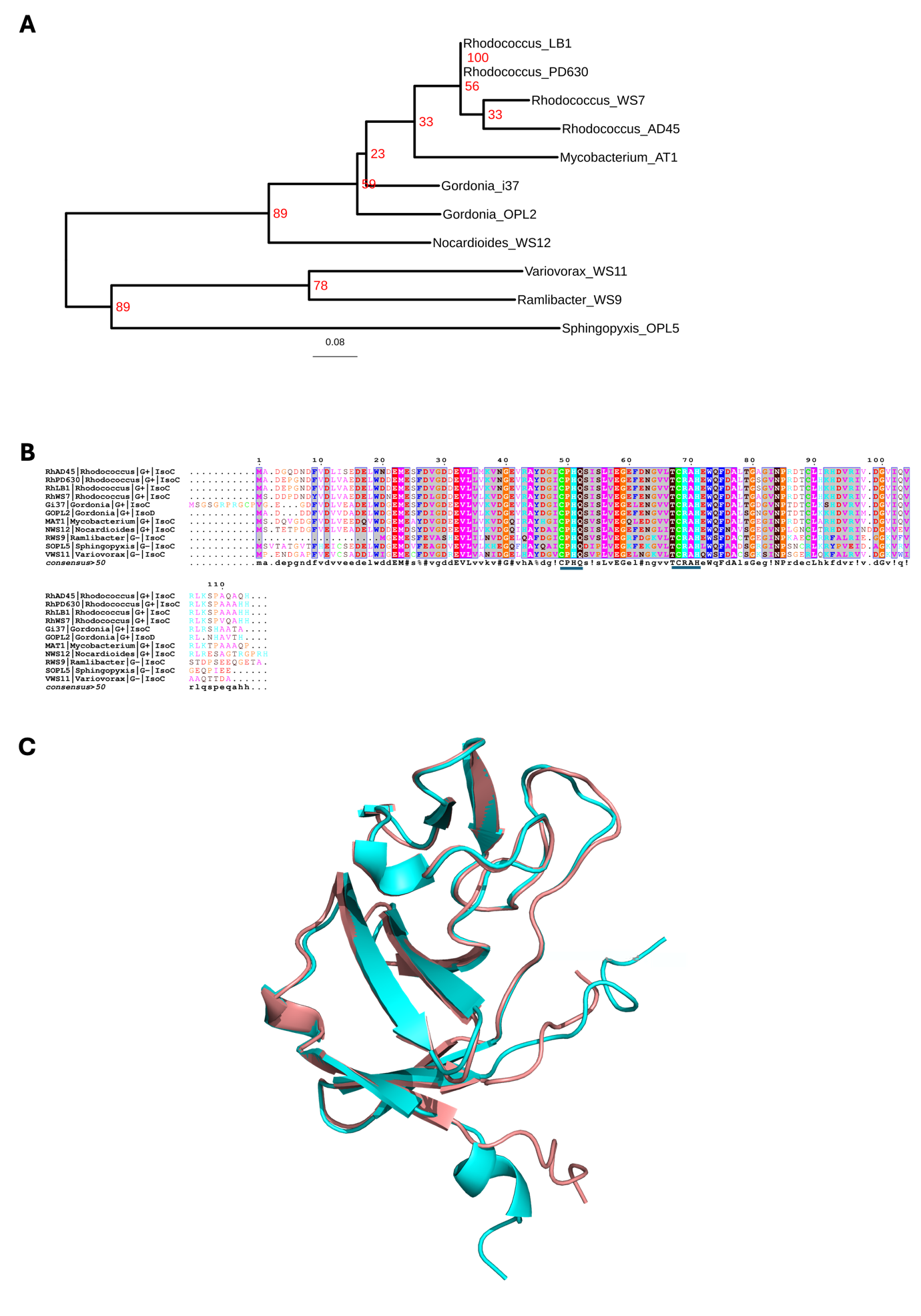
Supplementary Figure S4.** **The Rieske-type ferredoxin (IsoC) sequence analysis.** (A) Maximum-likelihood phylogeny of IsoC protein sequences from 11 representative isoprene-degrading bacteria. UFBoot node-support values are shown in red. The tree was inferred from full-length protein sequences (126 amino acids). Scale bar indicates substitutions per site. (B) Multiple sequence alignment of IsoC proteins visualised with ESPript 3.0, based on MAFFT-trimmed alignments (89 aligned positions after removal of gaps and poorly aligned regions). *Rh*. AD45 was used as reference for secondary-structure annotation. Conserved cluster coordination motifs CPHQ (C49–Q52) and TCRAH (T67–H71). (C) AlphaFold2 structural models of IsoC proteins from *Rh*. AD45 (Gram-positive; cyan) and *V*. WS11 (Gram-negative; salmon), were predicted with AlphaFold2 via ColabFold as above. Highlighted residues fall within high-confidence regions (pLDDT > 95), supporting structural conservation across Gram-positive and Gram-negative IsoC proteins.

**
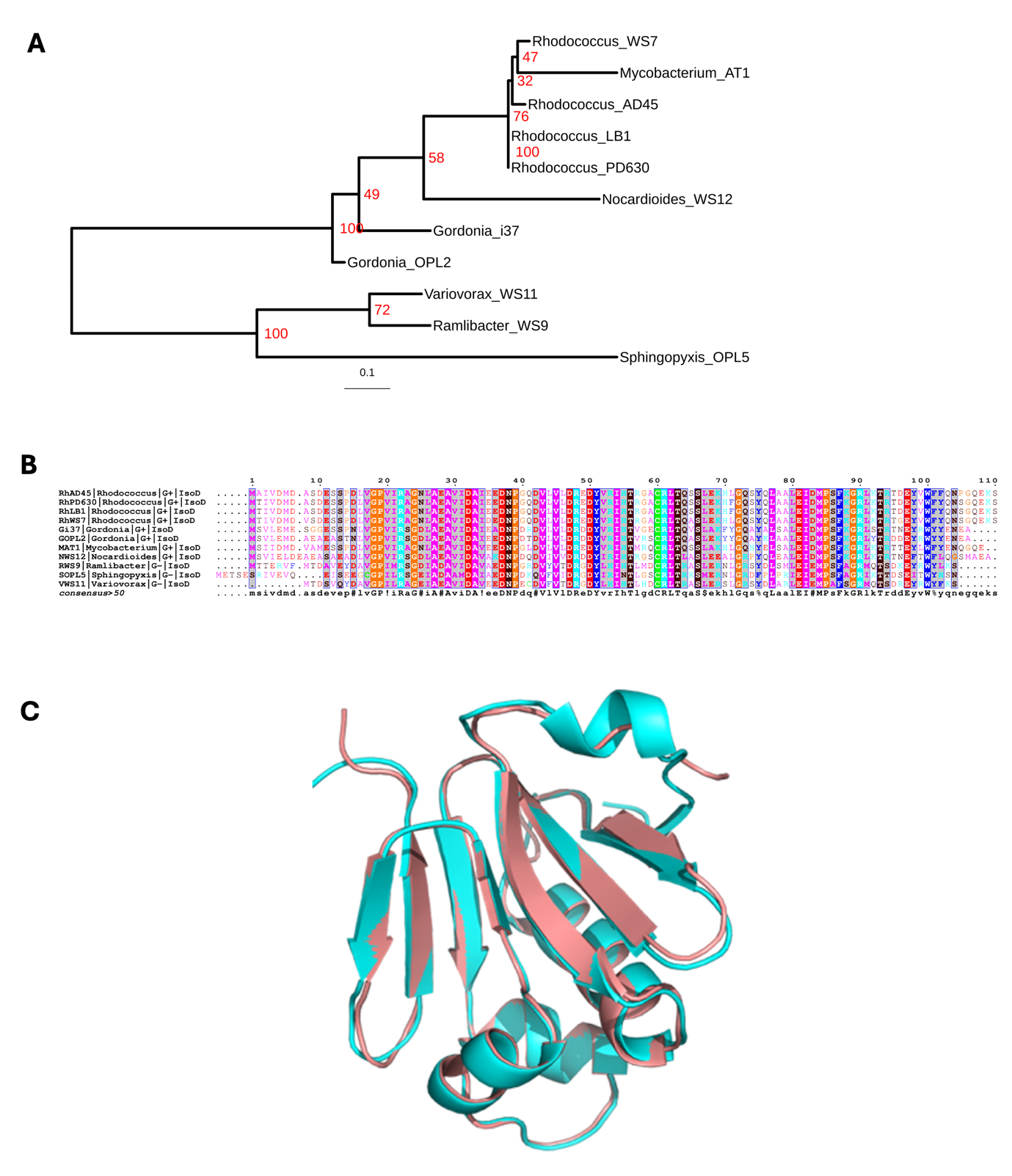
Supplementary Figure S5.** **The coupling subunit (IsoD) sequence analysis.** (A) Maximum-likelihood phylogeny of IsoD protein sequences from 11 representative isoprene-degrading bacteria. UFBoot node-support values are shown in red. The tree was inferred from full-length protein sequences (113 amino acids). Scale bar indicates substitutions per site. (B) Multiple sequence alignment of IsoD proteins visualised with ESPript 3.0, based on MAFFT-trimmed alignments (104 aligned positions after removal of gaps and poorly aligned regions). *Rh*. AD45 was used as reference for secondary-structure annotation. (C) AlphaFold2 structural models of IsoD proteins from *Rh*. AD45 (Gram-positive; cyan) and *V*. WS11 (Gram-negative; salmon), were predicted with AlphaFold2 via ColabFold as above. Key conserved cluster coordination residues (Cys37, His39, Cys56, His59 and Glu72–Glu81) fall within high-confidence regions (pLDDT > 92), supporting their structural and functional relevance.

**
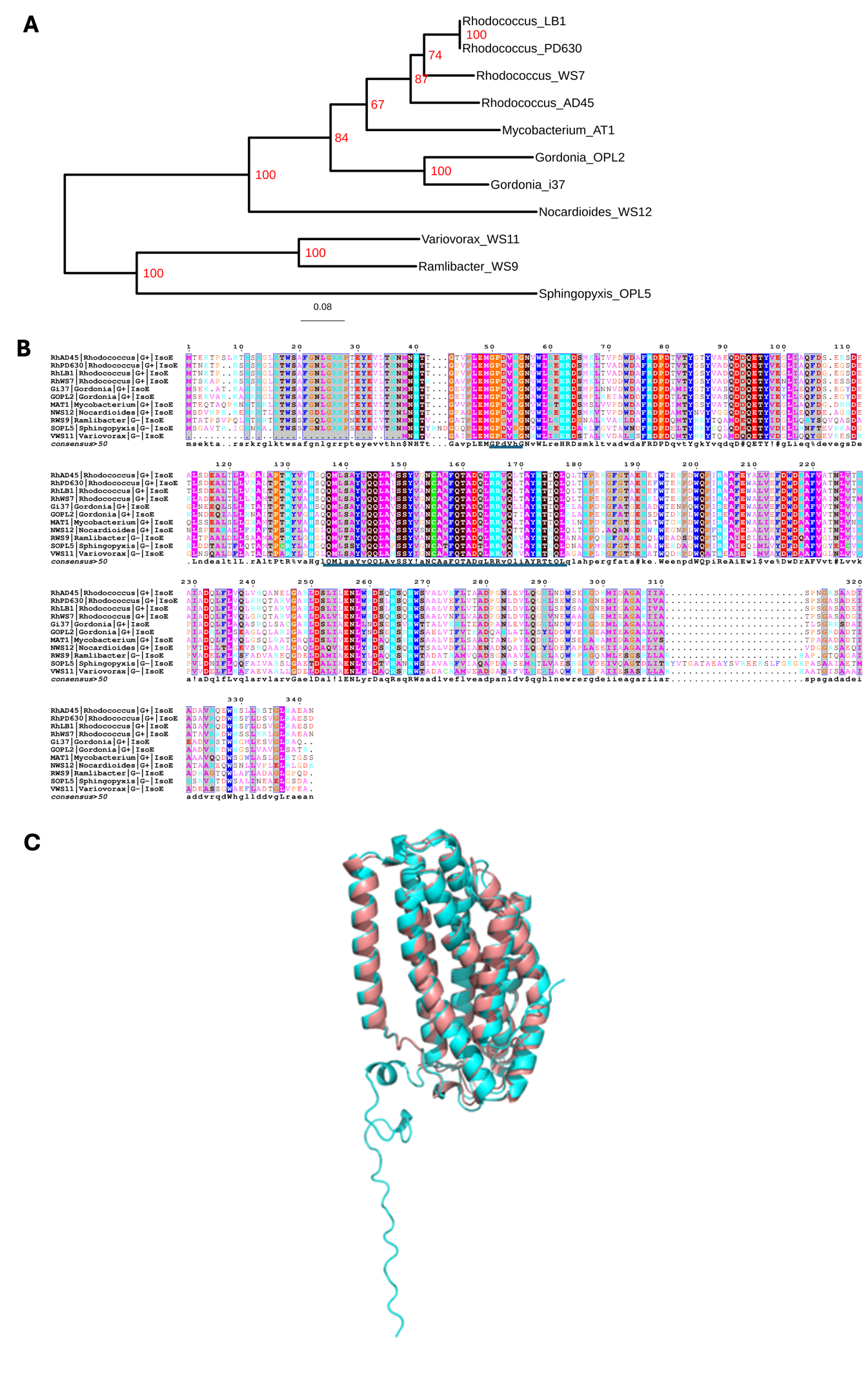
Supplementary Figure S6. The β-subunit of the IsoMO core (IsoE) sequence analysis.** (A) Maximum-likelihood phylogeny of IsoE protein sequences from 11 representative isoprene-degrading bacteria. UFBoot node-support values are shown in red. The tree was inferred from full-length sequences (372 amino acids). Scale bar indicates substitutions per site. (B) Multiple sequence alignment of IsoE proteins visualised with ESPript 3.0, based on MAFFT-trimmed alignments (339 aligned positions). *Rh*. AD45 was used as reference for secondary-structure annotation. Conserved regions include a β-strand region (residues 148–180) and short glycine-rich segment (residues 45-55). (C) AlphaFold2 models of IsoE proteins from *Rh*. AD45 and *V*. WS11 were predicted with AlphaFold2 via ColabFold as above**.
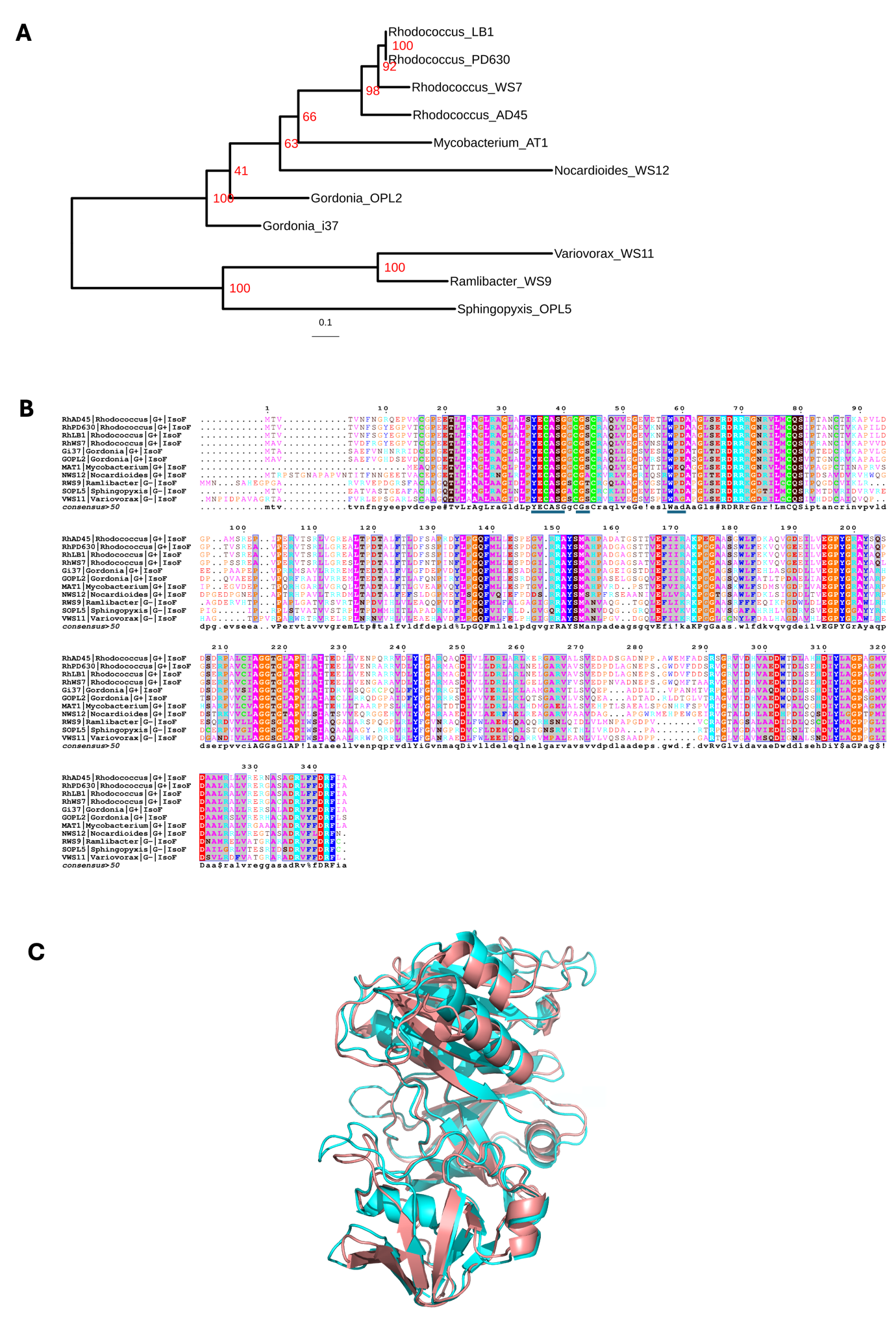
Supplementary Figure S7.** **The reductase (IsoF) sequence analysis**. A) Maximum-likelihood phylogeny of IsoF protein sequences from 11 representative isoprene-degrading bacteria. UFBoot node-support values are shown in red. The tree was inferred from full-length sequences (364 amino acids). Scale bar indicates substitutions per site. (B) Multiple sequence alignment of IsoF proteins visualised with ESPript 3.0, based on MAFFT-trimmed alignments (332 aligned positions). *Rh*. AD45 was used as reference for secondary-structure annotation. Conserved motifs within IsoF include YECASG (35–40), CGxC (42–45) and WAD (58–60). (C) AlphaFold2 models of IsoF from *Rh*. AD45 (cyan) and *V*. WS11 (salmon) were predicted with ColabFold as above. Models showed high-confidence predictions for the β-sheet/α-helix core (mean pLDDT > 80), with lower confidence in peripheral loops and variable C-terminal regions.

**
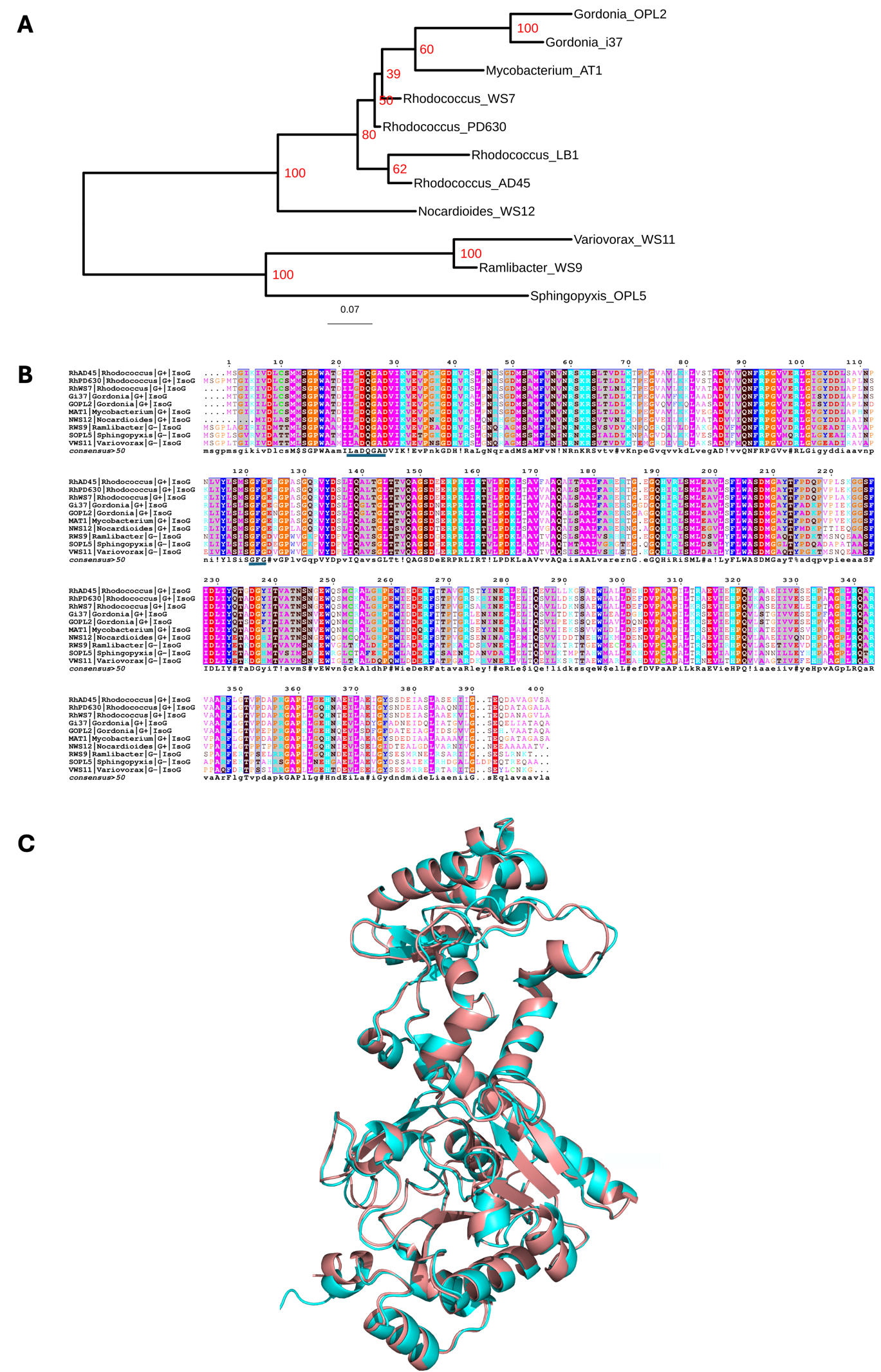
Supplementary Figure S8. The putative CoA-transferase (IsoG) sequence analysis.** (A) Maximum-likelihood phylogeny of IsoG protein sequences from 11 representative isoprene-degrading bacteria. UFBoot node-support values are shown in red. The tree was inferred from full-length sequences (406 amino acids). Scale bar indicates substitutions per site. (B) Multiple sequence alignment of IsoG proteins visualised with ESPript 3.0, based on MAFFT-trimmed alignments (398 aligned positions). *Rh*. AD45 was used as reference for secondary-structure annotation. Conserved features include the candidate residues for the catalytic glutamate (E) residues 33 and 392, GxG-like motifs (G36, G101 and G121). (C) AlphaFold2 structural models of IsoG proteins from *Rh*. AD45 and *V*. WS11, were predicted with AlphaFold2 via ColabFold as above, showed high model confidence (pLDDT > 95) across catalytic regions.

**
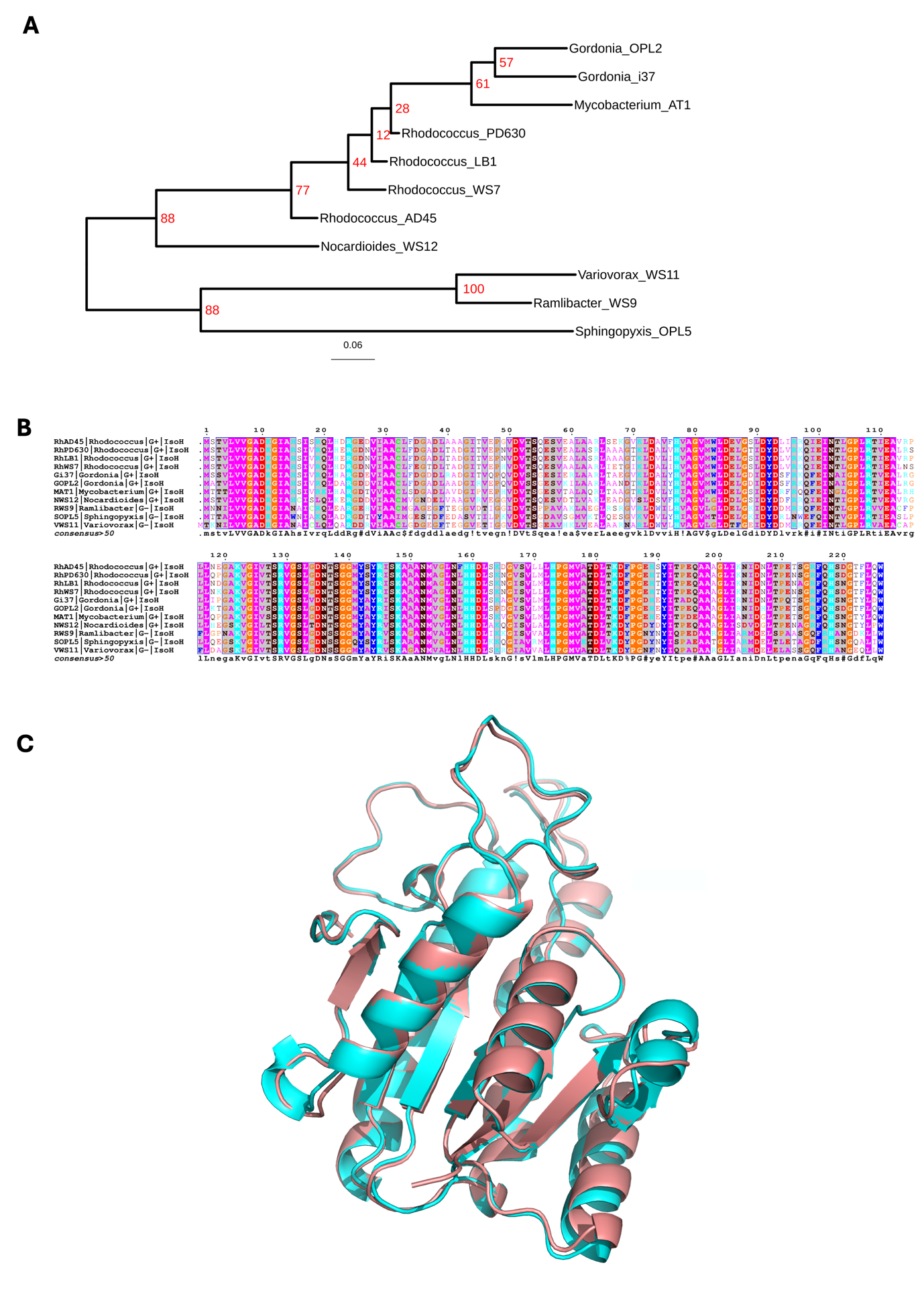
Supplementary Figure S9. NAD⁺-dependent dehydrogenase subunit (IsoH) sequence analysis.** (A) Maximum-likelihood phylogeny of IsoH protein sequences from 11 representative isoprene-degrading bacteria. UFBoot node-support values are shown in red. The tree was inferred from full-length sequences (227 amino acids). Scale bar indicates substitutions per site. (B) Multiple sequence alignment of IsoH proteins visualised with ESPript 3.0, based on MAFFT-trimmed alignments (226 aligned positions). *Rh*. AD45 was used as reference for secondary-structure annotation. (C) AlphaFold2 structural models of IsoH proteins from *Rh*. AD45 and *Variovorax* sp. WS11 were predicted with AlphaFold2 via ColabFold as above.

**
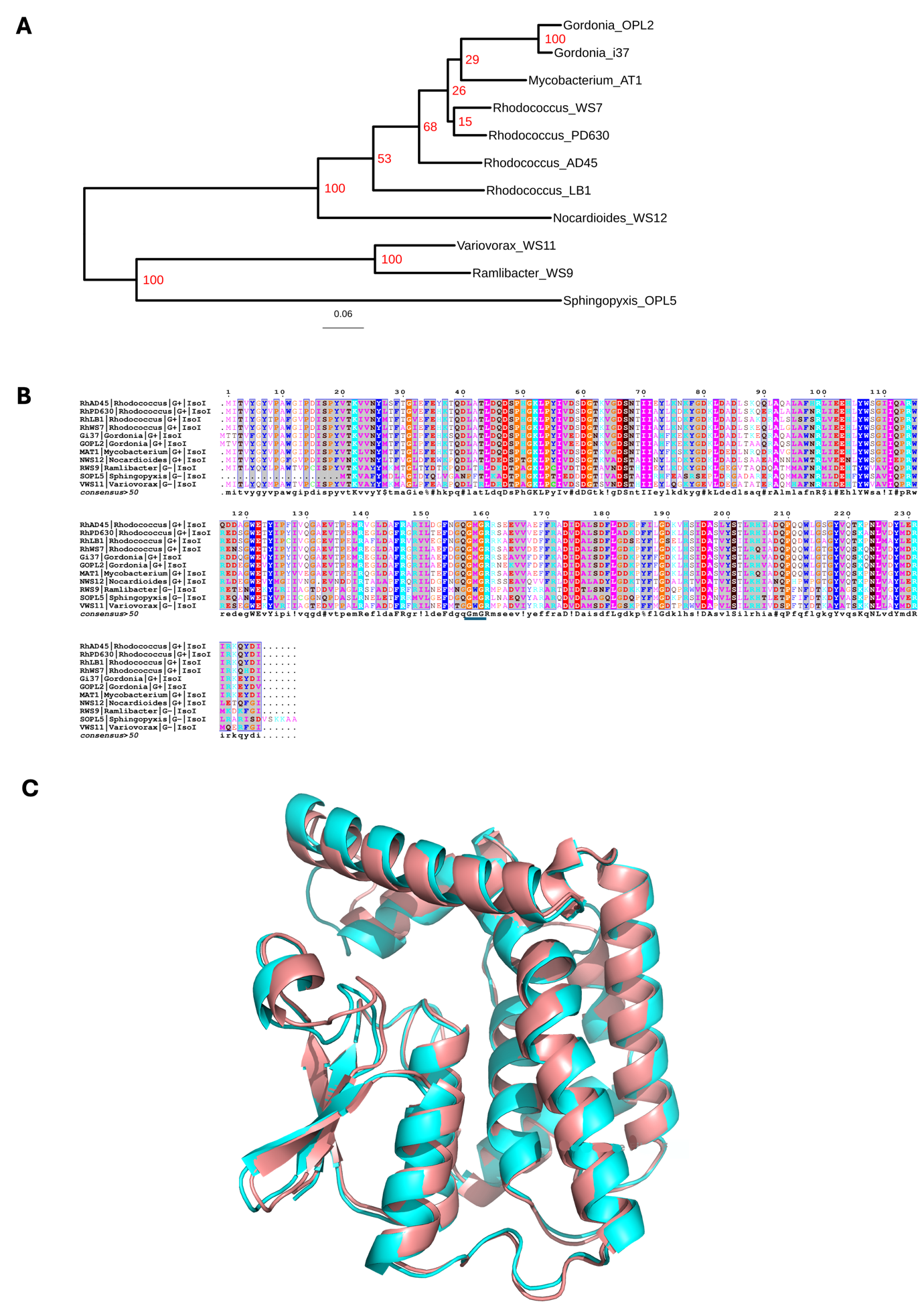
Supplementary Figure S10.** **Glutathione S-transferase -like subunit (IsoI) sequence analysis.** (A) Maximum-likelihood phylogeny of IsoI protein sequences from 11 representative isoprene-degrading bacteria. UFBoot node-support values are shown in red. The tree was inferred from full-length sequences (245 amino acids). Scale bar indicates substitutions per site. (B) Multiple sequence alignment of IsoI proteins visualised with ESPript 3.0, based on MAFFT-trimmed alignments (238 aligned positions). *Rh*. AD45 was used as reference for secondary-structure annotation. A conserved GXG loop (G157–G159) may contribute to glutathione binding. (C) AlphaFold2 structural models of IsoI proteins from *Rh*. AD45 (cyan) and *V*. WS11 (salmon) were predicted with AlphaFold2 via ColabFold as above. Predicted structures achieved high confidence (pLDDT > 90 across 92 % of residues).

**
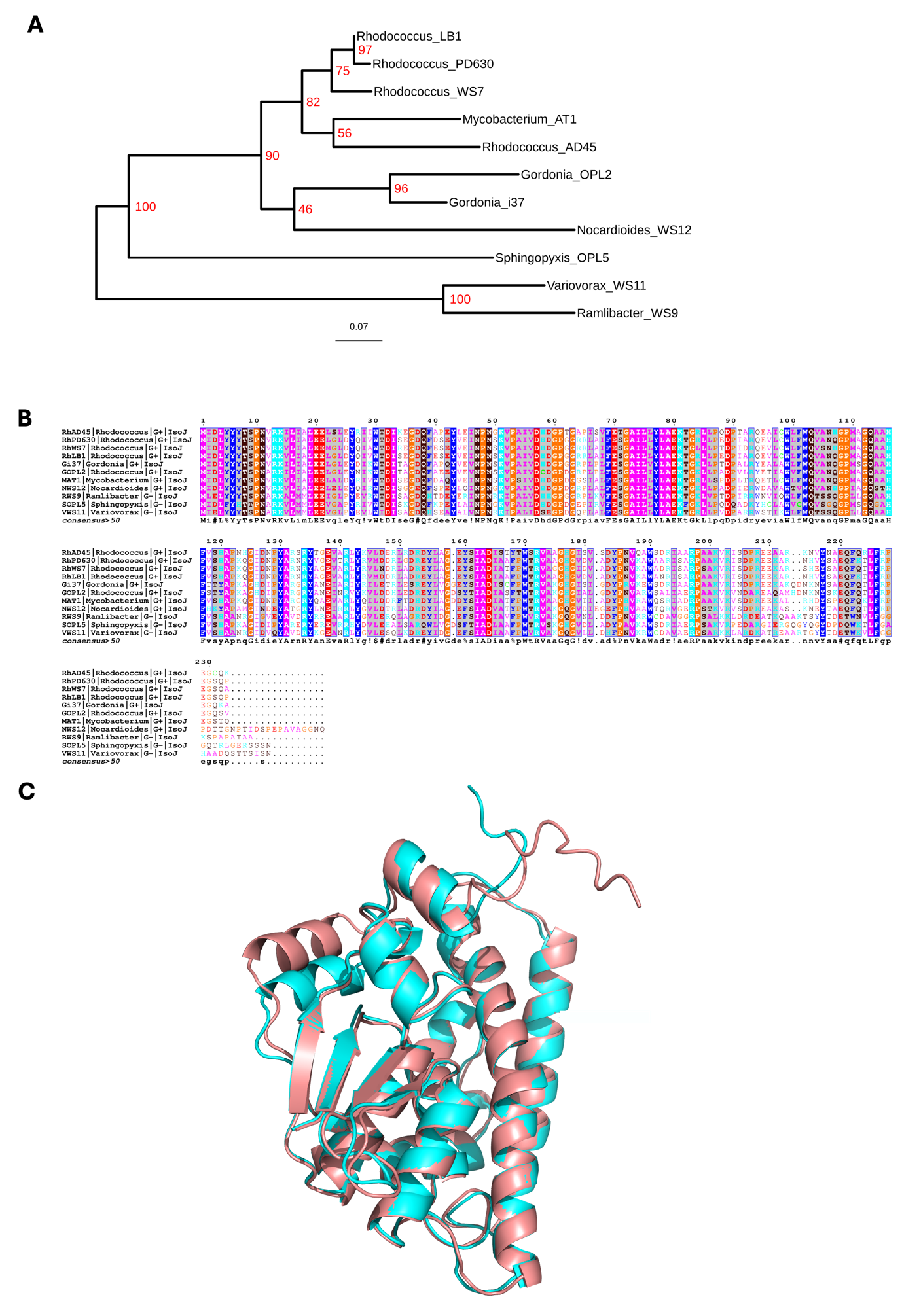
Supplementary Figure S11. Glutathione S-transferase-like subunit (IsoJ) sequence analysis.** (A) Maximum-likelihood phylogeny of IsoJ protein sequences from 11 representative isoprene-degrading bacteria. UFBoot node-support values are shown in red. The tree was inferred from full-length sequences (255 amino acids). Scale bar indicates substitutions per site. (B) Multiple sequence alignment of IsoJ proteins visualised with ESPript 3.0, based on MAFFT-trimmed alignments (237 aligned positions). *Rh*. AD45 was used as reference for secondary-structure annotation. (C) AlphaFold2 models of IsoJ proteins from *Rh*. AD45 (cyan) and *V*. WS11 (salmon) were predicted with AlphaFold2 via ColabFold as above and mapped to *Rh.* AD45 numbering. Consensus sequence is included. Predicted structures had mean pLDDT scores of 91.5 (*Rh.* AD45) and 89.7 (*V.* WS11).

**
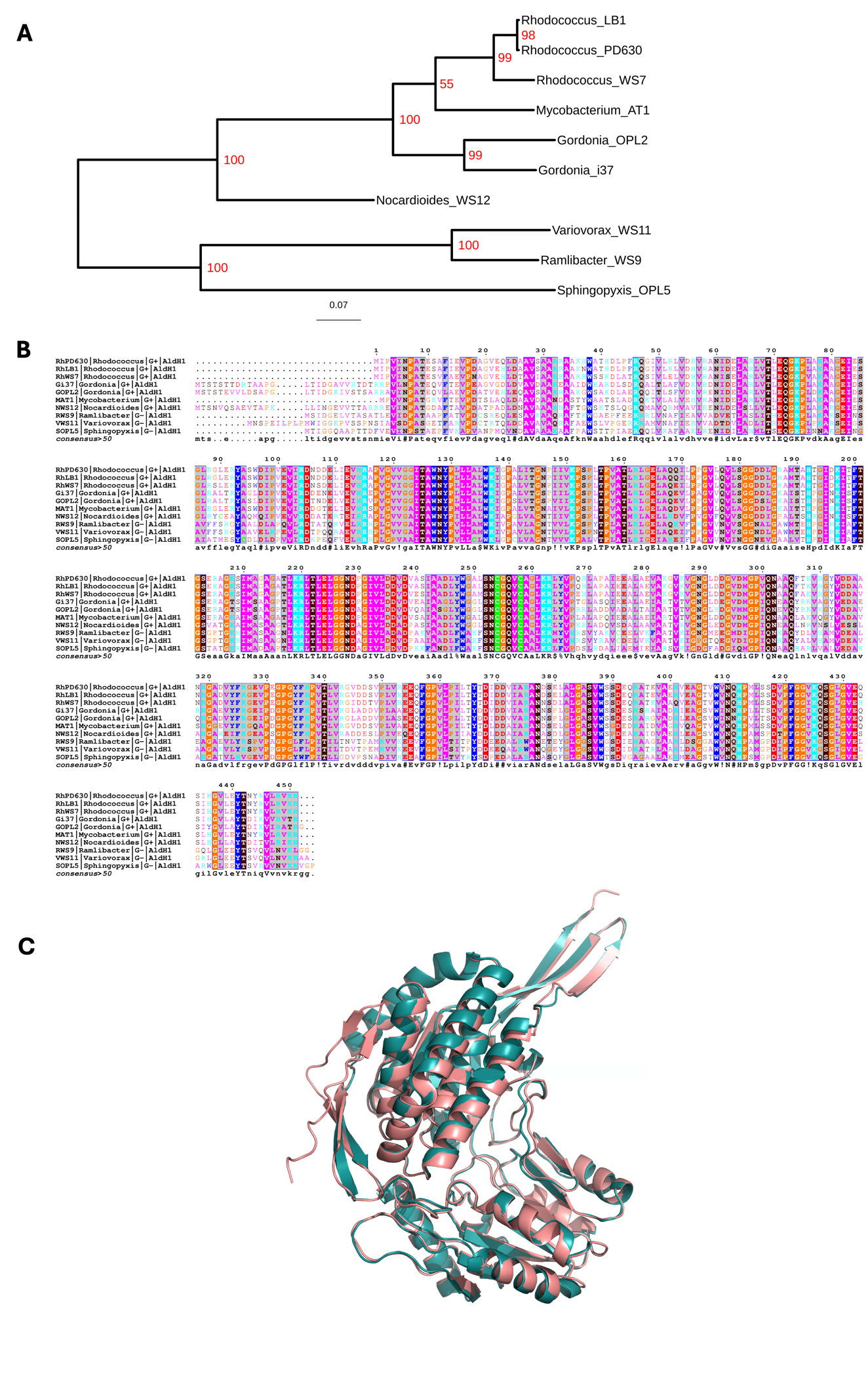
Supplementary Figure S12.** **Aldehyde dehydrogenase (AldH1) sequence analysis**. (A) Maximum-likelihood phylogeny of AldH1 protein sequences from 10 isoprene-degrading bacteria. UFBoot node-support values are shown in red. The tree was inferred from full-length sequences (480 amino acids). Scale bar indicates substitutions per site. (B) Multiple sequence alignment of trimmed AldH1 sequences visualised with ESPript 3.0 (474 aligned positions), mapped to *Rh. opacus* PD630 numbering. Consensus sequence is included. (C) AlphaFold2 models of AldH1 proteins showed high predicted confidence, with mean pLDDT scores of 95.7 (*V.* WS11) and 96.2 (*Rh. opacus* PD630).

**
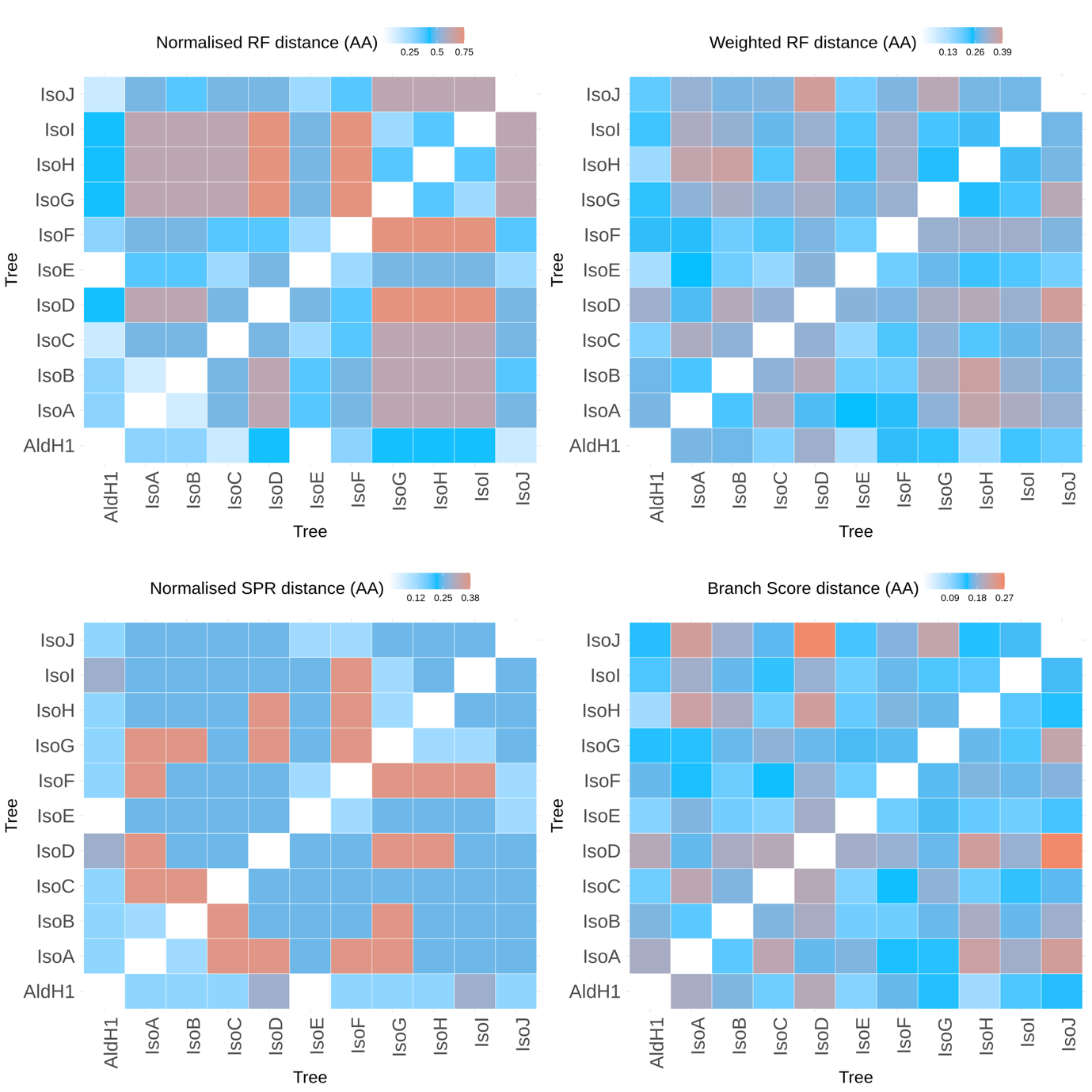
Supplementary Figure S13. Pairwise tree distances for protein phylogenies of the *iso* gene cluster.** Heatmaps show all-versus-all distances between maximum-likelihood trees of 11 *iso* genes using four metrics: (top left) Normalised Robinson–Foulds (RF) distance, (top right) Weighted RF distance, (bottom left) Normalised subtree-prune-and-regraft (SPR) distance and (bottom right) Branch-score distance. Right-hand panels incorporate branch-length information, while left-hand panels show only topological differences. Light-blue cells indicate greater similarity between trees. Overall, low distance values indicate congruent phylogenies: oxygenase core genes (*isoA–C*) showed high congruence, detoxification genes (*isoG–J*) were more divergent and AldH1 and electron-transfer genes (*isoD–F*) displayed intermediate patterns.

**
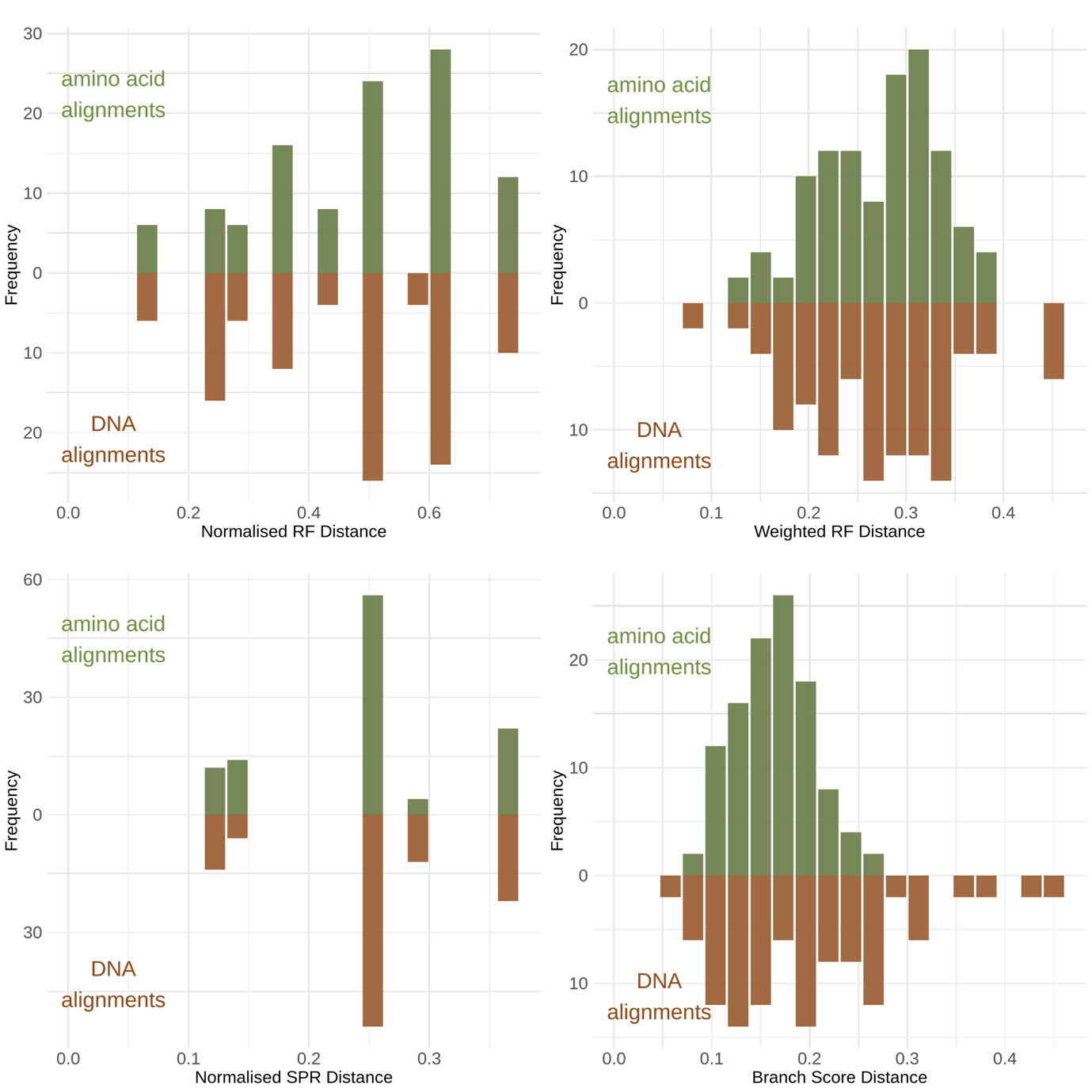
Supplementary Figure S14. Distribution of pairwise tree distances, for both nucleotide and amino acid alignments** Histograms show pairwise distances between *iso* gene phylogenies using four metrics: (top left) Normalised Robinson–Foulds (RF), (top right) Weighted RF, (bottom left) Normalised SPR and (bottom right) Branch-score distance. Right-hand panels incorporate branch lengths, while left-hand panels show topological differences only. Green distributions correspond to amino acid trees, and brown to nucleotide trees. Both data types yield similar distributions, though DNA trees exhibit slightly higher overall distances and spread when branch lengths are included. Oxygenase core genes (*isoA–C*) show low pairwise distances, while detoxification genes (*isoG–J*) are more divergent. AldH1 exhibits intermediate distances, reflecting partial decoupling from the core *iso* module.

**
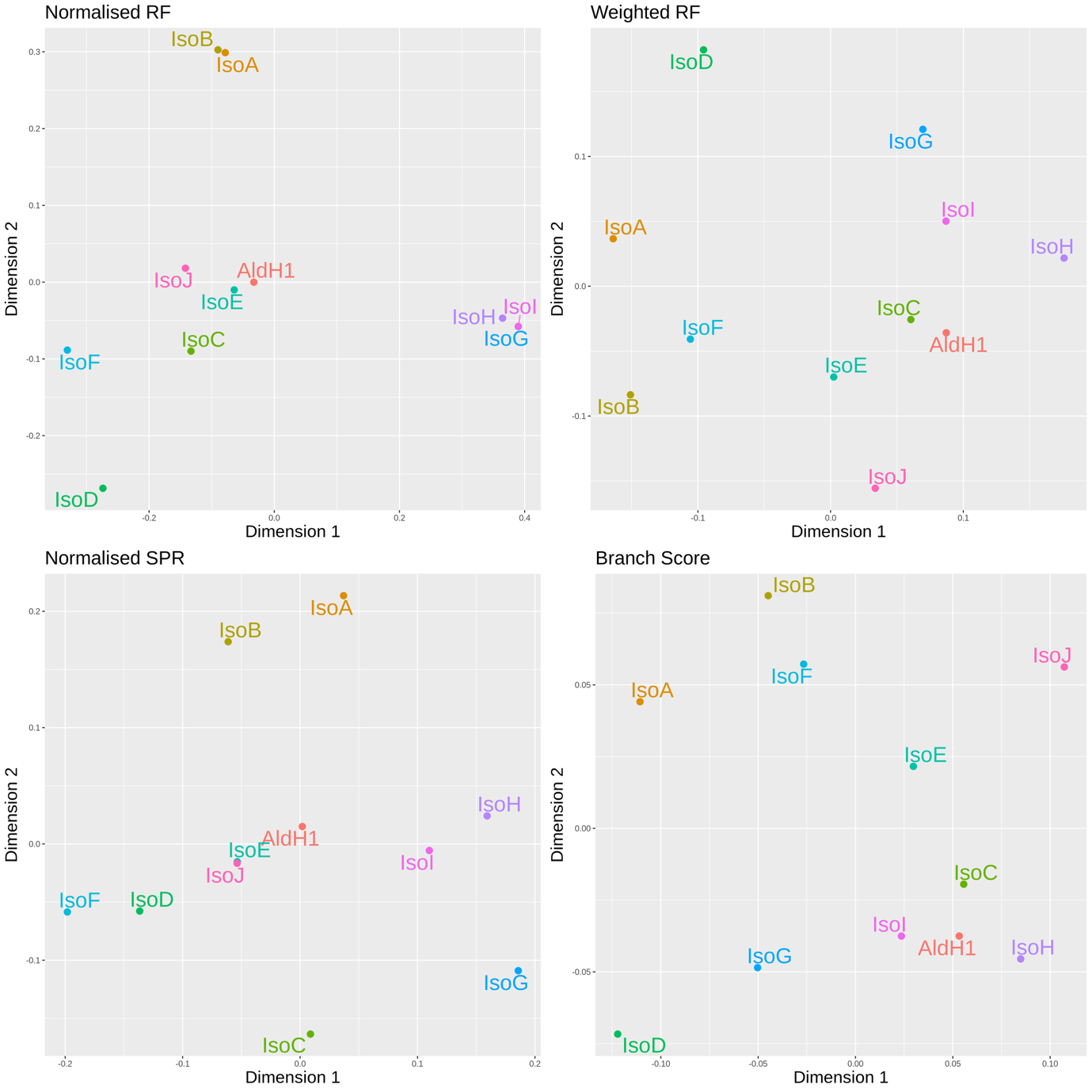
Supplementary Figure S15. Multidimensional scaling (MDS) of tree distances (amino acid alignments).** MDS plots display two-dimensional representations of pairwise distances between all protein-coding *iso* gene trees. Distances were calculated using four metrics: (top left) Normalised RF, (top right) Weighted RF, (bottom left) Normalised SPR and (bottom right) Branch-score distance. Right-hand panels include branch-length information, while left-hand panels reflect topological relationships only.

**
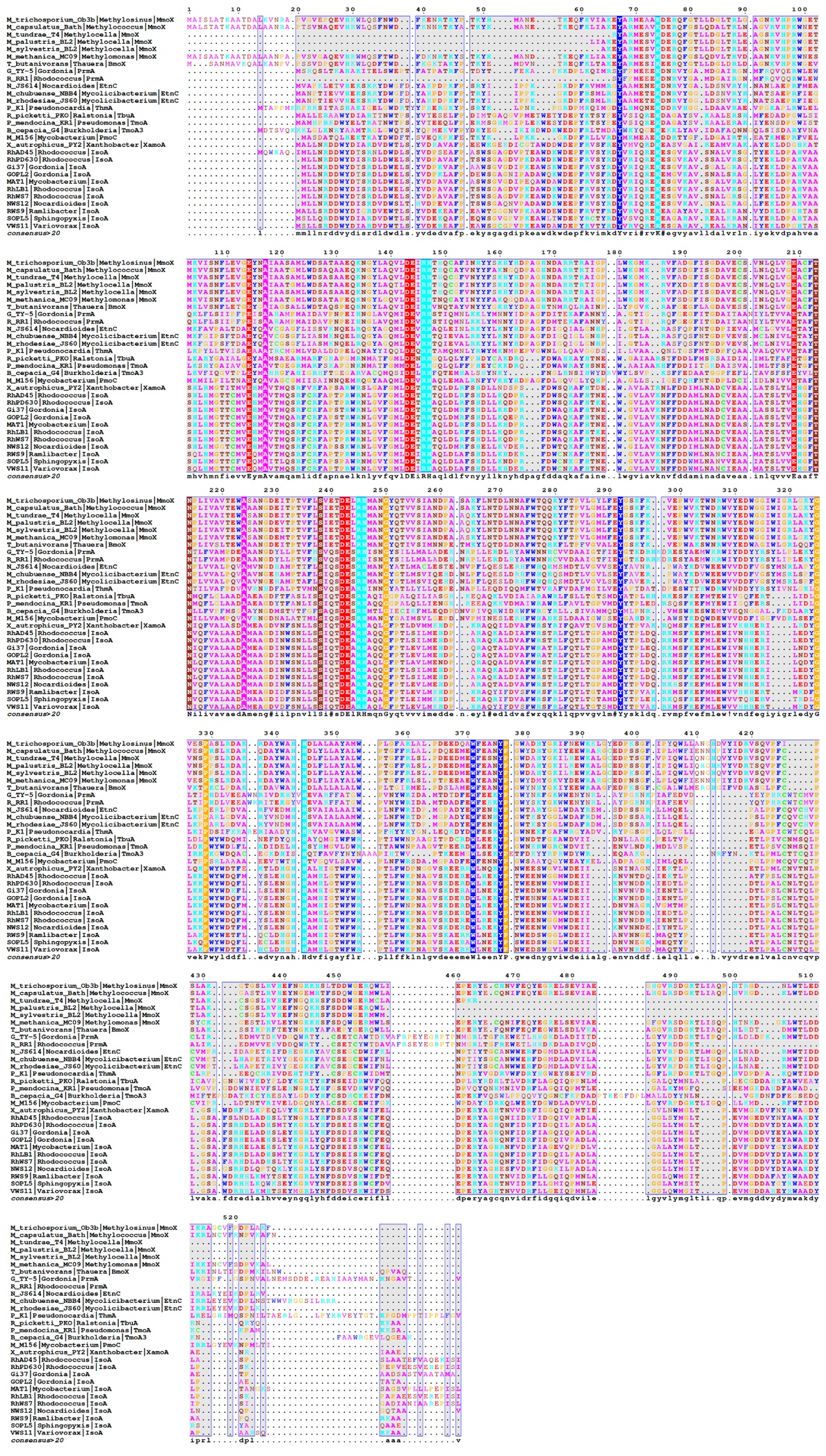
**

**Supplementary Figure S16.** Multiple sequence alignment of representative soluble di-iron monooxygenase (SDIMO) α-subunit (A-component) proteins. The alignment includes IsoA sequences from all confirmed isoprene-degrading strains in this study, together with selected representatives of other SDIMO lineages (MmoX, PrmA, BmoX, TmoA, TbuA, ThmA and XamoA). Alignments were generated using MAFFT (L-INS-i) and visualised with ESPript 3.0. Residue numbering corresponds to *Methylosinus trichosporium* Ob3b (MmoX) for consistency. Residues are coloured by physicochemical properties, and a consensus sequence is shown beneath the alignment.

**Supplementary Figure S17.** Multiple sequence alignment of five complete MmoX proteins. Sequences include the five MmoX representatives used in this study. The alignment was generated using MAFFT (L-INS-i) and visualised with ESPript 3.0, with secondary-structure elements derived from the RCSB PDB entry 1MTY (*Methylococcus capsulatus* Bath) indicated. Residue numbering corresponds to *Methylosinus trichosporium* Ob3b (MmoX) for consistency. A consensus sequence is shown below the alignment.

**
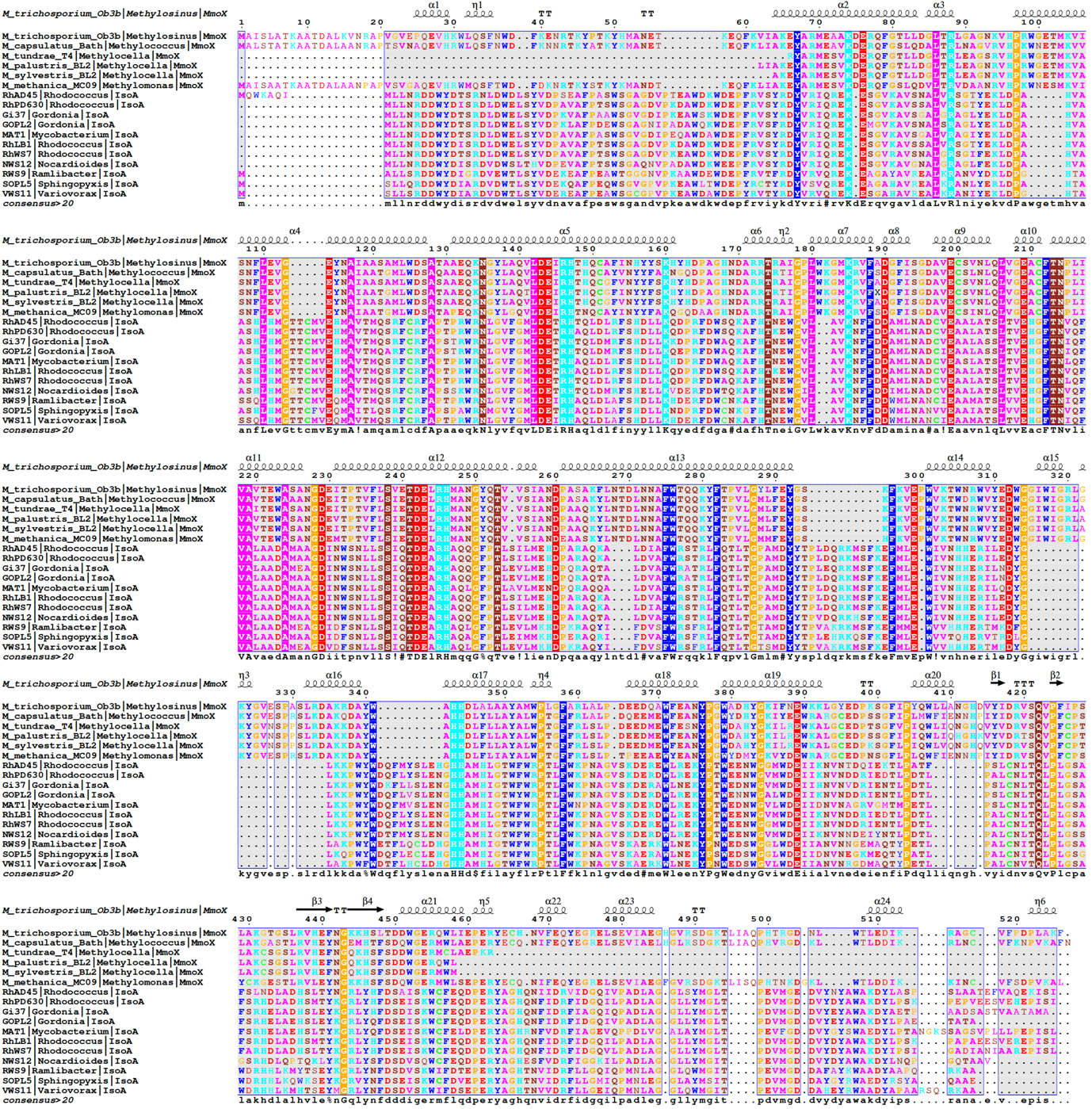
**

**Supplementary Figure S18.** Multiple sequence alignment of IsoA and MmoX, α-subunits of the IsoMO core and sMMO, respectively. Alignment of 11 complete IsoA sequences and six MmoX sequences was generated using MAFFT (L-INS-i) and visualised with ESPript 3.0, with residues coloured by physicochemical properties. The structure of MmoX from *Methylococcus capsulatus* (Bath; PDB 1MTY) was used as reference for secondary-structure annotation. Residue numbering corresponds to *Methylosinus trichosporium* Ob3b (MmoX) for consistency. A consensus sequence is shown below the alignment.

**
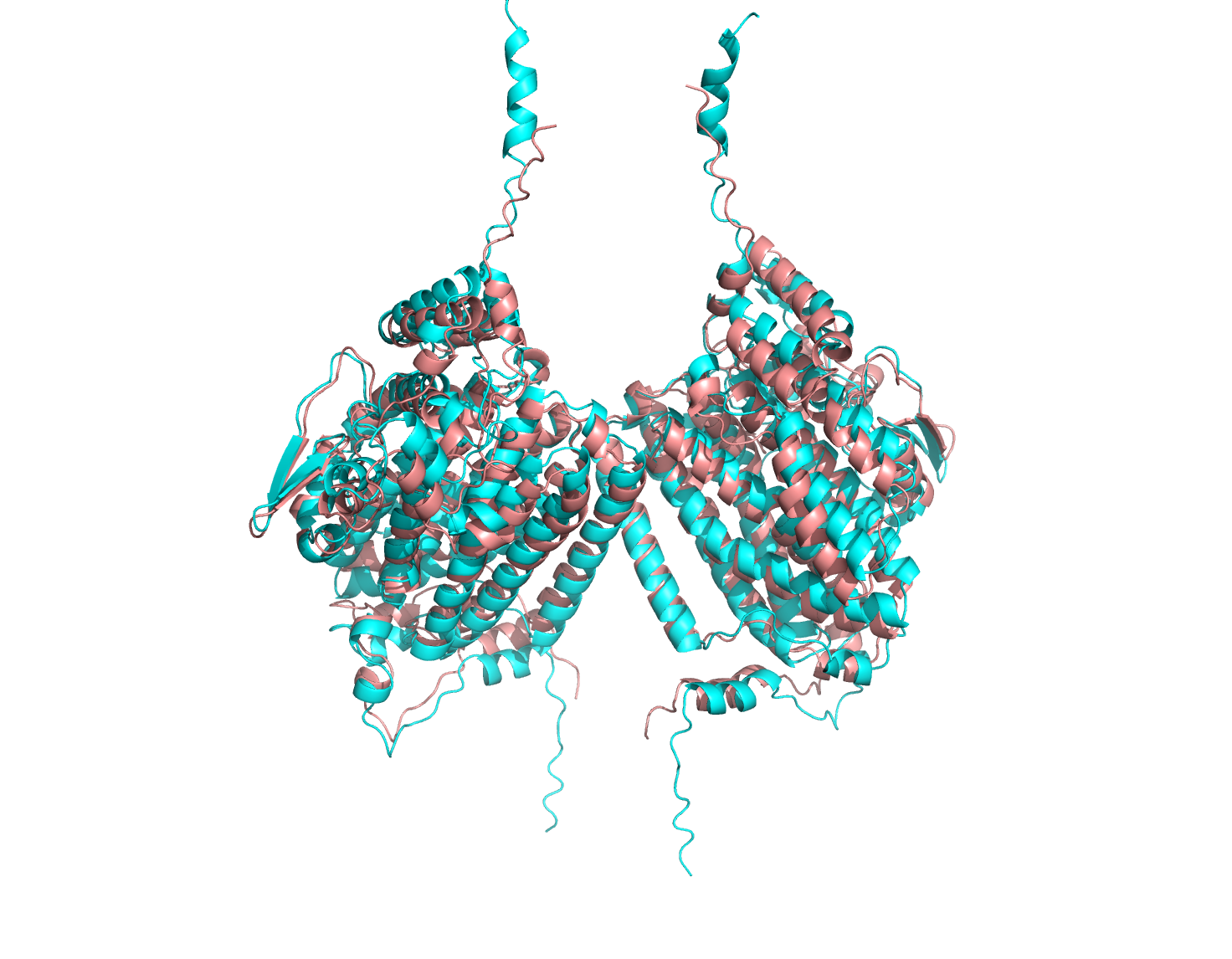
**

**Supplementary Figure S19.** Comparison of IsoA α-subunit dimers from Gram-positive and Gram-negative isoprene degraders. AlphaFold2-predicted dimeric models of the IsoA α-subunit from *Rh*. AD45 (Gram-positive; cyan) and *V*. WS11 (Gram-negative; salmon) were superimposed and visualised in PyMOL. Both models display conserved dimeric architecture and catalytic core, with subtle clade-specific variations in peripheral helices and loop regions that may reflect lineage-specific adaptations of IsoMO to distinct cellular environments. Mean model confidence was pLDDT = 90.8.


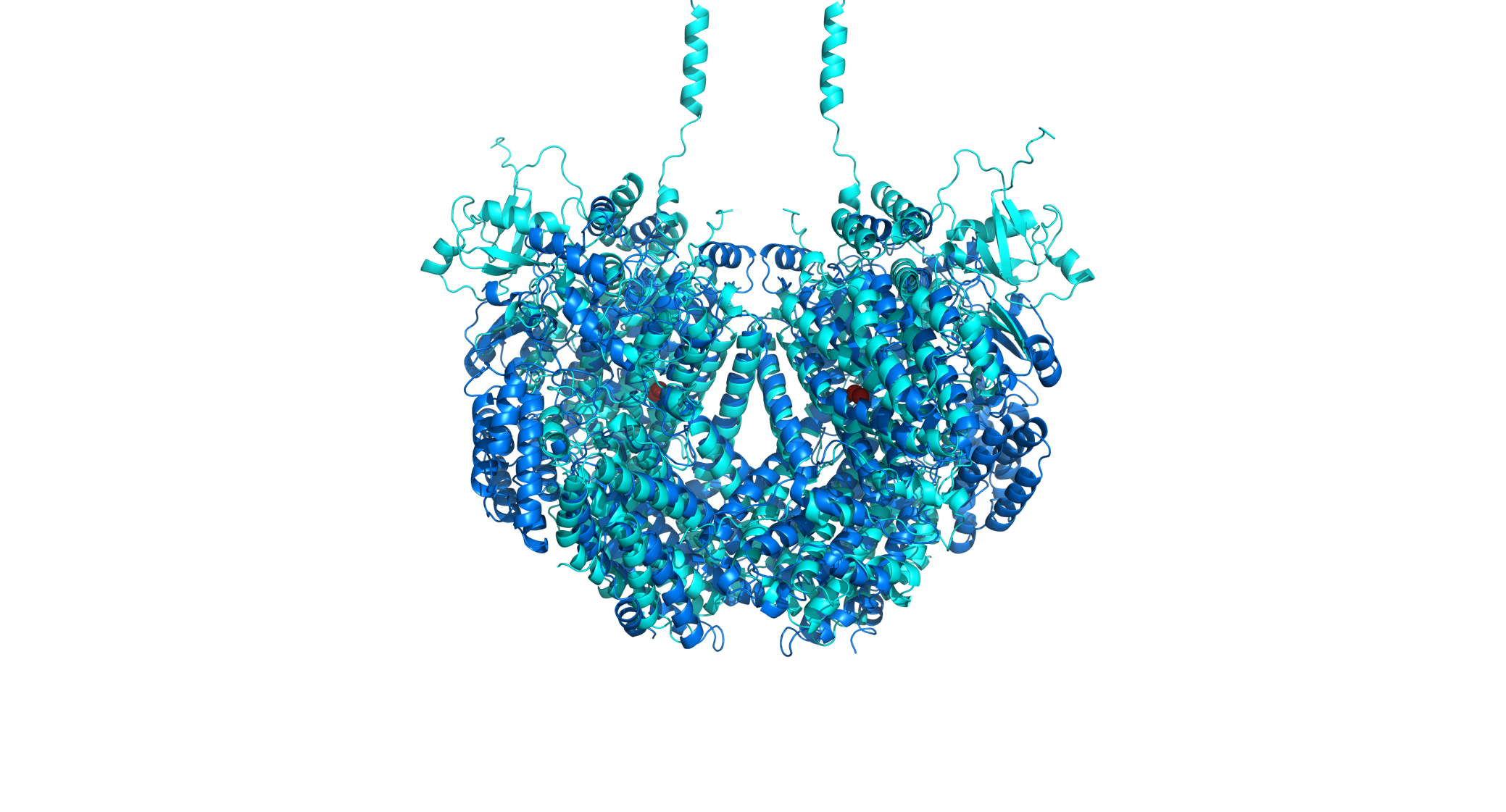

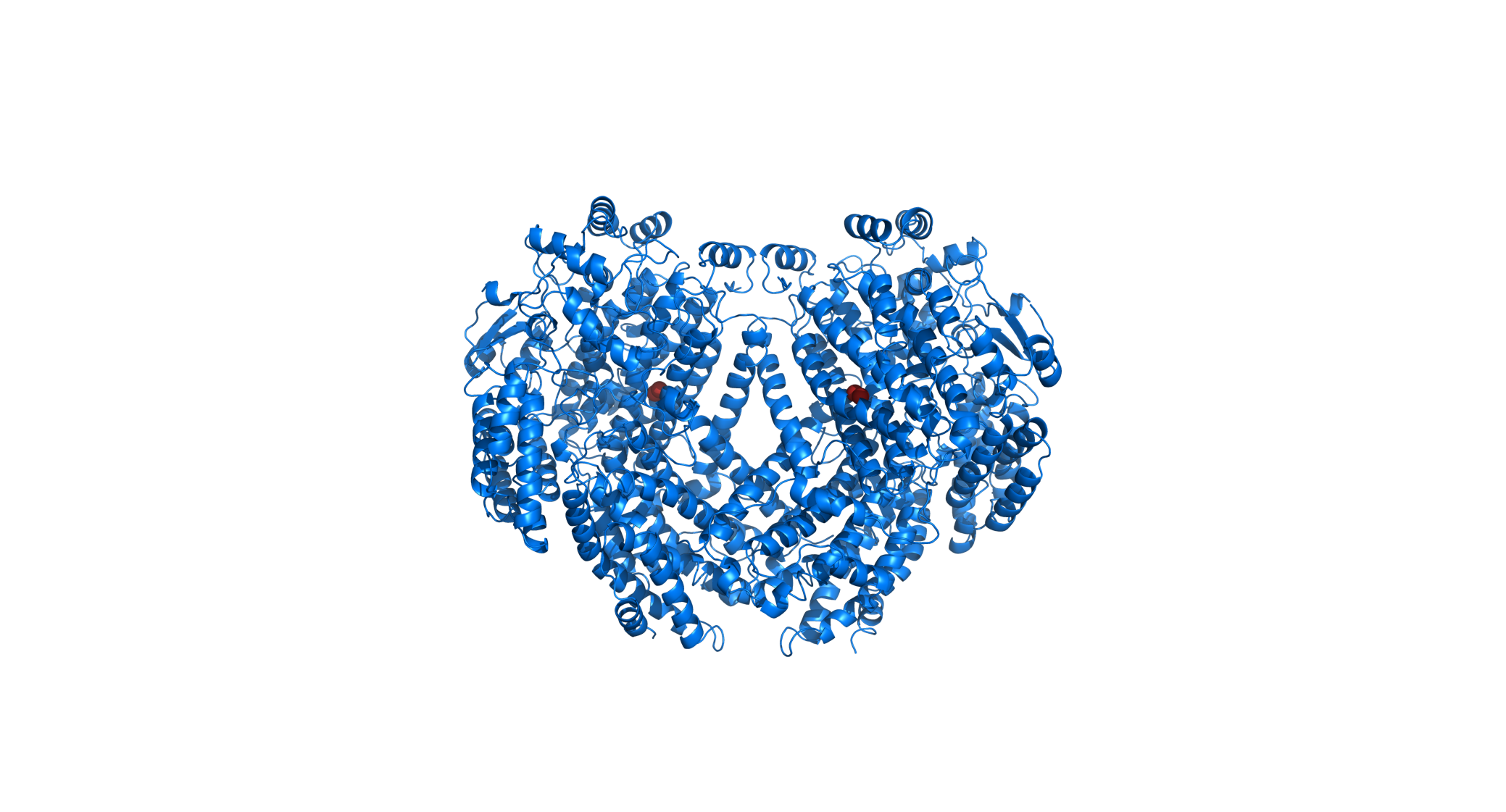


**Supplementary Figure S20.** (A) Structural comparison between the IsoMO core and sMMO hydroxylase (α₂β₂γ₂). Superposition of the predicted *Rh*. AD45 IsoMO core (cyan) and the crystal structure of the soluble methane monooxygenase (sMMO) hydroxylase from *Methylococcus* *capsulatus* Bath (PDB: 1MTY; blue). Both complexes share the characteristic α₂β₂ di-iron monooxygenase architecture, with closely aligned α-helical bundles and conserved di-iron centres (orange). The strong structural correspondence supports a close evolutionary and mechanistic relationship between IsoMO core and other SDIMO. (B) sMMO hydroxylase (1MTY), including di-iron atoms in red.
