# Supplementary material for: Structural and evolutionary insights into the isoprene monooxygenases
Source: FEMS Microbiol Ecol. 2026 Jan 22;102(3):fiag004. doi: 10.1093/femsec/fiag004 (PMC12917321; doi:10.1093/femsec/fiag004)
Supplement: fiag004_Supplemental_Files [file fiag004_supplemental_files.zip › UNTRACKED_revised_Supplementary_Notes_v3.docx]

**Phylogenetic analysis and alignment of proteins encoded by the *iso* cluster**

Protein analyses are summarized below, with phylogenetic trees (A), multiple sequence alignment (B), and AlphaFold models (C) shown in Figures S2–S12. Below we summarise the main phylogenetic and sequence alignment features for each protein, with G⁺/G⁻ variation profiles highlighted and conserved motifs referenced to *Rh.* AD45 numbering from the multiple sequence alignment.

**IsoA** (α-subunit of the IsoMO; average length 504 amino acids, Figure S2; n=11) was the most conserved protein within the *iso* cluster, with pairwise amino acid identities of 82–100% among Gram-positive sequences, 81–100% among Gram-negative sequences, and 71–75% between Gram groups. Maximum likelihood phylogenies (Fig. S2A, 507 aligned positions) consistently resolved Gram-positive and Gram-negative IsoA sequences as distinct, well-supported clades. Indicating long-term lineage-specific divergence while having the lowest phylogenetic diversity (sum of branch lengths on the tree = 1.47). Multiple sequence alignment (Fig. S2B, full annotation Figure 3 and Table 2) revealed conservation of hallmark SDIMO motifs based on Leahy *et al*., 2003 and Jones *et al*., 2020. Structural predictions of IsoA (Fig. S2C) confirmed the high conservation of the α-subunit fold between Gram-positive (*Rh.* AD45) and Gram-negative (*V.* WS11) representatives, with both monomeric and dimeric models showing nearly identical core helices. Superposition highlighted subtle clade-specific loop variations, while the di-iron cluster ligating residues remained invariant. AlphaFold2 models yielded high-confidence predictions (average pLDDT ~91.8), with particularly strong confidence at regions likely to be involved in di-iron binding motifs and catalytic helices (highlighted in pink/red ribbon representation in the monomer for clarity). Together, these features underscore the strong purifying selection acting on the IsoMO catalytic core, conserved sequence of the catalytic center of the cluster and reinforce IsoA as a robust phylogenetic and functional marker.

**IsoB** (γ-subunit of the IsoMO; average length 92 amino acids, Figure S3; n=11) formed two strongly supported clades in phylogenetic trees (Fig. S3A, 89 aligned positions), clearly separating Gram-positive and Gram-negative representatives. *Rhodococcus* sequences formed a compact Gram-positive subcluster (bootstrap support >99%), while Gram-negative taxa showed longer branches, indicating greater divergence. Pairwise sequence identity was high within groups (G⁺:G⁺ 85–100%; G⁻:G⁻ 81–97%), but dropped across groups (G⁺:G⁻ <75%), consistent with taxonomic divergence across the *iso* cluster. The tree shows the second largest phylogenetic diversity (sum of branches = 4.2). Multiple sequence alignment (Fig. S3B) showed the conservation of residues 13–44 (*Rh*. AD45 numbering). Models for *Rh*. AD45 and *V.* WS11 (Fig. S3C) showed strong overlap in the central β/α framework. Confidence scores showed very high pLDDT values (>90) across the core β1–α2 region and moderate scores (70–85) in the variable loops, supporting confident prediction of conserved structural features while acknowledging flexibility in less-conserved regions.

**IsoC** (the Rieske-type ferredoxin; average length 111 amino acids, Figure S4; n=11) was among the most conserved components of the IsoMO complex, with pairwise identities ranging from 88–100% within Gram-positive strains and 85–100% within Gram-negatives. Phylogenetic analysis (Fig. S4A, 112 aligned positions) recovered strongly supported separation into Gram-positive and Gram-negative clades, with short internal branches indicative of limited divergence and recent common ancestry. Multiple sequence alignment (Fig. S4B) revealed complete conservation of several hallmark Rieske motifs across all taxa, including the CPHQ segment (C49–Q52) and the TCRAH motif (T67–H71) which coordinate the [2Fe-2S] cluster (Davidson *et al*., 1992; Colbert *et al*., 2000). Structural predictions (Fig. S4C) confirmed the near-identical fold of IsoC in both *Rh*. AD45 and *V*. WS11, with conserved packing of the β-sheet core and outward-facing α-helices. All conserved residues fell within high-confidence regions (pLDDT >95) in AlphaFold2 models, supporting their structural and functional significance.

**IsoD** (the coupling subunit; average length 110 amino acids, Figure S5, n = 11) exhibited high conservation across taxa, forming distinct Gram-positive and Gram-negative clades in the phylogenetic tree (Fig. S5A, 104 aligned positions). Within the *Rhodococcus* clade, sequences were nearly identical, reflected by short branch lengths. Multiple sequence alignment (Fig. S5B) showed strict conservation of a glutamate-rich segment (E72–E81, *Rh.* AD45 numbering) and adjacent β-strand/α-helix elements that may contribute to docking interactions with the monooxygenase as seen in the phenol hydroxylase and regulatory protein complex for *Pseudomonas* sp. OX1 (Sazinsky *et al*., 2007). Beyond this conserved block, variation was largely confined to surface-exposed loops. AlphaFold2 structural predictions (Fig. S5C) confirmed that IsoD adopts a consistent β-sheet/α-helix scaffold across both Gram-positive and Gram-negative representatives, with the glutamate-rich region and surrounding secondary structure predicted with high confidence (pLDDT > 92). These features support the role of IsoD as a structurally stable, evolutionarily constrained coupling protein that mediates electron transfer complex assembly rather than acting as a redox-active subunit itself.

**IsoE** (β-subunit of the IsoMO; average length 348 amino acids, Figure S6, n = 11) exhibited a deep G⁺/G⁻ split, with intra-clade identities of 80–100%, but G⁺:G⁻ values dropping to 47–54%. Phylogenetic trees resolved *Rhodococcus* sequences as a tight G⁺ subcluster with short internal branch lengths, whereas G⁻ sequences displayed longer branches, consistent with higher divergence (Fig. S6A, 339 aligned positions). Multiple sequence alignment (Fig. S6B) highlighted a conserved β-strand-rich region (residues 148–180, *Rh.* AD45 numbering) and a short glycine-rich segment near positions ~45–55 that is conserved (Walters *et al*., 1999). Structural predictions (Fig. S6C) showed a highly conserved β-subunit scaffold with near-identical core β-sheet/α-helix architecture across *Rh.* AD45 and *V.* WS11; clade-specific differences were confined to surface-exposed loops. Per-residue model confidence was highest in the core scaffold (pLDDT >95), underscoring structural conservation of the β-subunit.

**IsoF** (the reductase; average length 343 amino acids, Figure S7, n = 11) was the least conserved of the electron transfer components, with inter-group amino acid identities of 39–48%. This was confirmed by the largest phylogenetic diversity based on its phylogeny (sum of branches = 6.24). Phylogenetic analyses (Fig. S7A, 332 aligned positions) resolved deep Gram-positive and Gram-negative clades, with long internal branches observed particularly among G⁻ sequences. Despite this divergence, the core β-sheet/α-helix scaffold was preserved across all taxa. Multiple sequence alignment (Fig. S7B) revealed strict conservation of motifs within IsoF sequences, including a conserved YECASG segment (residues 35–40, *Rh.* AD45 numbering), a CGxC motif (42–45), and the WAD motif (58–60), all of which were invariant across sequences and may be critical for maintaining structural integrity and mediating redox interactions (e.g., Sevrioukova *et al*., 2004). Sequence divergence was concentrated in loop regions and the C-terminal tail, where Gram-negative taxa exhibited clade-specific length variation and compositional changes. Structural predictions (Fig. S7C) confirmed that these variable segments correspond to solvent-exposed regions, leaving the conserved core intact. AlphaFold2 models for *Rh.* AD45 and *V.* WS11 IsoF showed high pLDDT scores (>80) in the conserved scaffold and reduced confidence in peripheral loops, supporting the structural stability of the core and flexibility of accessory regions.

**IsoG** (putative CoA-transferase, average length 381 amino acids, Fig. S8, n = 10 full-length sequences in this dataset) resolved into well-supported Gram-positive and Gram-negative clades, with *Rhodococcus* sequences clustering tightly within the Gram-positive group (Fig. S8A, 398 aligned positions). Consistent with pairwise identities (G⁺:G⁻ 49.4–60.7%), the Gram split was accompanied by longer internal branches among Gram-negative sequences. Multiple sequence alignment (Fig. S8B) revealed several conserved features characteristic of CoA-transferases. These include the candidate glutamate residues at positions E33 and E392, or D165 from the enzyme family, as conserved in all sequences, that may be involved in the CoA transfer of Frc CoA transferases (Hackmann, 2022; *Rix et al*., 2023). Additionally, the GxG-like motifs at G36, G101 and G121 were identified. These motifs were conserved across clades, while lineage-specific indels and substitutions occurred primarily in peripheral loops. AlphaFold superpositions of IsoG from *Rh.* AD45 and *V.* WS11 predicted nearly identical α/β-domain folds and conserved active-site geometry, with structural variation largely confined to solvent-exposed surface loops (Fig. S8C). Conserved active-site residues were modeled with high confidence (pLDDT >95), supporting IsoG’s functional annotation as a CoA-transferase in the isoprene degradation pathway.

**IsoH** (NAD⁺-dependent dehydrogenase subunit; average length 226 amino acids, Figure S9, n = 11) resolved into clearly separated Gram-positive and Gram-negative clades, with evidence of recent duplication in Gram-positive taxa. *Rhodococcus* sequences formed a tight Gram-positive subcluster with short internal branches and strong bootstrap support, whereas Gram-negative sequences showed greater divergence (Fig. S9A). Despite this variation, multiple sequence alignment (Fig. S9B) and structural predictions using AlphaFold2 (Fig. S9C) showed nearly identical β-sheet/α-helix cores in *Rh.* AD45 and *V.* WS11 IsoH, with differences confined to solvent-exposed loops. Model confidence was high (mean pLDDT ~91 for AD45 and ~89 for WS11), supporting a conserved fold and cofactor-binding architecture despite clade-specific sequence divergence.

**IsoI** (glutathione S-transferase–like subunit, average length 238 amino acids, Figure S10; n = 11), formed strongly supported Gram-positive and Gram-negative clades (Fig. S10A). Pairwise identities were lower between clades (45–53%) than within clades, consistent with functional divergence. Multiple sequence alignment (Fig. S10B) revealed several highly conserved glycine- and polar-rich segments, including a canonical GXG loop (G157–G159, *Rh.* AD45 numbering) implicated in glutathione binding, as well as conserved core β-strands and α-helices typical of GST enzymes (Wilce *et al*., 1994). Clade-specific divergence was concentrated in surface-exposed loops, particularly among Gram-negative sequences, where lineage-specific insertions and polarity shifts were observed. AlphaFold2 structural models (Fig. S10C) confirmed preservation of the canonical GST α/β scaffold in both *Rh.* AD45 and *V.* WS11, with high per-residue confidence (pLDDT >90 across ~92% of residues). These results support classification of IsoI as a structurally conserved GST-like enzyme with peripheral adaptations across clades.

**IsoJ** (glutathione S-transferase–like subunit, average length 238 amino acids, Figure S11; n = 11), phylogenetic trees resolved *Rhodococcus* sequences as a tight Gram-positive subcluster, and despite the rooting artefact imposed by the midpoint rooting on highly diverged G- compared to G+, Gram-positive and Gram-negative do form monophyletic clusters. (Fig. S11A, 237 aligned positions). Multiple sequence alignment (Fig. S11B) and AlphaFold2 models of *Rh.* AD45 and *V.* WS11 (Fig. S11C) confirmed preservation of the canonical GST β/α scaffold, with minor clade-specific deviations restricted to solvent-exposed loops. Structural confidence was high (mean pLDDT ~91 for AD45 and ~90 for WS11), supporting robust core structural conservation.

**AldH1** (aldehyde dehydrogenase, average length 465 amino acids, Figure S12; n = 10), resolved into compact G⁺ and G⁻ clades with intermediate inter-group conservation (49–54%). Phylogenetic topologies broadly mirrored organismal relationships, with Gram-positive sequences clustering tightly and Gram-negative taxa forming a distinct sister group (Fig. S12A, 474 aligned positions). Multiple sequence alignment (Fig. S12B) and structural overlays of AlphaFold2 models (Fig. S12C) confirmed conservation of the catalytic core of this protein across Gram-positive and Gram-negative homologues, with loop-level variation confined to solvent-exposed regions (Ahvazi *et al*., 2000). Model confidence was uniformly high (mean pLDDT >90 for core β/α elements, 70–85 in loops), supporting the assignment of AldH1 as a catalytically competent NAD⁺-dependent aldehyde dehydrogenase that plays a more auxiliary role relative to the IsoMO core components.

When looking at the MDS plot (Fig. S15): left panels are topology only, right panels account for branch lengths. IsoA and IsoB are topologically very similar (confirmed by looking at individual trees, only *Nocardioides* location changes). And both are not far from IsoF when branches are considered (same quadrant in panels at the right): despite the topology being more different. Another grouping appears between IsoH, IsoI, and IsoG topologically. And this is joined by isoC and AldH1 looking at branch lengths. That is, even though the topologies (branching patterns) are different, the branch lengths are relatively similar.

The heatmap (Fig. S13) is less visual than the phylogenies, but allows to see actual pairwise gene distances (in terms of their evolutionary history). For instance, we see that IsoA and IsoB tend to be close to each other irrespective of distance. Same thing between IsoE and IsoF, but not between IsoC and IsoD. IsoE and AldH1 tend to be close to all other trees (their rows or columns are closer to light blue than to brown).

**References:**

**Ahvazi B,** Coulombe R, Delarge M, *et al.* Crystal structure of the NADP+-dependent aldehyde dehydrogenase from *Vibrio harveyi:* structural implications for cofactor specificity and affinity. *Biochem J* 2000; **349**:853-861. <https://doi.org/10.1042/bj3490853>

**Colbert CL,** Couture MM-J, Eltis LD, *et al.* A cluster exposed structure of the Rieske ferredoxin from biphenyl dioxygenase and the redox properties of Rieske Fe-S proteins. *Structure* 2000;**8**:1267–1278. <https://doi.org/10.1016/S0969-2126(00)00536-0>

**Davidson E,** Ohnishi T, Atta-Asafo-Adjei E, *et al.* Potential ligands to the [2Fe-2S] Rieske cluster of the cytochrome bc1 complex of *Rhodobacter capsulatus* probed by site-directed mutagenesis. *Biochemistry* 1992;**31**:3342–3351. <https://doi.org/10.1021/bi00128a006>

**Hackmann TJ.** Redefining the coenzyme a transferase superfamily with a large set of manually annotated proteins. Protein Sci 2022;31:864–81. <https://doi.org/10.1002/pro.4277>.

**Jones JC,** Banerjee R, Shi K, *et al.* Structural studies of the *Methylosinus trichosporium* OB3b soluble methane monooxygenase hydroxylase and regulatory component complex reveal a transient substrate tunnel. *Biochemistry* 2020;**59**:2946–2961. <https://doi.org/10.1021/acs.biochem.0c00459>

**Leahy JG,** Batchelor PJ, Morcomb SM. Evolution of the soluble diiron monooxygenases. *FEMS Microbiol Rev* 2003;**27**:449–479. <https://doi.org/10.1016/S0168-6445(03)00023-8>

**Rix GD,** Sims LP, Dawson RA, *et al.* Analysis of essential isoprene metabolic pathway proteins in *Variovorax* sp strain WS11. *Appl Environ Microbiol* 2023;**89**: e02122-22. <https://doi.org/10.1128/aem.02122-22>

**Sazinsky MH,** Dunten PW, McCormick MS, *et al.* X-ray structure of a hydroxylase-regulatory protein complex from a hydrocarbon-oxidizing multicomponent monooxygenase *Pseudomonas* sp OX1 phenol hydroxylase. *Biochemistry* 2006;**45**:15392–15404. <https://doi.org/10.1021/bi0618969>

**Sevrioukova IF,** Li H, Poulos TL. Crystal structure of putidaredoxin reductase from *Pseudomonas putida* the final structural component of the cytochrome P450cam monooxygenase. *J Mol Biol* 2004;**336**:889–902. <https://doi.org/10.1016/j.jmb.2003.12.067>

**Walters KJ,** Gassner GT, Lippard SJ, *et al.* Structure of the soluble methane monooxygenase regulatory protein B. *Proc Natl Acad Sci USA* 1999;**96**:7877–7882. <https://doi.org/10.1073/pnas.96.14.7877>

**Wilce MCJ,** Parker MW. Structure and function of glutathione S-transferases. *Biochim Biophys Acta* 1994;**1205**:1–18. <https://doi.org/10.1016/0167-4838(94)90086-8>
