# Supplementary material for: Structural and evolutionary insights into the isoprene monooxygenases
Source: FEMS Microbiol Ecol. 2026 Jan 22;102(3):fiag004. doi: 10.1093/femsec/fiag004 (PMC12917321; doi:10.1093/femsec/fiag004)
Supplement: fiag004_Supplemental_Files [file fiag004_supplemental_files.zip › UNTRACKED_revised_Supplementary_Tables_v2.docx]

**Supplementary Tables**

**Supplementary Table S1.** Isoprene-degrading bacterial strains isolated and with sequenced genomes.

| **Isolate** | **RefSeq** | **Scaffolds (contigs)** | **N50** | **L50** | **Total length (Mb)** | **Protein count** | **GC%** | **Genome neighbour (symmetrical identity; gapped)*** |
| --- | --- | --- | --- | --- | --- | --- | --- | --- |
| *Rhodococcus AD45* | GCA_000949305.1 | 9 | 1,521,985 | 2 | 6.79479 | 6029 | 61.7 |  |
| *Rhodococcus opacus PD630* | GCA_000599545.1 | 10 | 8,376,953 | 1 | 9.16903 | 8942 | 67.2 |  |
| *Rhodococcus LB1* | GCA_001583455.1 | 448 (526) | 48,257 | 62 | 10.752 | 9270 | 66.6 | *Rhodococcus* SC4 (82%) |
| *Rhodococcus SC4* | GCA_001555475.1 | 345 (419) | 71,344 | 41 | 10.5719 | 9123 | 66.7 | *Rhodococcus* LB1 (82%) |
| *Rhodococcus ACPA1* | GCA_002300195.1 | 47 (48) | 1,120,468 | 4 | 10.0608 | 8808 | 66.9 | *Rhodococcus* LB1 (81.56%) |
| *Rhodococcus ACPA4* | GCA_002300185.1 | 9 (11) | 3,616,808 | 1 | 7.06712 | 6247 | 61.1 | metagenome *Rhodococcus* sp. PMG_254 (98.78%) |
| *Rhodococcus ACS1* | GCA_002300155.1 | 40 (41) | 676,616 | 5 | 10.8914 | 9508 | 67 | *Rhodococcus* ACPA1 (64.49%) |
| *Rhodococcus WS1* | GCA_003797745.1 | 8 | 2,655,236 | 2 | 6.56024 | 5943 | 62.3 | *Rhodococcus* WS7 (99%) |
| *Rhodococcus WS3* | GCA_003797085.1 | 19 (21) | 1,535,683 | 2 | 6.85616 | 6095 | 61.7 | metagenome *Rhodococcus* sp. PMG_254 (98.82%) |
| *Rhodococcus WS4* | GCA_006543605.1 | 1221 | 40,486 | 91 | 12.7295 | 10854 | 66.4 | *Rhodococcus* ACPA1 (56.96%) |
| *Rhodococcus WS7* | GCA_006543615.1 | 180 | 453,949 | 5 | 6.6282 | 6053 | 62.4 | *Rhodococcus* WS1 (99.34%) |
| *Gordonia i37* | GCA_002043085.1 | 721 | 16524 | 122 | 6.22816 | 5486 | 66.8 | *Gordonia* sp. NB4-1Y (84.00%; 97.44%) |
| *Gordonia OPL2* | GCA_003797825.1 | 146 | 149,633 | 11 | 5.7959 | 5169 | 67.3 |  |
| *Mycobacterium AT1* | GCA_002043095.1 | 129 (143) | 105263 | 22 | 7.07238 | 6620 | 67.2 | *Mycobacterium* sp. Root135 (73.85%) |
| *Nocardioides WS12* | GCA_014108865.1 | 1 | 5,171,066 | 1 | 5.17107 | 4925 | 68.7 | *Nocardioides* sp. Root682 (64.35%; 88.15%) |
| *Ramlibacter WS9* | GCA_003797765.1 | 180 | 224764 | 10 | 6.98085 | 6517 | 65.4 |  |
| *Variovorax WS11* | GCA_010499245.1 | 5 | 1304680 | 2 | 8.48961 | 7789 | 67.3901 | *Variovorax* sp CF079 (54.52%; 87.31%)  *Variovorax* RA8 (51.8%; 90.121%) |
| *Sphingopyxis OPL5* | GCA_003797775.2 | 1 | 4,676,975 | 1 | 4.67697 | 4418 | 65.9 |  |

*The listed bacterial name corresponds to the closest genome neighbour identified in the alignment-cased comparison. The percentages indicate the symmetrical identity between the two genomes, calculated for the whole genome and with gaps included.

**Supplementary Table S2.** Protein lengths of isoprene metabolic cluster genes (IsoA–F, IsoG–J, and AldH1) across 11 selected isoprene-degrading bacterial genomes.

| **Genus** | **strain** | **IsoG** | **IsoH** | **IsoI** | **IsoJ** | **AldH** | **IsoA** | **IsoB** | **IsoC** | **IsoD** | **IsoE** | **IsoF** |
| --- | --- | --- | --- | --- | --- | --- | --- | --- | --- | --- | --- | --- |
| ***Rhodococcus*** | **AD45** | 401 | 226 | 238 | 233 | -* | 514 | 94 | 114 | 110 | 342 | 345 |
| ***Rhodococcus*** | **PD630** | 405 | 226 | 238 | 233 | 451 | 507 | 94 | 114 | 110 | 340 | 345 |
| ***Rhodococcus*** | **LB1** | 173 | 226 | 238 | 233 | 451 | 507 | 94 | 114 | 110 | 340 | 345 |
| ***Rhodococcus*** | **WS7** | 401 | 226 | 238 | 233 | 451 | 507 | 94 | 114 | 110 | 340 | 345 |
| ***Mycobacterium*** | **AT1** | 401 | 226 | 238 | 234 | 450 | 511 | 94 | 114 | 108 | 342 | 331 |
| ***Gordonia*** | **i37** | 402 | 226 | 239 | 236 | 477 | 506 | 95 | 121 | 106 | 338 | 341 |
| ***Gordonia*** | **OPL2** | 401 | 226 | 239 | 236 | 478 | 496 | 95 | 109 | 106 | 340 | 342 |
| ***Nocardioides*** | **WS12** | 395 | 226 | 237 | 250 | 477 | 498 | 89 | 118 | 110 | 340 | 355 |
| ***Ramlibacter*** | **WS9** | 402 | 226 | 238 | 239 | 465 | 497 | 88 | 97 | 104 | 343 | 342 |
| ***Sphingopyxis*** | **OPL5** | 405 | 226 | 225 | 243 | 466 | 497 | 87 | 112 | 106 | 367 | 329 |
| ***Variovorax*** | **WS11** | 402 | 227 | 238 | 242 | 475 | 500 | 88 | 111 | 97 | 367 | 344 |

**Supplementary Table S3.** Pairwise amino acid identity percentages for each protein in the isoprene metabolic gene cluster (IsoA–F, IsoG–J, and AldH) across 11 isoprene-degrading strains. Values were derived from BLASTp comparisons.

| 100-90 | 89-80 | 79-70 | 69-60 | 59-50 | 49-35 | 34-0 |
| --- | --- | --- | --- | --- | --- | --- |

| **IsoA** | **AD45** | **PD630** | **LB1** | **WS7** | **AT1** | **i37** | **OPL2** | **WS12** | **WS9** | **WS11** | **OPL5** |  | **IsoG** | **AD45** | **PD630** | **LB1** | **WS7** | **AT1** | **i37** | **OPL2** | **WS12** | **WS9** | **WS11** | **OPL5** |
| --- | --- | --- | --- | --- | --- | --- | --- | --- | --- | --- | --- | --- | --- | --- | --- | --- | --- | --- | --- | --- | --- | --- | --- | --- |
| **AD45** | 100 |  |  |  |  |  |  |  |  |  |  |  | **AD45** | 100 |  |  |  |  |  |  |  |  |  |  |
| **PD630** | 91.1 | 100 |  |  |  |  |  |  |  |  |  |  | **PD630** | 90.5 | 100 |  |  |  |  |  |  |  |  |  |
| **LB1** | 91.3 | 99.4 | 100 |  |  |  |  |  |  |  |  |  | **LB1** | 83.3 | 80.4 | 100 |  |  |  |  |  |  |  |  |
| **WS7** | 90.7 | 93.5 | 93.9 | 100 |  |  |  |  |  |  |  |  | **WS7** | 90 | 95.8 | 78.6 | 100 |  |  |  |  |  |  |  |
| **AT1** | 83.2 | 88.2 | 85.5 | 85.5 | 100 |  |  |  |  |  |  |  | **AT1** | 84.3 | 87.8 | 76.3 | 86.5 | 100 |  |  |  |  |  |  |
| **i37** | 85.6 | 90.6 | 88.8 | 89 | 85.9 | 100 |  |  |  |  |  |  | **i37** | 78.9 | 82.6 | 70.8 | 83.5 | 81.3 | 100 |  |  |  |  |  |
| **OPL2** | 86.7 | 90 | 89.7 | 89.3 | 86.3 | 92.5 | 100 |  |  |  |  |  | **OPL2** | 77.5 | 80.8 | 68.3 | 80.8 | 80.0 | 89.8 | 100 |  |  |  |  |
| **WS12** | 83.9 | 87.9 | 87.9 | 86.1 | 82.4 | 84.7 | 84.7 | 100 |  |  |  |  | **WS12** | 75.3 | 76.7 | 68.3 | 76 | 74.5 | 74.8 | 73.9 | 100 |  |  |  |
| **WS9** | 71.2 | 73.7 | 73.7 | 72.7 | 73.3 | 74.6 | 72.3 | 72 | 100 |  |  |  | **WS9** | 60.7 | 60.4 | 57.9 | 59.1 | 60.5 | 56.5 | 56.2 | 59.4 | 100 |  |  |
| **WS11** | 72.8 | 74.5 | 74.5 | 73.5 | 74.1 | 74.6 | 72.9 | 73.1 | 91.8 | 100 |  |  | **WS11** | 59.2 | 57.5 | 53.1 | 56.3 | 56.0 | 55.5 | 54.6 | 56.3 | 84.5 | 100 |  |
| **OPL5** | 72.2 | 74.5 | 74.5 | 73.5 | 73.0 | 75 | 73.1 | 71.3 | 81.1 | 81.6 | 100 |  | **OPL5** | 60.3 | 58.8 | 49.4 | 58.4 | 58.0 | 55.8 | 54.7 | 58.5 | 67.3 | 65 | 100 |

| **IsoB** | **AD45** | **PD630** | **LB1** | **WS7** | **AT1** | **i37** | **OPL2** | **WS12** | **WS9** | **WS11** | **OPL5** |  | **IsoH** | **AD45** | **PD630** | **LB1** | **WS7** | **AT1** | **i37** | **OPL2** | **WS12** | **WS9** | **WS11** | **OPL5** |
| --- | --- | --- | --- | --- | --- | --- | --- | --- | --- | --- | --- | --- | --- | --- | --- | --- | --- | --- | --- | --- | --- | --- | --- | --- |
| **AD45** | 100 |  |  |  |  |  |  |  |  |  |  |  | **AD45** | 100 |  |  |  |  |  |  |  |  |  |  |
| **PD630** | 84.0 | 100 |  |  |  |  |  |  |  |  |  |  | **PD630** | 86.3 | 100 |  |  |  |  |  |  |  |  |  |
| **LB1** | 83 | 98.9 | 100 |  |  |  |  |  |  |  |  |  | **LB1** | 87.2 | 94.7 | 100 |  |  |  |  |  |  |  |  |
| **WS7** | 76.9 | 82.4 | 84.3 | 100 |  |  |  |  |  |  |  |  | **WS7** | 88.1 | 91.2 | 91.6 | 100 |  |  |  |  |  |  |  |
| **AT1** | 66.7 | 68.8 | 68.8 | 62.8 | 100 |  |  |  |  |  |  |  | **AT1** | 78.3 | 82.7 | 81.9 | 78.8 | 100 |  |  |  |  |  |  |
| **i37** | 57.5 | 60.6 | 61.7 | 58.1 | 65.6 | 100 |  |  |  |  |  |  | **i37** | 79.2 | 82.7 | 81 | 79.7 | 82.3 | 100 |  |  |  |  |  |
| **OPL2** | 65.9 | 64.8 | 64.8 | 59.8 | 66.7 | 83.2 | 100 |  |  |  |  |  | **OPL2** | 81.4 | 83.2 | 82.7 | 81.4 | 83.2 | 85.4 | 100 |  |  |  |  |
| **WS12** | 55.7 | 58 | 56.8 | 50 | 56.2 | 55.7 | 56.8 | 100 |  |  |  |  | **WS12** | 73.5 | 70.8 | 73.0 | 70.4 | 68.6 | 70.8 | 69.9 | 100 |  |  |  |
| **WS9** | 50 | 50 | 50 | 52.3 | 51.7 | 46.6 | 50 | 42.1 | 100 |  |  |  | **WS9** | 61.5 | 61.5 | 61.5 | 61.1 | 61.5 | 60.6 | 59.7 | 64.6 | 100 |  |  |
| **WS11** | 53.6 | 54.8 | 54.8 | 55.8 | 54.8 | 45.2 | 46.4 | 39.3 | 73.3 | 100 |  |  | **WS11** | 59.1 | 59.6 | 60.4 | 58.2 | 60.8 | 58.7 | 59.4 | 63.6 | 82.2 | 100 |  |
| **OPL5** | 44.3 | 46.6 | 46.6 | 43.2 | 47.7 | 47.7 | 45.4 | 39.5 | 50 | 52.3 | 100 |  | **OPL5** | 61.1 | 60.2 | 62.4 | 61.5 | 63.3 | 59.7 | 59.7 | 60.6 | 60.2 | 60.8 | 100 |
|  |  |  |  |  |  |  |  |  |  |  |  |  |  |  |  |  |  |  |  |  |  |  |  |  |
| **IsoC** | **AD45** | **PD630** | **LB1** | **WS7** | **AT1** | **i37** | **OPL2** | **WS12** | **WS9** | **WS11** | **OPL5** |  | **IsoI** | **AD45** | **PD630** | **LB1** | **WS7** | **AT1** | **i37** | **OPL2** | **WS12** | **WS9** | **WS11** | **OPL5** |
| **AD45** | 100 |  |  |  |  |  |  |  |  |  |  |  | **AD45** | 100 |  |  |  |  |  |  |  |  |  |  |
| **PD630** | 86 | 100 |  |  |  |  |  |  |  |  |  |  | **PD630** | 85.7 | 100 |  |  |  |  |  |  |  |  |  |
| **LB1** | 86 | 100 | 100 |  |  |  |  |  |  |  |  |  | **LB1** | 76.1 | 80.3 | 100 |  |  |  |  |  |  |  |  |
| **WS7** | 82.5 | 89.5 | 89.5 | 100 |  |  |  |  |  |  |  |  | **WS7** | 85.7 | 91.6 | 76.5 | 100 |  |  |  |  |  |  |  |
| **AT1** | 71.7 | 76.1 | 76.1 | 89.4 | 100 |  |  |  |  |  |  |  | **AT1** | 82.4 | 87 | 76.1 | 87 | 100 |  |  |  |  |  |  |
| **i37** | 78.1 | 84.9 | 84.9 | 84.5 | 78.9 | 100 |  |  |  |  |  |  | **i37** | 80.1 | 84.3 | 71.2 | 86 | 83.5 | 100 |  |  |  |  |  |
| **OPL2** | 81.3 | 87.9 | 87.9 | 89.4 | 73.6 | 87.5 | 100 |  |  |  |  |  | **OPL2** | 78.9 | 83.1 | 72.2 | 84.8 | 83.1 | 95.4 | 100 |  |  |  |  |
| **WS12** | 62.2 | 65.5 | 65.5 | 63.9 | 64.0 | 70.5 | 66.7 | 100 |  |  |  |  | **WS12** | 65.6 | 67.2 | 66.4 | 65.1 | 63.5 | 61.9 | 62.5 | 100 |  |  |  |
| **WS9** | 58.7 | 59.0 | 59 | 59.5 | 58.3 | 58.3 | 59.5 | 55.3 | 100 |  |  |  | **WS9** | 47.5 | 52.1 | 48.3 | 50.4 | 50.8 | 47.0 | 47.7 | 47.1 | 100 |  |  |
| **WS11** | 49.1 | 48.7 | 48.7 | 50.5 | 49.6 | 56 | 57.3 | 51.6 | 60.4 | 100 |  |  | **WS11** | 47.9 | 53.4 | 50 | 52.1 | 51.7 | 49.2 | 49.8 | 48.3 | 79.8 | 100 |  |
| **OPL5** | 54.2 | 52.5 | 52.5 | 54.2 | 50 | 53.1 | 57.3 | 56.3 | 50.6 | 50.5 | 100 |  | **OPL5** | 43.5 | 47.2 | 45.8 | 46.3 | 47.7 | 46.3 | 46.7 | 45.4 | 50 | 51.6 | 100 |

|  |  |  |  |  |  |  |  |  |  |  |  |  |  |  |  |  |  |  |  |  |  |  |  |  |
| --- | --- | --- | --- | --- | --- | --- | --- | --- | --- | --- | --- | --- | --- | --- | --- | --- | --- | --- | --- | --- | --- | --- | --- | --- |
| **IsoD** | **AD45** | **PD630** | **LB1** | **WS7** | **AT1** | **i37** | **OPL2** | **WS12** | **WS9** | **WS11** | **OPL5** |  | **IsoJ** | **AD45** | **PD630** | **LB1** | **WS7** | **AT1** | **i37** | **OPL2** | **WS12** | **WS9** | **WS11** | **OPL5** |
| **AD45** | 100 |  |  |  |  |  |  |  |  |  |  |  | **AD45** | 100 |  |  |  |  |  |  |  |  |  |  |
| **PD630** | 96.4 | 100 |  |  |  |  |  |  |  |  |  |  | **PD630** | 77.6 | 100 |  |  |  |  |  |  |  |  |  |
| **LB1** | 96.4 | 100 | 100 |  |  |  |  |  |  |  |  |  | **LB1** | 78 | 97.9 | 100 |  |  |  |  |  |  |  |  |
| **WS7** | 93.6 | 95.5 | 95.5 | 100 |  |  |  |  |  |  |  |  | **WS7** | 77.2 | 90.5 | 92.2 | 100 |  |  |  |  |  |  |  |
| **AT1** | 82.4 | 82.4 | 82.4 | 82.4 | 100 |  |  |  |  |  |  |  | **AT1** | 73.5 | 80.6 | 80.6 | 77.6 | 100 |  |  |  |  |  |  |
| **i37** | 71.2 | 72.1 | 72.1 | 71.15 | 72.6 | 100 |  |  |  |  |  |  | **i37** | 71.2 | 73.2 | 74 | 74.2 | 70 | 100 |  |  |  |  |  |
| **OPL2** | 73.1 | 72.6 | 71.6 | 70.8 | 70.8 | 83.0 | 100 |  |  |  |  |  | **OPL2** | 68 | 69.1 | 69.1 | 67.8 | 66.8 | 80.8 | 100 |  |  |  |  |
| **WS12** | 65.1 | 65.1 | 65.1 | 67 | 63.3 | 65.4 | 68.6 | 100 |  |  |  |  | **WS12** | 66.4 | 67.3 | 67.7 | 68.1 | 64.1 | 65.1 | 64.2 | 100 |  |  |  |
| **WS9** | 55.2 | 54.2 | 54.2 | 53.9 | 57.8 | 59.1 | 61.4 | 60.2 | 100 |  |  |  | **WS9** | 54.8 | 55.7 | 56.6 | 58.8 | 51.5 | 55 | 52.8 | 56.8 | 100 |  |  |
| **WS11** | 58.3 | 56.3 | 56.3 | 56.7 | 57.8 | 54.4 | 61.4 | 55.9 | 81.4 | 100 |  |  | **WS11** | 54.8 | 57.9 | 57.9 | 57 | 52.5 | 52.8 | 55.9 | 54.6 | 77 | 100 |  |
| **OPL5** | 50 | 48.9 | 48.9 | 50 | 46.8 | 50 | 52.1 | 57.7 | 57.7 | 58.9 | 100 |  | **OPL5** | 58.8 | 62 | 62 | 61.6 | 59.5 | 60.7 | 60.3 | 56.1 | 53 | 52.7 | 100 |
|  |  |  |  |  |  |  |  |  |  |  |  |  |  |  |  |  |  |  |  |  |  |  |  |  |
| **IsoE** | **AD45** | **PD630** | **LB1** | **WS7** | **AT1** | **i37** | **OPL2** | **WS12** | **WS9** | **WS11** | **OPL5** |  | **AldH1** | **AD45** | **PD630** | **LB1** | **WS7** | **AT1** | **i37** | **OPL2** | **WS12** | **WS9** | **WS11** | **OPL5** |
| **AD45** | 100 |  |  |  |  |  |  |  |  |  |  |  | **PD630** | - | 100 |  |  |  |  |  |  |  |  |  |
| **PD630** | 84.2 | 100 |  |  |  |  |  |  |  |  |  |  | **LB1** | - | 99.6 | 100 |  |  |  |  |  |  |  |  |
| **LB1** | 84.2 | 100 | 100 |  |  |  |  |  |  |  |  |  | **WS7** | - | 92 | 92 | 100 |  |  |  |  |  |  |  |
| **WS7** | 83.0 | 87.9 | 87.9 | 100 |  |  |  |  |  |  |  |  | **AT1** | - | 82.2 | 82 | 80 | 100 |  |  |  |  |  |  |
| **AT1** | 74.5 | 76.3 | 76.3 | 75.8 | 100 |  |  |  |  |  |  |  | **i37** | - | 76.4 | 76.4 | 76.6 | 77.5 | 100 |  |  |  |  |  |
| **i37** | 71.5 | 73.1 | 73.1 | 71.3 | 70.1 | 100 |  |  |  |  |  |  | **OPL2** | - | 75.5 | 75.7 | 75.7 | 72.8 | 82.2 | 100 |  |  |  |  |
| **OPL2** | 68.1 | 71.3 | 71.3 | 69.2 | 68.3 | 79 | 100 |  |  |  |  |  | **WS12** | - | 67.8 | 67.8 | 65.8 | 68.8 | 63.6 | 64.1 | 100 |  |  |  |
| **WS12** | 61.1 | 62.2 | 62.2 | 61.9 | 60.2 | 59.8 | 58.9 | 100 |  |  |  |  | **WS9** | - | 52.1 | 51.9 | 50.6 | 52.6 | 49.5 | 50.7 | 54.1 | 100 |  |  |
| **WS9** | 53.3 | 53 | 53 | 52.7 | 52.8 | 52.1 | 51.8 | 49.6 | 100 |  |  |  | **WS11** | - | 53.8 | 53.6 | 52.9 | 53.6 | 49.2 | 50.8 | 53.1 | 81.4 | 100 |  |
| **WS11** | 52.8 | 51.6 | 51.6 | 51.8 | 54.1 | 52.2 | 52.3 | 47.3 | 72.6 | 100 |  |  | **OPL5** | - | 50.3 | 50.1 | 49.9 | 50.5 | 49.7 | 49.5 | 52 | 56.5 | 55.7 | 100 |
| **OPL5** | 53.4 | 50.8 | 50.8 | 52.7 | 47.2 | 51.1 | 50.6 | 51 | 53.3 | 54.5 | 100 |  |  |  |  |  |  |  |  |  |  |  |  |  |

|  |  |  |  |  |  |  |  |  |  |  |  |  |  |  |  |  |  |  |  |  |  |  |  |  |
| --- | --- | --- | --- | --- | --- | --- | --- | --- | --- | --- | --- | --- | --- | --- | --- | --- | --- | --- | --- | --- | --- | --- | --- | --- |
| **IsoF** | **AD45** | **PD630** | **LB1** | **WS7** | **AT1** | **i37** | **OPL2** | **WS12** | **WS9** | **WS11** | **OPL5** |  |  |  |  |  |  |  |  |  |  |  |  |  |
| **AD45** | 100 |  |  |  |  |  |  |  |  |  |  |  |  |  |  |  |  |  |  |  |  |  |  |  |
| **PD630** | 81.7 | 100 |  |  |  |  |  |  |  |  |  |  |  |  |  |  |  |  |  |  |  |  |  |  |
| **LB1** | 81.7 | 100 | 100 |  |  |  |  |  |  |  |  |  |  |  |  |  |  |  |  |  |  |  |  |  |
| **WS7** | 77.7 | 89.3 | 89.3 | 100 |  |  |  |  |  |  |  |  |  |  |  |  |  |  |  |  |  |  |  |  |
| **AT1** | 66 | 66 | 66 | 64.7 | 100 |  |  |  |  |  |  |  |  |  |  |  |  |  |  |  |  |  |  |  |
| **i37** | 60 | 62.3 | 62.3 | 62 | 60.4 | 100 |  |  |  |  |  |  |  |  |  |  |  |  |  |  |  |  |  |  |
| **OPL2** | 60.6 | 64.1 | 64.1 | 64.5 | 59.8 | 69.3 | 100 |  |  |  |  |  |  |  |  |  |  |  |  |  |  |  |  |  |
| **WS12** | 48.6 | 50 | 50 | 50.9 | 49.1 | 52.5 | 51.2 | 100 |  |  |  |  |  |  |  |  |  |  |  |  |  |  |  |  |
| **WS9** | 43.2 | 43.6 | 43.6 | 43.8 | 43.7 | 47.7 | 47.1 | 41.3 | 100 |  |  |  |  |  |  |  |  |  |  |  |  |  |  |  |
| **WS11** | 41.4 | 40.8 | 40.8 | 42.3 | 423 | 45.1 | 42.3 | 39.3 | 61.2 | 100 |  |  |  |  |  |  |  |  |  |  |  |  |  |  |
| **OPL5** | 43.8 | 44.2 | 44.2 | 46.3 | 43.3 | 45.3 | 44.2 | 40 | 50.5 | 44.4 | 100 |  |  |  |  |  |  |  |  |  |  |  |  |  |

**Supplementary Table S4.** Summary of BLASTp pairwise protein identity ranges (%) for each isoprene metabolic gene categorized by taxonomic group comparison. Reported values represent the observed minimum and maximum identity percentages for each gene across G⁺:G⁺, G⁻:G⁻, and G⁺:G⁻ pairings.

|  | **Percentage homology (%)** | | |
| --- | --- | --- | --- |
| **Protein** | **G+:G+** | **G+:G-** | **G-:G-** |
| **IsoA** | 82.4-100 | 71.2-74.6 | 81-100 |
| **IsoE** | 60.2-100 | 47.3-54.1 | 53.2-100 |
| **IsoF** | 48.5-100 | 39.3-47.7 | 44.4-100 |
| **IsoG** | 68.3-100 | 49.4-60.7 | 65-100 |
| **IsoH** | 68.6-100 | 58.2-63.6 | 60.2-100 |
| **IsoI** | 61.9-100 | 45.4-53.4 | 50-100 |
| **IsoJ** | 64.1-100 | 51.5-62 | 52.7-100 |
| **AldH1** | 63.6-100 | 49.2-54.1 | 55.7-100 |

**Supplementary Table S5**. List of protein sequences from α-subunits of soluble di-iron monooxygenases (SDIMOs) used in the multiple sequence alignment, including all IsoA sequences from this study and selected non-IsoA SDIMO representatives. * are sequences included in the MmoX alignment with IsoA.

| **Gene** | **Organism** | **Genome accesion No** | **GenBank Protein ID** | **Protein length** |
| --- | --- | --- | --- | --- |
| MmoX* | *Methylocella tundrae* T4 | AJ555245.1 | CAD88243.1 | 398 |
| MmoX* | *Methylocella silvestris BL2* | AJ491848.1 | CAD37188.1 | 395 |
| MmoX* | *Methylocella palustris* BL2 | AJ458535.1 | CAD30366.1 | 397 |
| MmoX* | *Methylosinus trichosporium* Ob3b | X55934.3 | CAA39068.2 | 526 |
| MmoX* | *Methylococcus capsulatus* (Bath) | M90050.3 | AAB62392.3 | 527 |
| MmoX* | *Methylomonas methanica* MC09 | CP002738.1 | AEG00068.1 | 527 |
| BmoX | *Thauera butanivorans* | AY093933.3 | AAM19727.1 | 530 |
| ThmA | *Pseudonocardia tetrahydrofuranoxydans* | AJ296087.1 | CAC10506.1 | 545 |
| PrmA | *Rhodococcus sp. RR1* | HM209445.1 | ADM83577.1 | 439 |
| PrmA | *Gordonia sp. TY-5* | AB112920.1 | BAD03956.2 | 545 |
| EtnC | *Nocardioides sp. JS614* | AY772007.1 | AAV52084.1 | 501 |
| EtnC | *Mycolicibacterium chubuense NBB4* | GU174752.1 | ACZ56346.1 | 514 |
| EtnC | *Mycolicibacterium rhodesiae JS60* | AY243034.1 | AAO48576.1 | 500 |
| TmoA3 | *Burkholderia cepacia* G4 | AF349675.1 | AAL50373.1 | 519 |
| TmoA | *Pseudomonas mendocina* KR1 | M65106.1 | AAA25999.1 | 500 |
| TbuA | *Ralstonia picketii* | U04052.1 | AAB09618.1 | 501 |
| XamoA | *Xanthomonas autotrophicus Py2* | AJ012090.1 | CAA09911.1 | 497 |
| IsoA | *Rhodococcus* sp. *AD45* | JYOP01000009.1 | KJF19164.1 | 507 |
| IsoA | *Rhodococcus opacus PD630* | JH377098.1 | EHI47089.1 | 507 |
| IsoA | *Rhodococcus* sp. *LB1* | LTCZ01000014.1 | KXX62729.1 | 507 |
| IsoA | *Rhodococcus* sp. *WS7* | SCFU01000009.1 | TQC36059.1 | 507 |
| IsoA | *Mycobacterium* sp.  *AT1* | MVOC01000096.1 | OPX05314.1 | 511 |
| IsoA | *Gordonia* sp. *i37* | MVPX01000257.1 | OPX14991.1 | 506 |
| IsoA | *Gordonia* sp. *OPL2* | RKME01000032.1 | ROZ88020.1 | 496 |
| IsoA | *Nocardioides* sp. *WS12* | CP053928.1 | WP_182378907.1 | 498 |
| IsoA | *Ramlibacter* sp. *WS9* | MK176346.1 | AZL41290.1 | 497 |
| IsoA | *Variovorax* sp. *WS11* | JAAGOW010000004.1 | NDZ17408.1 | 500 |
| IsoA | *Sphingopyxis* sp. *OPL5* | CP060725.1 | QNO27218.1 | 497 |

**Supplementary Table S6.** Pairwise nucleotide identity percentages for each protein-coding in the isoprene metabolic gene cluster (IsoA–F, IsoG–J, and AldH) across 11 isoprene-degrading strains. Values were derived from BLASTn comparisons.

| ***isoA*** | **AD45** | **PD630** | **LB1** | **WS7** | **AT1** | **i37** | **OPL2** | **WS12** | **WS9** | **WS11** | **OPL5** |  | ***isoG*** | **AD45** | **PD630** | **LB1** | **WS7** | **AT1** | **i37** | **OPL2** | **WS12** | **WS9** | **WS11** | **OPL5** |
| --- | --- | --- | --- | --- | --- | --- | --- | --- | --- | --- | --- | --- | --- | --- | --- | --- | --- | --- | --- | --- | --- | --- | --- | --- |
| **AD45** | 100.0 |  |  |  |  |  |  |  |  |  |  |  | **AD45** | 100.0 |  |  |  |  |  |  |  |  |  |  |
| **PD630** | 84.7 | 100.0 |  |  |  |  |  |  |  |  |  |  | **PD630** | 83.5 | 100.0 |  |  |  |  |  |  |  |  |  |
| **LB1** | 84.7 | 99.4 | 100.0 |  |  |  |  |  |  |  |  |  | **LB1** | 77.3 | 76.7 | 100.0 |  |  |  |  |  |  |  |  |
| **WS7** | 84.7 | 88.7 | 88.6 | 100.0 |  |  |  |  |  |  |  |  | **WS7** | 80.9 | 87.3 | 75.0 | 100.0 |  |  |  |  |  |  |  |
| **AT1** | 78.7 | 81.8 | 82.1 | 79.7 | 100.0 |  |  |  |  |  |  |  | **AT1** | 78.1 | 80.6 | 74.7 | 79.5 | 100.0 |  |  |  |  |  |  |
| **i37** | 80.4 | 83.8 | 84.0 | 81.7 | 81.7 | 100.0 |  |  |  |  |  |  | **i37** | 74.8 | 77.6 | 70.9 | 75.3 | 78.3 | 100.0 |  |  |  |  |  |
| **OPL2** | 79.1 | 81.8 | 82.1 | 80.8 | 80.8 | 84.3 | 100.0 |  |  |  |  |  | **OPL2** | 74.9 | 77.4 | 71.6 | 74.7 | 77.3 | 79.4 | 100.0 |  |  |  |  |
| **WS12** | 78.4 | 82.7 | 82.7 | 79.5 | 79.5 | 81.8 | 79.1 | 100.0 |  |  |  |  | **WS12** | 73.5 | 77.5 | 71.9 | 75.0 | 76.7 | 73.7 | 73.1 | 100.0 |  |  |  |
| **WS9** | 73.0 | 73.4 | 73.5 | 72.7 | 72.7 | 76.3 | 73.4 | 75.4 | 100.0 |  |  |  | **WS9** | 66.1 | 68.1 |  | 65.9 | 68.1 | 65.2 | 66.7 | 71.0 | 100.0 |  |  |
| **WS11** | 73.9 | 76.0 | 76.2 | 73.5 | 73.5 | 77.2 | 73.4 | 76.7 | 87.4 | 100.0 |  |  | **WS11** | 65.9 | 66.6 | 68.3 | 66.2 | 65.9 | 68.3 | 67.7 | 71.1 | 80.3 | 100.0 |  |
| **OPL5** | 71.6 | 73.8 | 74.0 | 72.3 | 72.3 | 74.3 | 73.2 | 74.0 | 78.1 | 80.0 | 100.0 |  | **OPL5** | 66.9 | 68.5 | 69.8 | 69.2 | 70.4 | 68.2 | 67.9 | 67.6 | 70.7 | 69.8 | 100.0 |

|  |  |  |  |  |  |  |  |  |  |  |  |  |  |  |  |  |  |  |  |  |  |  |  |  |
| --- | --- | --- | --- | --- | --- | --- | --- | --- | --- | --- | --- | --- | --- | --- | --- | --- | --- | --- | --- | --- | --- | --- | --- | --- |
|  |  |  |  |  |  |  |  |  |  |  |  |  |  |  |  |  |  |  |  |  |  |  |  |  |
|  |  |  |  |  |  |  |  |  |  |  |  |  |  |  |  |  |  |  |  |  |  |  |  |  |
|  |  |  |  |  |  |  |  |  |  |  |  |  |  |  |  |  |  |  |  |  |  |  |  |  |
|  |  |  |  |  |  |  |  |  |  |  |  |  |  |  |  |  |  |  |  |  |  |  |  |  |
|  |  |  |  |  |  |  |  |  |  |  |  |  |  |  |  |  |  |  |  |  |  |  |  |  |
|  |  |  |  |  |  |  |  |  |  |  |  |  |  |  |  |  |  |  |  |  |  |  |  |  |
|  |  |  |  |  |  |  |  |  |  |  |  |  |  |  |  |  |  |  |  |  |  |  |  |  |
|  |  |  |  |  |  |  |  |  |  |  |  |  |  |  |  |  |  |  |  |  |  |  |  |  |
|  |  |  |  |  |  |  |  |  |  |  |  |  |  |  |  |  |  |  |  |  |  |  |  |  |
|  |  |  |  |  |  |  |  |  |  |  |  |  |  |  |  |  |  |  |  |  |  |  |  |  |
|  |  |  |  |  |  |  |  |  |  |  |  |  |  |  |  |  |  |  |  |  |  |  |  |  |
| **isoB** | AD45 | PD630 | LB1 | WS7 | AT1 | i37 | OPL2 | WS12 | WS9 | WS11 | OPL5 |  | **isoH** | AD45 | PD630 | LB1 | WS7 | AT1 | i37 | OPL2 | WS12 | WS9 | WS11 | OPL5 |
| **AD45** | 100.0 |  |  |  |  |  |  |  |  |  |  |  | **AD45** | 100.0 |  |  |  |  |  |  |  |  |  |  |
| **PD630** | 78.6 | 100.0 |  |  |  |  |  |  |  |  |  |  | **PD630** | 78.4 | 100.0 |  |  |  |  |  |  |  |  |  |
| **LB1** | 77.9 | 98.6 | 100.0 |  |  |  |  |  |  |  |  |  | **LB1** | 78.4 | 93.8 | 100.0 |  |  |  |  |  |  |  |  |
| **WS7** | 73.0 | 81.7 | 81.3 | 100.0 |  |  |  |  |  |  |  |  | **WS7** | 78.4 | 87.4 | 86.9 | 100.0 |  |  |  |  |  |  |  |
| **AT1** | 68.9 | 71.5 | 71.2 | 69.7 | 100.0 |  |  |  |  |  |  |  | **AT1** | 71.8 | 77.6 | 78.3 | 74.5 | 100.0 |  |  |  |  |  |  |
| **i37** |  | 69.8 | 69.4 | 69.3 | 70.3 | 100.0 |  |  |  |  |  |  | **i37** | 72.6 | 76.8 | 77.3 | 73.4 | 79.0 | 100.0 |  |  |  |  |  |
| **OPL2** | 67.8 | 70.7 | 69.6 | 78.9 | 72.0 | 77.9 | 100.0 |  |  |  |  |  | **OPL2** | 73.7 | 77.3 | 77.1 | 75.2 | 76.5 | 79.2 | 100.0 |  |  |  |  |
| **WS12** |  |  |  |  | 65.9 | 77.1 | 65.2 | 100.0 |  |  |  |  | **WS12** | 69.9 | 73.0 | 74.8 | 70.6 | 73.7 | 71.5 | 69.9 | 100.0 |  |  |  |
| **WS9** | 78.6 |  |  |  |  | 80.8 | 74.5 |  | 100.0 |  |  |  | **WS9** | 69.9 | 72.0 | 72.6 | 71.2 | 73.3 | 70.7 | 69.4 | 77.1 | 100.0 |  |  |
| **WS11** | 79.6 | 68.3 | 69.7 | 73.5 | 76.4 | 76.0 |  |  | 77.2 | 100.0 |  |  | **WS11** | 67.7 | 68.7 | 69.7 | 74.0 | 74.4 | 74.9 | 67.4 | 73.1 | 78.1 | 100.0 |  |
| **OPL5** |  |  |  |  |  |  |  |  | 81.9 | 76.7 | 100.0 |  | **OPL5** | 61.1 | 60.2 | 62.4 | 61.5 | 63.3 | 59.7 | 59.7 | 60.6 | 60.2 | 60.8 | 100.0 |
|  |  |  |  |  |  |  |  |  |  |  |  |  |  |  |  |  |  |  |  |  |  |  |  |  |
|  |  |  |  |  |  |  |  |  |  |  |  |  |  |  |  |  |  |  |  |  |  |  |  |  |
| **isoC** | AD45 | PD630 | LB1 | WS7 | AT1 | i37 | OPL2 | WS12 | WS9 | WS11 | OPL5 |  | **isoI** | AD45 | PD630 | LB1 | WS7 | AT1 | i37 | OPL2 | WS12 | WS9 | WS11 | OPL5 |
| **AD45** | 100.0 |  |  |  |  |  |  |  |  |  |  |  | **AD45** | 100.0 |  |  |  |  |  |  |  |  |  |  |
| **PD630** | 86.1 | 100.0 |  |  |  |  |  |  |  |  |  |  | **PD630** | 84.2 | 100.0 |  |  |  |  |  |  |  |  |  |
| **LB1** | 86.1 | 100.0 | 100.0 |  |  |  |  |  |  |  |  |  | **LB1** | 77.0 | 80.8 | 100.0 |  |  |  |  |  |  |  |  |
| **WS7** | 83.2 | 87.5 | 87.5 | 100.0 |  |  |  |  |  |  |  |  | **WS7** | 81.6 | 87.7 | 75.9 | 100.0 |  |  |  |  |  |  |  |
| **AT1** | 74.0 | 75.9 | 75.9 | 73.3 | 100.0 |  |  |  |  |  |  |  | **AT1** | 78.5 | 82.9 | 75.5 | 80.1 | 100.0 |  |  |  |  |  |  |
| **i37** | 77.1 | 81.8 | 81.8 | 78.8 | 77.0 | 100.0 |  |  |  |  |  |  | **i37** | 78.9 | 81.9 | 73.9 | 79.9 | 78.8 | 100.0 |  |  |  |  |  |
| **OPL2** |  |  |  |  |  |  | 100.0 |  |  |  |  |  | **OPL2** | 78.0 | 81.1 | 74.9 | 79.2 | 80.3 | 87.1 | 100.0 |  |  |  |  |
| **WS12** | 70.3 | 75.8 | 75.8 | 73.7 | 75.0 | 74.0 |  | 100.0 |  |  |  |  | **WS12** | 72.7 | 73.5 | 71.7 | 71.2 | 71.6 | 73.7 | 72.3 | 100.0 |  |  |  |
| **WS9** | 78.5 | 71.2 | 71.2 | 77.4 | 72.8 | 69.9 |  |  | 100.0 |  |  |  | **WS9** | 70.2 | 66.4 | 68.6 | 68.7 | 70.7 | 69.4 | 65.2 | 73.8 | 100.0 |  |  |
| **WS11** | 70.3 | 71.2 | 71.1 | 72.9 | 71.8 | 76.6 |  |  | 78.7 | 100.0 |  |  | **WS11** | 67.5 | 66.1 | 67.9 | 65.8 | 65.1 | 65.2 | 67.1 | 74.9 | 80.2 | 100.0 |  |
| **OPL5** | 73.6 | 74.7 | 74.7 | 72.5 | 72.6 | 70.3 |  | 71.8 | 73.5 | 72.2 | 100.0 |  | **OPL5** | 72.4 |  |  | 66.2 | 65.7 | 64.3 | 67.3 | 67.0 | 67.7 | 68.1 | 100.0 |

| ***isoD*** | **AD45** | **PD630** | **LB1** | **WS7** | **AT1** | **i37** | **OPL2** | **WS12** | **WS9** | **WS11** | **OPL5** |  | ***isoJ*** | **AD45** | **PD630** | **LB1** | **WS7** | **AT1** | **i37** | **OPL2** | **WS12** | **WS9** | **WS11** | **OPL5** |
| --- | --- | --- | --- | --- | --- | --- | --- | --- | --- | --- | --- | --- | --- | --- | --- | --- | --- | --- | --- | --- | --- | --- | --- | --- |
| **AD45** | 100.0 |  |  |  |  |  |  |  |  |  |  |  | **AD45** | 100.0 |  |  |  |  |  |  |  |  |  |  |
| **PD630** | 89.5 | 100.0 |  |  |  |  |  |  |  |  |  |  | **PD630** | 74.8 | 100.0 |  |  |  |  |  |  |  |  |  |
| **LB1** | 89.5 | 100.0 | 100.0 |  |  |  |  |  |  |  |  |  | **LB1** | 75.0 | 97.9 | 100.0 |  |  |  |  |  |  |  |  |
| **WS7** | 87.1 | 92.8 | 92.8 | 100.0 |  |  |  |  |  |  |  |  | **WS7** | 73.5 | 89.5 | 90.3 | 100.0 |  |  |  |  |  |  |  |
| **AT1** | 77.2 | 79.1 | 79.1 | 77.1 | 100.0 |  |  |  |  |  |  |  | **AT1** | 72.6 | 78.1 | 78.6 | 77.8 | 100.0 |  |  |  |  |  |  |
| **i37** | 72.8 | 73.4 | 73.4 | 75.6 | 76.6 | 100.0 |  |  |  |  |  |  | **i37** | 70.3 | 72.7 | 72.7 | 73.2 | 69.5 | 100.0 |  |  |  |  |  |
| **OPL2** | 71.3 | 72.7 | 72.7 |  | 74.0 | 77.0 | 100.0 |  |  |  |  |  | **OPL2** |  |  |  |  |  |  | 100.0 |  |  |  |  |
| **WS12** | 73.8 | 71.8 | 71.8 |  | 70.9 | 70.0 | 70.9 | 100.0 |  |  |  |  | **WS12** |  |  |  |  |  |  |  | 100.0 |  |  |  |
| **WS9** | 65.7 | 67.3 | 67.3 | 67.0 | 71.7 | 69.1 | 65.6 |  | 100.0 |  |  |  | **WS9** | 67.7 | 67.3 | 68.1 | 67.8 | 66.8 | 72.3 |  |  | 100.0 |  |  |
| **WS11** |  | 69.2 | 69.2 | 67.4 | 71.7 | 69.5 | 69.0 | 69.7 | 81.0 | 100.0 |  |  | **WS11** | 70.6 | 70.5 | 71.2 | 71.0 | 69.1 | 77.0 |  |  | 78.1 | 100.0 |  |
| **OPL5** |  |  |  |  |  | 72.8 | 70.3 |  |  | 71.5 | 100.0 |  | **OPL5** |  | 64.9 | 65.3 | 65.8 | 68.2 | 66.9 |  |  | 68.8 | 66.3 | 100.0 |
|  |  |  |  |  |  |  |  |  |  |  |  |  |  |  |  |  |  |  |  |  |  |  |  |  |
|  |  |  |  |  |  |  |  |  |  |  |  |  |  |  |  |  |  |  |  |  |  |  |  |  |
|  |  |  |  |  |  |  |  |  |  |  |  |  |  |  |  |  |  |  |  |  |  |  |  |  |
| ***isoE*** | **AD45** | **PD630** | **LB1** | **WS7** | **AT1** | **i37** | **OPL2** | **WS12** | **WS9** | **WS11** | **OPL5** |  | ***aldH1*** | **AD45** | **PD630** | **LB1** | **WS7** | **AT1** | **i37** | **OPL2** | **WS12** | **WS9** | **WS11** | **OPL5** |
| **AD45** | 100.0 |  |  |  |  |  |  |  |  |  |  |  | **PD630** | - | 100.0 |  |  |  |  |  |  |  |  |  |
| **PD630** | 79.2 | 100.0 |  |  |  |  |  |  |  |  |  |  | **LB1** | - | 99.1 | 100.0 |  |  |  |  |  |  |  |  |
| **LB1** | 79.3 | 99.8 | 100.0 |  |  |  |  |  |  |  |  |  | **WS7** | - | 86.4 | 86.2 | 100.0 |  |  |  |  |  |  |  |
| **WS7** | 78.3 | 86.6 | 86.8 | 100.0 |  |  |  |  |  |  |  |  | **AT1** | - | 78.1 | 78.2 | 76.3 | 100.0 |  |  |  |  |  |  |
| **AT1** | 74.4 | 75.4 | 75.6 | 74.9 | 100.0 |  |  |  |  |  |  |  | **i37** | - | 76.4 | 76.3 | 75.7 | 73.6 | 100.0 |  |  |  |  |  |
| **i37** | 71.6 | 72.0 | 72.1 | 72.2 | 73.7 | 100.0 |  |  |  |  |  |  | **OPL2** | - | 73.7 | 73.5 | 71.4 | 71.6 | 77.2 | 100.0 |  |  |  |  |
| **OPL2** | 68.5 | 71.4 | 71.6 | 72.6 | 71.1 | 75.9 | 100.0 |  |  |  |  |  | **WS12** | - | 71.8 | 71.6 | 70.9 | 69.6 | 70.1 | 68.9 | 100.0 |  |  |  |
| **WS12** | 69.0 | 70.8 | 71.1 | 68.4 | 69.0 | 71.4 | 67.6 | 100.0 |  |  |  |  | **WS9** | - | 67.7 | 67.5 | 65.6 | 67.6 | 68.0 | 65.2 | 69.7 | 100.0 |  |  |
| **WS9** | 66.2 | 66.2 | 66.0 | 65.4 | 66.5 | 68.0 | 64.8 | 66.5 | 100.0 |  |  |  | **WS11** | - | 67.3 | 67.3 | 70.0 | 69.7 | 71.9 | 67.4 | 68.5 | 78.5 | 100.0 |  |
| **WS11** | 67.1 | 71.0 | 70.6 | 73.9 | 65.6 | 67.8 | 72.0 | 66.2 | 73.7 | 100.0 |  |  | **OPL5** | - | 66.3 | 66.6 | 64.1 | 65.2 | 65.0 | 64.7 | 68.1 | 66.7 | 67.2 | 100.0 |

| ***isoF*** | **AD45** | **PD630** | **LB1** | **WS7** | **AT1** | **i37** | **OPL2** | **WS12** | **WS9** | **WS11** | **OPL5** |
| --- | --- | --- | --- | --- | --- | --- | --- | --- | --- | --- | --- |
| **AD45** | 100.0 |  |  |  |  |  |  |  |  |  |  |
| **PD630** | 79.6 | 100.0 |  |  |  |  |  |  |  |  |  |
| **LB1** | 79.6 | 100.0 | 100.0 |  |  |  |  |  |  |  |  |
| **WS7** | 75.3 | 85.6 | 85.6 | 100.0 |  |  |  |  |  |  |  |
| **AT1** | 67.1 | 69.1 | 69.1 | 68.4 | 100.0 |  |  |  |  |  |  |
| **i37** | 65.8 | 66.9 | 66.9 | 67.2 | 66.2 | 100.0 |  |  |  |  |  |
| **OPL2** | 68.0 | 67.3 | 67.3 | 66.0 | 68.8 | 71.3 | 100.0 |  |  |  |  |
| **WS12** | 74.3 | 80.9 | 80.9 | 70.5 | 72.6 | 64.7 | 94.4 | 100.0 |  |  |  |
| **WS9** | 95.0 | 88.9 | 88.9 | 71.3 | 72.9 | 69.5 |  |  | 100.0 |  |  |
| **WS11** | 72.0 | 77.5 | 77.5 | 80.3 | 72.9 | 75.2 | 81.1 | 80.7 | 70.9 | 100.0 |  |
| **OPL5** | 74.3 | 70.3 | 70.3 | 100.0 | 70.0 | 67.3 | 69.4 | 79.6 | 72.2 | 85.5 | 100.0 |

|  |  |  |  |  |  |  |  |  |  |  |  |  |  |  |  |  |  |  |  |  |  |  |  |  |
| --- | --- | --- | --- | --- | --- | --- | --- | --- | --- | --- | --- | --- | --- | --- | --- | --- | --- | --- | --- | --- | --- | --- | --- | --- |
|  |  |  |  |  |  |  |  |  |  |  |  |  |  |  |  |  |  |  |  |  |  |  |  |  |
|  |  |  |  |  |  |  |  |  |  |  |  |  |  |  |  |  |  |  |  |  |  |  |  |  |
|  |  |  |  |  |  |  |  |  |  |  |  |  |  |  |  |  |  |  |  |  |  |  |  |  |
|  |  |  |  |  |  |  |  |  |  |  |  |  |  |  |  |  |  |  |  |  |  |  |  |  |
|  |  |  |  |  |  |  |  |  |  |  |  |  |  |  |  |  |  |  |  |  |  |  |  |  |
|  |  |  |  |  |  |  |  |  |  |  |  |  |  |  |  |  |  |  |  |  |  |  |  |  |
|  |  |  |  |  |  |  |  |  |  |  |  |  |  |  |  |  |  |  |  |  |  |  |  |  |
|  |  |  |  |  |  |  |  |  |  |  |  |  |  |  |  |  |  |  |  |  |  |  |  |  |
|  |  |  |  |  |  |  |  |  |  |  |  |  |  |  |  |  |  |  |  |  |  |  |  |  |
|  |  |  |  |  |  |  |  |  |  |  |  |  |  |  |  |  |  |  |  |  |  |  |  |  |
|  |  |  |  |  |  |  |  |  |  |  |  |  |  |  |  |  |  |  |  |  |  |  |  |  |
|  |  |  |  |  |  |  |  |  |  |  |  |  |  |  |  |  |  |  |  |  |  |  |  |  |
|  |  |  |  |  |  |  |  |  |  |  |  |  |  |  |  |  |  |  |  |  |  |  |  |  |

|  |  |  |  |  |  |  |  |  |  |  |  |  |  |  |  |  |  |  |  |  |  |  |  |  |
| --- | --- | --- | --- | --- | --- | --- | --- | --- | --- | --- | --- | --- | --- | --- | --- | --- | --- | --- | --- | --- | --- | --- | --- | --- |
|  |  |  |  |  |  |  |  |  |  |  |  |  |  |  |  |  |  |  |  |  |  |  |  |  |
|  |  |  |  |  |  |  |  |  |  |  |  |  |  |  |  |  |  |  |  |  |  |  |  |  |
|  |  |  |  |  |  |  |  |  |  |  |  |  |  |  |  |  |  |  |  |  |  |  |  |  |
|  |  |  |  |  |  |  |  |  |  |  |  |  |  |  |  |  |  |  |  |  |  |  |  |  |
|  |  |  |  |  |  |  |  |  |  |  |  |  |  |  |  |  |  |  |  |  |  |  |  |  |
|  |  |  |  |  |  |  |  |  |  |  |  |  |  |  |  |  |  |  |  |  |  |  |  |  |
|  |  |  |  |  |  |  |  |  |  |  |  |  |  |  |  |  |  |  |  |  |  |  |  |  |
|  |  |  |  |  |  |  |  |  |  |  |  |  |  |  |  |  |  |  |  |  |  |  |  |  |
|  |  |  |  |  |  |  |  |  |  |  |  |  |  |  |  |  |  |  |  |  |  |  |  |  |
|  |  |  |  |  |  |  |  |  |  |  |  |  |  |  |  |  |  |  |  |  |  |  |  |  |
|  |  |  |  |  |  |  |  |  |  |  |  |  |  |  |  |  |  |  |  |  |  |  |  |  |
|  |  |  |  |  |  |  |  |  |  |  |  |  |  |  |  |  |  |  |  |  |  |  |  |  |
|  |  |  |  |  |  |  |  |  |  |  |  |  |  |  |  |  |  |  |  |  |  |  |  |  |
|  |  |  |  |  |  |  |  |  |  |  |  |  |  |  |  |  |  |  |  |  |  |  |  |  |
|  |  |  |  |  |  |  |  |  |  |  |  |  |  |  |  |  |  |  |  |  |  |  |  |  |
|  |  |  |  |  |  |  |  |  |  |  |  |  |  |  |  |  |  |  |  |  |  |  |  |  |
|  |  |  |  |  |  |  |  |  |  |  |  |  |  |  |  |  |  |  |  |  |  |  |  |  |
|  |  |  |  |  |  |  |  |  |  |  |  |  |  |  |  |  |  |  |  |  |  |  |  |  |
|  |  |  |  |  |  |  |  |  |  |  |  |  |  |  |  |  |  |  |  |  |  |  |  |  |
|  |  |  |  |  |  |  |  |  |  |  |  |  |  |  |  |  |  |  |  |  |  |  |  |  |
|  |  |  |  |  |  |  |  |  |  |  |  |  |  |  |  |  |  |  |  |  |  |  |  |  |
|  |  |  |  |  |  |  |  |  |  |  |  |  |  |  |  |  |  |  |  |  |  |  |  |  |
|  |  |  |  |  |  |  |  |  |  |  |  |  |  |  |  |  |  |  |  |  |  |  |  |  |
|  |  |  |  |  |  |  |  |  |  |  |  |  |  |  |  |  |  |  |  |  |  |  |  |  |
|  |  |  |  |  |  |  |  |  |  |  |  |  |  |  |  |  |  |  |  |  |  |  |  |  |

|  |  |  |  |  |  |  |  |  |  |  |  |  |  |  |  |  |  |  |  |  |  |  |  |  |
| --- | --- | --- | --- | --- | --- | --- | --- | --- | --- | --- | --- | --- | --- | --- | --- | --- | --- | --- | --- | --- | --- | --- | --- | --- |
|  |  |  |  |  |  |  |  |  |  |  |  |  |  |  |  |  |  |  |  |  |  |  |  |  |
|  |  |  |  |  |  |  |  |  |  |  |  |  |  |  |  |  |  |  |  |  |  |  |  |  |
|  |  |  |  |  |  |  |  |  |  |  |  |  |  |  |  |  |  |  |  |  |  |  |  |  |
|  |  |  |  |  |  |  |  |  |  |  |  |  |  |  |  |  |  |  |  |  |  |  |  |  |
|  |  |  |  |  |  |  |  |  |  |  |  |  |  |  |  |  |  |  |  |  |  |  |  |  |
|  |  |  |  |  |  |  |  |  |  |  |  |  |  |  |  |  |  |  |  |  |  |  |  |  |
|  |  |  |  |  |  |  |  |  |  |  |  |  |  |  |  |  |  |  |  |  |  |  |  |  |
|  |  |  |  |  |  |  |  |  |  |  |  |  |  |  |  |  |  |  |  |  |  |  |  |  |
|  |  |  |  |  |  |  |  |  |  |  |  |  |  |  |  |  |  |  |  |  |  |  |  |  |
|  |  |  |  |  |  |  |  |  |  |  |  |  |  |  |  |  |  |  |  |  |  |  |  |  |
|  |  |  |  |  |  |  |  |  |  |  |  |  |  |  |  |  |  |  |  |  |  |  |  |  |
|  |  |  |  |  |  |  |  |  |  |  |  |  |  |  |  |  |  |  |  |  |  |  |  |  |
|  |  |  |  |  |  |  |  |  |  |  |  |  |  |  |  |  |  |  |  |  |  |  |  |  |
|  |  |  |  |  |  |  |  |  |  |  |  |  |  |  |  |  |  |  |  |  |  |  |  |  |
|  |  |  |  |  |  |  |  |  |  |  |  |  |  |  |  |  |  |  |  |  |  |  |  |  |
|  |  |  |  |  |  |  |  |  |  |  |  |  |  |  |  |  |  |  |  |  |  |  |  |  |
|  |  |  |  |  |  |  |  |  |  |  |  |  |  |  |  |  |  |  |  |  |  |  |  |  |
|  |  |  |  |  |  |  |  |  |  |  |  |  |  |  |  |  |  |  |  |  |  |  |  |  |
|  |  |  |  |  |  |  |  |  |  |  |  |  |  |  |  |  |  |  |  |  |  |  |  |  |
|  |  |  |  |  |  |  |  |  |  |  |  |  |  |  |  |  |  |  |  |  |  |  |  |  |
|  |  |  |  |  |  |  |  |  |  |  |  |  |  |  |  |  |  |  |  |  |  |  |  |  |
|  |  |  |  |  |  |  |  |  |  |  |  |  |  |  |  |  |  |  |  |  |  |  |  |  |
|  |  |  |  |  |  |  |  |  |  |  |  |  |  |  |  |  |  |  |  |  |  |  |  |  |
|  |  |  |  |  |  |  |  |  |  |  |  |  |  |  |  |  |  |  |  |  |  |  |  |  |
